# Serpina3c protects against metabolic dysfunction-associated steatotic liver disease in offspring induced by prenatal prednisone exposure

**DOI:** 10.1038/s41392-025-02569-1

**Published:** 2026-02-18

**Authors:** Yongguo Dai, Zhengjie Lu, Yu Peng, Kexin Liu, Xiaoqian Lu, Xiaoxiang Sun, Yuxi Wang, Xuerong Yan, Zijie Chen, Ziyi Zhang, Ning Zhang, Aihemaitijiang Ailikaiti, Yiming Chen, Quanrui Yue, Yu Guo, Liaobin Chen, Hui Wang

**Affiliations:** 1 Department of Pharmacology, Wuhan University School of Basic Medical Sciences, Wuhan, Hubei Province China; 2 Department of Pharmacy, National Clinical Research Center for Geriatric Disorders, Xiangya Hospital of Central South University, Changsha, Hunan Province China; 3 Department of Orthopedic Surgery, Joint Disease Research Center of Wuhan University, Zhongnan Hospital of Wuhan University, Wuhan, Hubei Province China; 4 Hubei Provincial Key Laboratory of Developmentally Originated Disease, Wuhan, Hubei Province China

**Keywords:** Metabolic disorders, Endocrine system and metabolic diseases

## Abstract

Metabolic dysfunction-associated steatotic liver disease (MASLD) has become a major global public health problem, and its occurrence is associated with adverse environmental exposures during development. In this study, we investigated the impact of the use of prednisone (a synthetic glucocorticoid drug) during pregnancy on susceptibility to MASLD in offspring and explored its potential therapeutic targets. Pregnant rodents were administered clinically equivalent doses of prednisone daily by oral gavage during gestation days (GDs) 0–20 in rats and GD0–18 mice, and their offspring were fed a high-fat diet from postnatal weeks 8–12. The results showed that prenatal prednisone exposure (PPE) led to reduced hepatic glucose uptake and fatty acid oxidation in offspring rats prenatally and postnatally and that the offspring developed more severe MASLD when fed a high-fat diet, with males exhibiting greater severity than females. Consistent findings were observed in PPE adult offspring mice. RNA-seq and experimental results revealed that hepatic Serpina3c expression was consistently reduced in PPE offspring before and after birth, which led to an increase in chymase-Ang II production and subsequent activation of its receptor AT1R, leading to MASLD susceptibility. In vivo and in vitro studies revealed that the programming of low Serpina3c expression was associated with reduced H3K27ac levels in the gene promoter region of Serpina3c caused by the activation of GR-HDAC3 signaling by the active metabolite prednisolone. Finally, postnatal high expression of hepatic Serpina3c reversed the activation of the chymase-Ang II-AT1R pathway and significantly ameliorated hepatic glucose and lipid metabolic dysfunction and MASLD susceptibility in PPE offspring. In summary, this study reveals MASLD susceptibility in offspring induced by PPE and identifies Serpina3c as a target for the prevention and treatment of MASLD susceptibility.

## Introduction

Oral glucocorticoid drugs are used to manage symptoms of chronic diseases such as rheumatoid arthritis, systemic lupus erythematosus, and other autoimmune disorders. It is estimated that 1–2% of pregnant women and 20–40% of pregnant women with rheumatoid arthritis use oral glucocorticoids.^1–5^ Prednisone is the most commonly used oral glucocorticoid during pregnancy.^1,3^ A prospective cohort study of rheumatoid arthritis patients during pregnancy revealed that prednisone comprised 98% of all oral glucocorticoid prescriptions.^6^ In addition, prednisone is employed in assisted reproductive technologies worldwide to enhance embryo implantation and prevent miscarriage.^7,8^ Therefore, prednisone use during gestation may be clinically unavoidable for managing maternal disease. Although some studies suggest no elevated risk of adverse pregnancy outcomes,^9,10^ studies published in journals such as *JAMA* have identified associations between the use of prednisone during pregnancy and increased risks of infections, preterm birth, biochemical pregnancy loss, and offspring oral clefts.^6,11–13^ Furthermore, a study in *Science* showed that prenatal prednisone exposure (PPE) in both humans and animals caused intrauterine growth retardation, which specifically attributed the reduced birth weight to prednisone exposure rather than to maternal pathology.^14^ Animal studies from our laboratory and others have also shown that PPE can lead to abnormalities in the development and function of the offspring’s brain, kidneys, articular cartilage, and reproductive organs, among others.^15–23^ Therefore, an in-depth understanding of the adverse effects of prednisone use during pregnancy on offspring is essential for guiding the clinical use of the drug and managing the long-term health of offspring.

Metabolic dysfunction-associated steatotic liver disease (MASLD), previously termed nonalcoholic fatty liver disease (NAFLD), is currently defined as “steatotic liver disease (SLD) in the presence of one or more cardiometabolic risk factor(s) and the absence of harmful alcohol intake”.^24,25^ The term MASLD comprises different conditions, including isolated liver steatosis (metabolic dysfunction-associated steatotic liver, MASL), metabolic dysfunction-associated steatohepatitis (MASH, previously NASH), as well as fibrosis, cirrhosis, and MASH-related hepatocellular carcinoma. MASLD has become one of the most common chronic liver diseases worldwide, affecting more than 30% of the global population.^26–29^ The global prevalence of MASLD is forecast to reach 55.4% by 2040.^30^ To date, only resmetirom and semaglutide have been approved by the U.S. Food and Drug Administration (FDA) for the treatment of MASLD. Therefore, gaining a better understanding of the precise etiology of MASLD may contribute to the development of promising therapeutic approaches for this condition. Growing evidence supports the developmental origin of MASLD.^31^ During intrauterine development, an adverse environment can lead to increased susceptibility to disease later in an individual’s life. This phenomenon is now known as developmental origins of health and disease (DOHaD) and has been extended to a variety of diseases, including MASLD.^32^ Epidemiological studies have shown that maternal use of prednisone during pregnancy is followed by the birth of offspring with low birth weights,^14,33^, which is strongly associated with the development of disorders related to glucose and lipid metabolism disorders, including MASLD.^34–36^ Our recent results have also shown that liver development, as well as glucose and lipid metabolism functions in fetal mice are affected by PPE.^21^ Therefore, we hypothesize that PPE may increase susceptibility to MASLD in offspring.

Epigenetic modifications refer to heritable alterations in gene expression that do not involve alterations to the DNA sequence. The primary mechanisms include DNA methylation, histone modifications, and noncoding RNA-associated regulation. Both animal and population studies have demonstrated that epigenetic mechanisms contribute significantly to the pathogenesis of MASLD.^37–40^ Epigenetic modification alterations can be long-lasting and even transferable to the next generation, thereby altering the risk of disease in offspring.^41^ The role of epigenetic mechanisms in mediating the developmental programming of MASLD induced by adverse early-life exposures, particularly in utero, is well established.^42–54^ For example, alterations in histone modifications have been demonstrated to be a significant cause of the increase in offspring MASLD risk associated with various adverse factors during pregnancy, including maternal disease, nutritional changes, and exposure to exogenous substances.^43–54^This may be a result of epigenetic alterations programming fetal liver development and function, thereby increasing its susceptibility to MASLD after birth.^42^ Therefore, we propose that PPE programs offspring liver development and susceptibility to MASLD through epigenetic modifications.

This study aimed to investigate the effects of PPE on hepatic metabolic functions and susceptibility to MASLD in offspring. On the basis of the commonly used treatment regimens of prednisone in the population, animal (rat and mouse) models of PPE were established according to our previous studies,^23,55^ as shown in Supplementary Figs. 1 and 2. We subsequently observed the hepatic glucose and lipid metabolic functions before and after birth, as well as the MASLD phenotypes in offspring. We further sought to elucidate the underlying intrauterine programming mechanisms and identify potential therapeutic targets. This research provides valuable insights into the developmental toxicity of PPE to offspring and offers an experimental foundation for developing strategies to mitigate its adverse effects on offspring.

## Results

### PPE induces alterations in glucose and lipid metabolism and increases susceptibility to MASLD in offspring

**Fig. 1:**
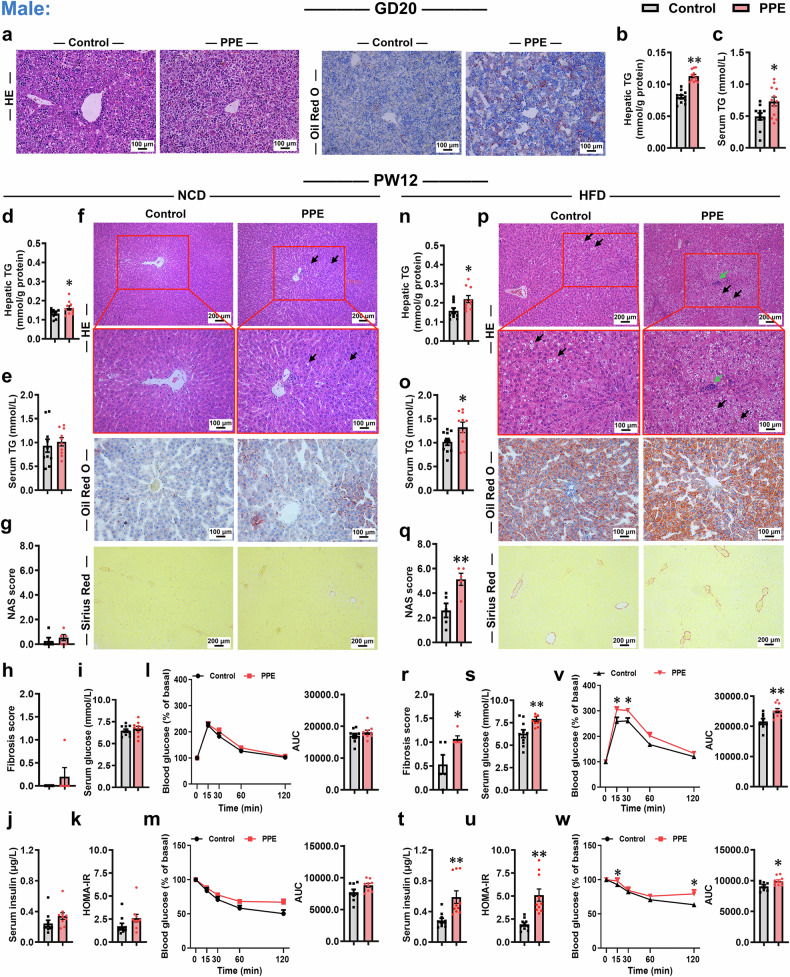
PPE increased susceptibility to MASLD in male offspring rats. Pregnant Wistar rats were intragastrically administered prednisone (0.25 mg/kg) or vehicle control (0.5% CMC-Na) per day from GD0−20, and offspring rats of different ages (GD20 and PW12) were subsequently obtained for further analysis. Among these offspring, some were fed a HFD (D12492) from PW8−12. **a** Representative micrographs of liver sections stained with HE and oil red O staining at GD20, scale bar: 100 μm; **b**, **c** Hepatic and serum TG levels at GD20; **d**, **n** Hepatic TG content at PW12; **e**, **o** Serum TG levels at PW12; **f**, **p** Representative micrographs of liver sections stained with HE, oil red O and Sirius red staining at PW12, scale bar: 100 μm and 200 μm; **g**, **q** Liver NAS score at PW12; **h**, **r** Liver fibrosis score at PW12; (**i**−**k**, **s**−**u**) Fasting serum glucose, insulin and HOMA-IR index at PW12; (**l**, **v**) Normalized blood glucose levels during the IPGTT and corresponding AUC at PW12B; (**m**, **w**) Normalized blood glucose levels during the IPITT and corresponding AUC at PW12. Mean ± SEM, n = 11−12 for hepatic and serum TG levels on GD20, n = 5 for histopathological data, n = 10 for other data. Statistical significance was determined by two-tailed unpaired Student’s *t* test (**a**−**s**, **v**, **w**), nonparametric test (Mann‒Whitney U test) (**t**, **u**), and two-way ANOVA for repeated measures followed by Bonferroni post hoc correction (**i**, **m**, **v**, **w**). ^*^P < 0.05, ^**^P < 0.01 *vs*. control. AUC area under the curve, CMC-Na carboxymethyl cellulose sodium, GD gestational day, HE hematoxylin and eosin, HFD high-fat diet, HOMA-IR homeostatic model assessment of insulin resistance, IPGTT intraperitoneal glucose tolerance test, IPITT intraperitoneal insulin tolerance test, NCD normal chow diet, PPE prenatal prednisone exposure, PW postnatal week, TG triglyceride

**Fig. 2:**
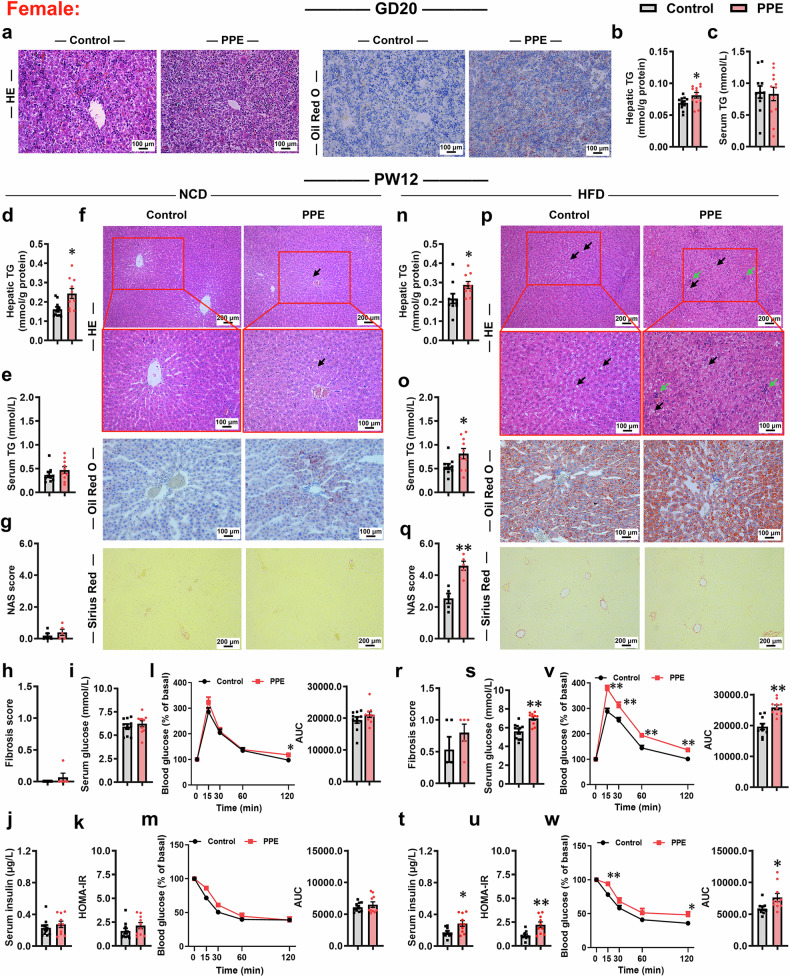
PPE increased susceptibility to MASLD in female offspring rats. Pregnant Wistar rats were intragastrically administered prednisone (0.25 mg/kg) or vehicle control (0.5% CMC-Na) per day from GD0−20, and offspring rats of different ages (GD20 and PW12) were subsequently obtained for further analysis. Among these offspring, some were fed a HFD (D12492) from PW8−12. **a** Representative micrographs of liver sections stained with HE and oil red O staining on GD20, scale bar: 100 μm; **b**, **c** Hepatic and serum TG levels on GD20; **d**, **n** Hepatic TG content at PW12; **e**, **o** Serum TG levels at PW12; **f**, **p** Representative micrographs of liver sections stained with HE, oil red O and Sirius red staining at PW12, scale bar: 100 μm and 200 μm; **g**, **q** Liver NAS score at PW12; **h**, **r** Liver fibrosis score at PW12; **i**−**k**, **s**−**u** Fasting serum glucose, insulin and HOMA-IR index at PW12; **l**, **v** Normalized blood glucose levels during the IPGTT and corresponding AUC at PW12; **m**, **w** Normalized blood glucose levels during the IPITT and corresponding AUC at PW12. Mean ± SEM, n = 11−12 for hepatic and serum TG levels on GD20, n = 5 for histopathological data, n = 10 for other data. Statistical significance was determined by two-tailed unpaired Student’s *t* test (**a**−**w**) and two-way ANOVA for repeated measures followed by Bonferroni post hoc correction (**l**, **m**, **v**, **w**). ^*^P < 0.05, ^**^P < 0.01 *vs*. control. AUC area under the curve, CMC-Na carboxymethyl cellulose sodium, GD gestational day, HE hematoxylin and eosin, HFD high-fat diet, HOMA-IR homeostatic model assessment of insulin resistance, IPGTT intraperitoneal glucose tolerance test, IPITT intraperitoneal insulin tolerance test, NCD normal chow diet, PPE prenatal prednisone exposure, PW postnatal week, TG triglyceride

We first investigated the effects of PPE on growth and development as well as susceptibility to MASLD in both male and female offspring. Compared with the control group, PPE reduced maternal weight gain, significantly inhibited intrauterine growth in both male and female fetal rats, and induced pronounced catch-up growth in female offspring rats (Supplementary Fig. 3). On gestation day (GD) 20, PPE significantly decreased fetal liver weights in both sexes, but the liver weight/body weight ratio showed no discernible change (Supplementary Figs. 4a, 4b, g, h). PPE also induced evident fatty vacuolar degeneration and lipid accumulation (Figs. 1a and 2a). Concurrently, PPE markedly increased the hepatic triglyceride (TG) content in both sexes and elevated the serum TG level in male fetal rats (Figs. 1b, c and 2b), whereas no significant alteration in the female fetal serum TG level was detected (Fig. 2c). Although PPE notably reduced fetal serum insulin levels, it did not significantly affect hepatic glycogen content or serum glucose levels in either sex (Supplementary Fig. 4c−e, i−k). These findings suggested that PPE disrupted hepatic TG metabolism in fetal rats. In postnatal week (PW) 12, under normal chow diet (NCD) feeding conditions, compared with those in the control group, both male and female offspring rats in the PPE group presented increased hepatic TG content and mild Oil Red O-positive staining, whereas other phenotypic indicators presented no significant alterations (Figs. 1d−m, 2d−m; Supplementary Fig. 4m, n, p, q). When the offspring rats were challenged with a high-fat diet (HFD), more pronounced phenotypic alterations in fatty liver were observed in both male and female offspring rats in the PPE group (Figs. 1n−r, 2n−r; Supplementary Fig. 4s, t, v, w). Specifically, male offspring rats in the PPE group presented significantly increased liver weights, liver weight/body weight ratios, hepatic and serum TG levels, and liver NAFLD activity score (NAS), as well as more pronounced hepatic steatosis, lipid accumulation, inflammatory infiltration, and collagen fiber deposition (Fig. 1n−r; Supplementary Fig. 4s, t); female offspring rats in the PPE group presented patterns similar to those of males except that increases in liver weights, liver weight/body weight ratios, and collagen fiber deposition were not observed (Fig. 2n−r and Supplementary Fig. 4v, w). Moreover, impaired systemic glucose metabolic homeostasis was observed in PPE male and female offspring rats fed a HFD (Fig. 1s−w, 2s−w). Consistent findings were observed in PPE adult offspring mice (Supplementary Figs. 5 and 6). These results collectively indicate that PPE enhances susceptibility to MASLD in offspring, with a more severe effect in males than in females.

**Fig. 3:**
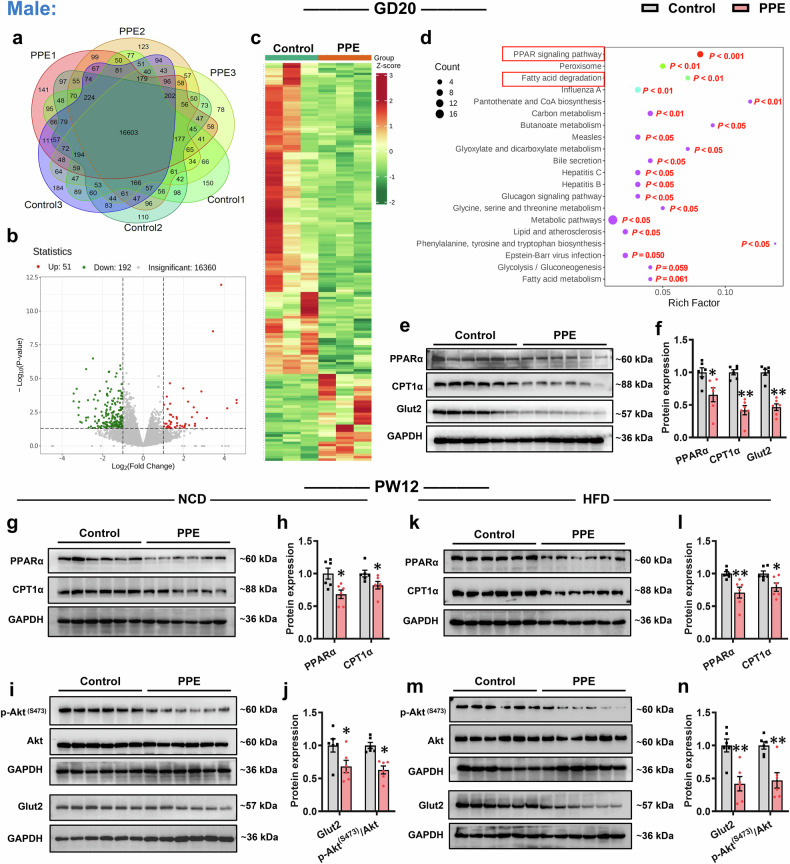
PPE altered hepatic glucose and lipid metabolism in male offspring rats. Pregnant Wistar rats were intragastrically administered prednisone (0.25 mg/kg) or vehicle control (0.5% CMC-Na) per day from GD0−20, and offspring rats of different ages (GD20 and PW12) were subsequently obtained for further analysis. Among these offspring, some were fed a HFD (D12492) from PW8−12. **a** Venn diagram showing the expression of genes detected in each sample from the liver on GD20; **b** Volcano plot showing DEGs in the liver on GD20 when |log_2_FC| > 1 and *P* < 0.05; **c** Hot map showing DEGs in the liver on GD20; **d** KEGG enrichment pathway analysis of DEGs in the liver on GD20; **e**, **f** Representative WB images and semiquantitative results of hepatic PPARα, CPT1α and Glut2 protein expression on GD20; **g**−**n** Representative WB images and semiquantitative results of hepatic PPARα, CPT1α, Glut2, Akt and p-Akt ^(S473)^ protein expression in PW12. Mean ± SEM, n = 3 for RNA-seq, n = 6 for WB. Statistical significance was determined via the DESeq2 R package (1.20.0) (**a**−**d**) and two-tailed unpaired Student’s *t* test (**e**−**n**). ^*^P < 0.05, ^**^P < 0.01 *vs*. control. Akt protein kinase B, CPT1α carnitine palmitoyltransferase 1α; CMC-Na carboxymethyl cellulose sodium, GAPDH glyceraldehyde-3-phosphate dehydrogenase, Glut2 glucose transporter 2, GD gestational day, HFD high-fat diet, p-Akt phospho-Akt, PPARα peroxisome proliferator-activated receptor α, PPE prenatal prednisone exposure, PW postnatal week, NCD normal chow diet, WB western blot

**Fig. 4:**
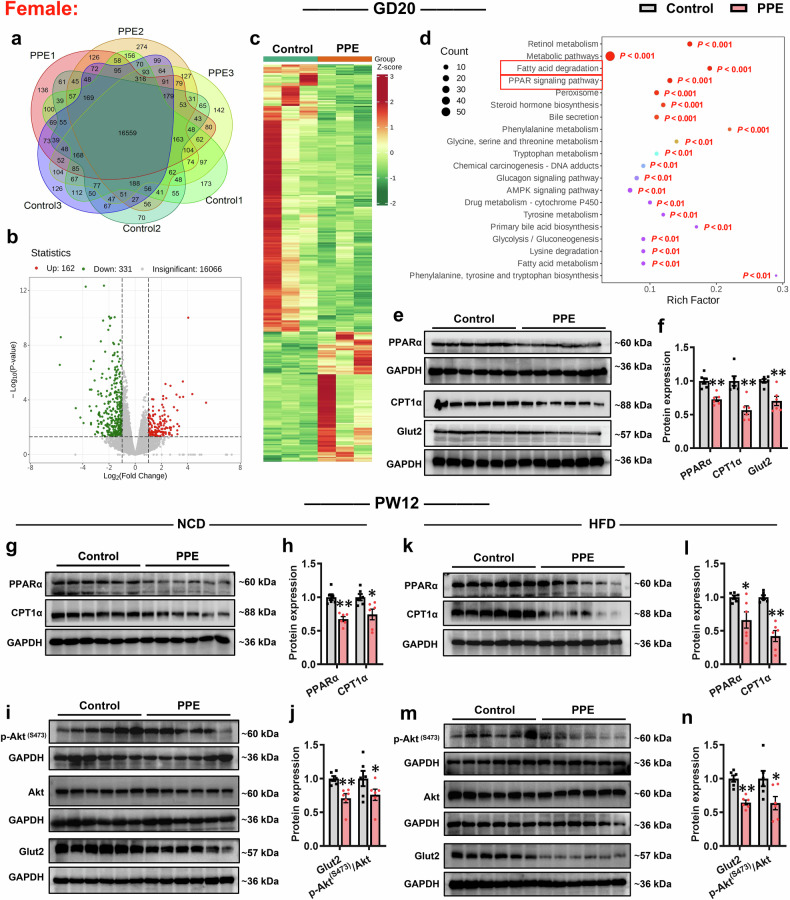
PPE altered hepatic glucose and lipid metabolism in female offspring rats. Pregnant Wistar rats were intragastrically administered prednisone (0.25 mg/kg) or vehicle control (0.5% CMC-Na) per day from GD0−20, and offspring rats of different ages (GD20 and PW12) were subsequently obtained for further analysis. Among these offspring, some were fed an HFD (D12492) from PW8−12. **a** Venn diagram showing the expression of genes detected in each sample from the liver on GD20; **b** Volcano plot showing DEGs in the liver on GD20 when |log_2_FC| > 1 and *P* < 0.05; **c** Hot map showing DEGs in the liver on GD20; **d** KEGG enrichment pathway analysis of DEGs in the liver on GD20; **e**, **f** Representative WB images and semiquantitative results of hepatic PPARα, CPT1α and Glut2 protein expression on GD20; (**g**−**n**) Representative WB images and semiquantitative results of hepatic PPARα, CPT1α, Glut2, Akt and p-Akt ^(S473)^ protein expression in PW12. Mean ± SEM, n = 3 for RNA-seq, n = 6 for WB. Statistical significance was determined via the DESeq2 R package (1.20.0) (**a**−**d**) and two-tailed unpaired Student’s *t* test (**e**−**n**). ^*^P < 0.05, ^**^P < 0.01 *vs*. control. Akt protein kinase B, CPT1α carnitine palmitoyltransferase 1α, CMC-Na carboxymethyl cellulose sodium, GAPDH glyceraldehyde-3-phosphate dehydrogenase, Glut2 glucose transporter 2, GD gestational day, HFD high-fat diet, p-Akt phospho-Akt, PPARα peroxisome proliferator-activated receptor α, PPE prenatal prednisone exposure, PW postnatal week, NCD normal chow diet, WB western blot

We further examined functional changes in hepatic glucose and lipid metabolism in PPE male and female offspring. Messenger RNA sequencing (mRNA-seq) analysis revealed that 16,603 and 16,559 genes were detected in all male and female rat fetal liver samples from the control and PPE groups, respectively (Figs. 3a and 4a; Supplementary Fig. 7). Differential expression analysis (|log2FC| > 1, *P* < 0.05) demonstrated that, compared with the control group, the PPE group presented 51 upregulated and 192 downregulated genes in the male fetal liver (Fig. 3b, c), with 162 upregulated and 331 downregulated genes in the female fetal liver (Fig. 4b, c). Kyoto Encyclopedia of Genes and Genomes (KEGG) functional enrichment analysis indicated that these differentially expressed genes (DEGs) in the fetal livers of both sexes in the PPE group were associated mainly with lipid metabolism pathways, particularly the PPAR signaling pathway and fatty acid degradation (Figs. 3d and 4d). RT‒qPCR confirmed that the fetal livers of both sexes in the PPE group collectively presented significantly reduced mRNA expression of key genes involved in fatty acid oxidation and glucose uptake [peroxisome proliferators activated receptor α (*Ppara*), carnitine palmitoyltransferase 1α (*Cpt1a*), enoyl-CoA hydratase and 3-hydroxyacyl CoA dehydrogenase (*Ehhadh*), Cytochrome P450 4a8(*Cyp4a8)*, and glucose transporter 2 (*Glut2*)] (Supplementary Fig. 4f, l). The western blot (WB) results further confirmed the markedly decreased protein levels of PPARα, CPT1α, and Glut2 in the PPE male and female fetal rat livers (Figs. 3e, f and 4e, f). These reductions in gene and protein expression persisted in the livers of adult PPE male and female offspring rats under both NCD and HFD conditions, accompanied by significant suppression of hepatic insulin signaling pathways (Figs. 3g−n and 4g−n). The above findings suggest that the common mechanism underlying PPE-induced susceptibility to MASLD in offspring may be associated with hepatic metabolic dysfunction caused by impaired fatty acid oxidation and glucose uptake capacity.

Notably, a marked increase in the expression of sterol-regulatory element binding protein-1(*Srebp1*), fatty acid synthase (*Fasn*), and microsomal triglyceride transfer protein (*Mttp*) was observed specifically in PPE male offspring rats (Supplementary Fig. 4f), indicating increased de novo fatty acid synthesis and lipid output in the liver. In females, PPE significantly reduced the hepatic expression of glucokinase (*Gck*) and phosphoenolpyruvate carboxykinase 1 (*Pck1*), which encode key glycolytic and gluconeogenic enzymes, respectively (Supplementary Fig. 4l). These results indicated that PPE also induced sex-specific alterations in hepatic glucose and lipid metabolism in male and female offspring.

### Low expression of Serpina3c mediates PPE-induced MASLD susceptibility in offspring

**Fig. 5:**
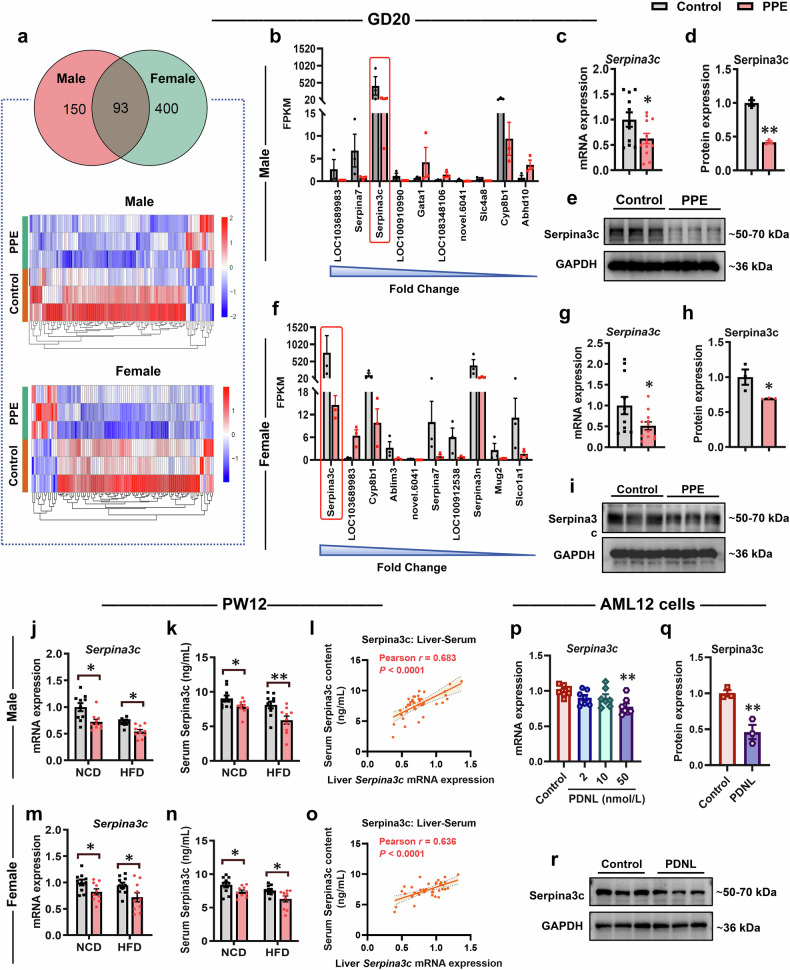
PPE inhibited hepatic Serpina3c expression in both male and female offspring rats. Pregnant Wistar rats were intragastrically administered prednisone (0.25 mg/kg) or vehicle control (0.5% CMC-Na) per day from GD0−20, and offspring rats of different ages (GD20 and PW12) were subsequently obtained for further analysis. Among these offspring, some were fed a HFD (D12492) from PW8−12. AML12 cells were treated with different concentrations (0, 2, 10, or 50 nmol/L) of prednisolone for 72 h and then harvested for further analysis. **a** The number of common DEGs and their heatmap in the livers of both male and female fetal rats on GD20; (**b**, **f**) Top 10 genes ranked for common DEGs in the livers of both male and female fetal rats on GD20; **c**, **g** *Serpina3c* mRNA expression in the livers of both male and female fetal rats on GD20; **d**, **e**, **h**, **i** Representative WB images and semiquantitative results of Serpina3c protein expression in the livers of both male and female fetal rats on GD20; **j**, **m** *Serpina3c* mRNA expression in the livers of NCD-fed and HFD-fed male and female offspring rats on PW12; **k**, **n** Serum Serpina3c content in NCD-fed male and female offspring rats on PW12; **l**, **o** Pearson correlation analysis of serum Serpina3c content and hepatic *Serpina3c* mRNA expression in male and female offspring rats on PW12; **p** *Serpina3c* mRNA expression in AML12 cells; **q**, **r** Representative WB images and semiquantitative results of Serpina3c protein expression in AML12 cells. Mean ± SEM, n = 11−12 for RT-qRCR on GD20, n = 3 for RNA-seq and WB data, n = 10 for other data. Statistical significance was determined via the DESeq2 R package (1.20.0) (**a**, **b**, **f**), two-tailed unpaired Student’s *t* test (c−e, g−k, m, n, q, r), one-way ANOVA with Dunnett’s post hoc test (**p**), and Pearson correlation analysis (**l**, **o**). ^*^P < 0.05, ^**^P < 0.01 *vs*. control. CMC-Na carboxymethyl cellulose sodium, DEGs differentially expressed genes, GAPDH glyceraldehyde-3-phosphate dehydrogenase, GD gestational day, HFD high-fat diet, NCD normal chow diet, PDNL prednisolone, PPE prenatal prednisone exposure, PW postnatal week, WB western blot

To further investigate the potential common mechanisms by which PPE induces susceptibility to MASLD in male and female offspring, we conducted an in-depth analysis of mRNA-seq data from male and female fetal rat livers. Among all DEGs, 93 DEGs that were consistently altered in the livers of both male and female fetal rats in the PPE group were identified (Fig. 5a). Among these 93 common DEGs, the top 10 DEGs in PPE male fetal rat livers were *LOC103689983*, *Serpina7*, *Serpina3c*, *LOC100910990*, *Gata1*, *LOC108348106*, *novel.6041*, *Slc4a8*, *Cyp8b1* and *Abhd10* (Fig. 5b); the top 10 DEGs in females were *Serpina3c*, *LOC103689983*, *Cyp8b1*, *Ablim3*, *novel.6041*, *Serpina7*, *LOC100912538*, *Serpina3n*, *Mug2*, and *Slco1a1* (Fig. 5f). Notably, Serpina3c ranked among the three genes with the most significant changes in both sexes and presented the highest expression abundance in liver tissues (Fig. 5b, f). RT‒qPCR confirmed that hepatic *Serpina3c* mRNA expression was significantly lower in both male and female fetal rats in the PPE group than in those in the control group (Fig. 5c, g). WB analysis confirmed these changes at the protein level (Fig. 5d, e, h, i). At PW12, compared with that in the control group, hepatic *Serpina3c* expression remained significantly suppressed in both male and female offspring rats in the PPE group (Fig. 5j, m). Serpina3c is a member of the serine protease inhibitor (serpin) family and is a secreted protein.^56^ We further examined its concentration in serum. The results revealed that serum Serpina3c levels were also significantly reduced in the male and female offspring of the PPE group (Fig. 5k, n). Additionally, Pearson correlation analysis revealed a significant positive correlation between serum Serpina3c levels and hepatic *Serpina3c* mRNA expression in PPE male and female offspring rats (Fig. 5l, o). In vitro experiments demonstrated that prednisolone (the active metabolite of prednisone) significantly downregulated both the mRNA and protein expression of Serpina3c/SERPINA3 in the AML12 and HepG2 cell lines (Fig. 5p−r; Supplementary Fig. 8). Furthermore, analysis of sequencing data from MASLD patients and the healthy population revealed significantly reduced hepatic *SERPINA3* expression in MASLD patients (Supplementary Fig. 9). Collectively, these findings suggest that Serpina3c may serve as a potential toxicity target involved in mediating PPE-induced susceptibility to MASLD in offspring.

**Fig. 6:**
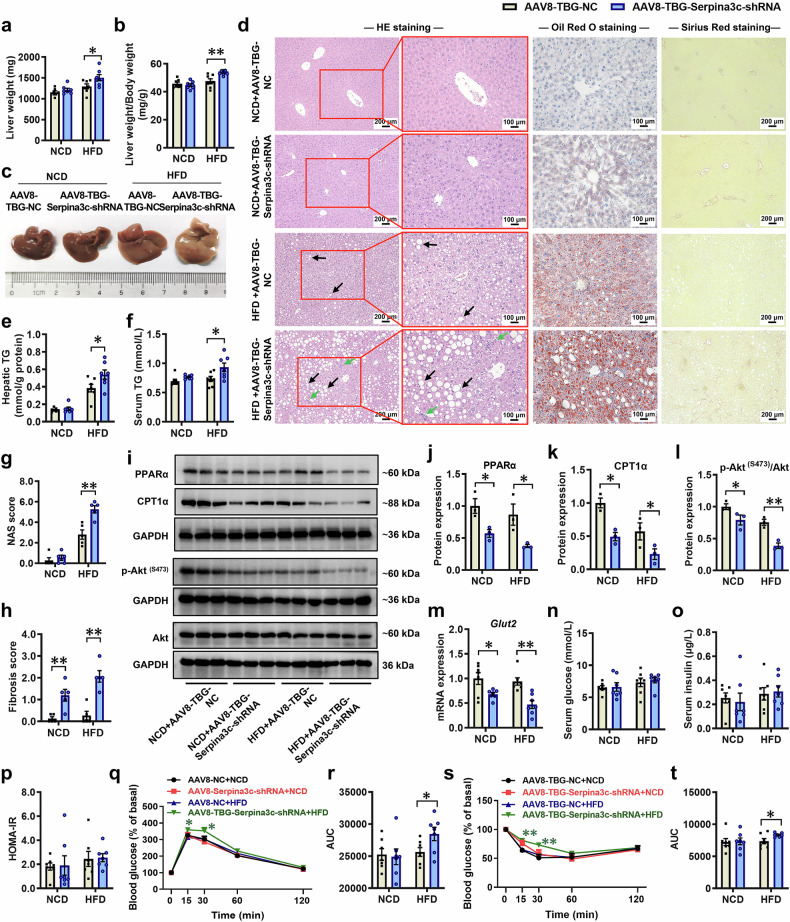
Liver-specific knockdown of Serpina3c increased susceptibility to MASLD in mice. Eight-week-old male C57BL/6 mice were administered AAV8-TBG-Serpina3c-shRNA *via* tail vein injection to knock down Serpina3c in the mouse liver, and samples were obtained at 12 weeks of age for further analysis. **a** Liver weights; **b** Liver weight/body weight; **c** Photograph of liver appearance; **d** Representative micrographs of liver sections stained with HE, oil red O and Sirius red, scale bar: 100 and 200 μm; **e** Hepatic TG content; **f** Serum TG levels; **g** Liver NAS score; **h** Liver fibrosis score; **i**−**l** Representative WB images and semiquantitative results of PPARα, CPT1α, Akt and p-Akt ^(S473)^ protein expression in the liver; **m** Hepatic *Glut2* mRNA expression; **n** Fasting serum glucose levels; **o** Fasting serum insulin levels; **p** HOMA-IR index; **q**, **r** Normalized blood glucose levels during the IPGTT and corresponding AUC; **s**, **t** Normalized blood glucose levels during the IPITT and corresponding AUC. Mean ± SEM, n = 3 for WB, n = 5 for liver histopathological data, n = 7 for other data. Statistical significance was determined by two-tailed unpaired Student’s *t* test (**a**−**p**, **r**, **t**) and two-way ANOVA for repeated measures followed by Bonferroni post hoc correction (**q**, **s**). *P < 0.05, **P < 0.01 *vs*. AAV8-TBG-NC. AAV8 adeno-associated virus serotype 8, Akt protein kinase B, AUC area under the curve, CPT1α carnitine palmitoyltransferase 1α, GAPDH glyceraldehyde-3-phosphate dehydrogenase, *Glut2* glucose transporter 2, HE hematoxylin and eosin, HFD high-fat diet, HOMA-IR homeostatic model assessment of insulin resistance, IPGTT intraperitoneal glucose tolerance test, IPITT intraperitoneal insulin tolerance test, NCD normal chow diet, p-Akt phospho-Akt, PPARα peroxisome proliferator-activated receptor α, TG triglyceride, TBG thyroxine-binding globulin, WB western blot

To verify whether Serpina3c is involved in regulating the occurrence of MASLD, we selected male C57BL/6 mice as study subjects (to avoid interference from the estrous cycle in females) and performed liver-specific Serpina3c knockdown *via* tail vein injection of AAV8-TBG-Serpina3c-shRNA (2.5 × 10^11 ^v.g.) (Supplementary Fig. 10a). Four weeks post-injection, a significant reduction in hepatic Serpina3c expression was confirmed in the AAV8-TBG-Serpina3c-shRNA group compared with the AAV8-TBG-NC group (Supplementary Fig. 10b, c). Under both NCD and HFD conditions, body weight changes in the mice in the AAV8-TBG-Serpina3c-shRNA group were not significantly different from those in the AAV8-TBG-NC group, and the body weight growth rate increased after 4 weeks of HFD feeding (Supplementary Fig. 10d, e). Compared with those in the AAV8-TBG-NC group, the liver weights and liver weight/body weight ratios of the mice in the AAV8-TBG-Serpina3c-shRNA group markedly increased under HFD conditions, whereas these changes were not observed under NCD conditions (Fig. 6a, b). In addition, compared with those in the AAV8-TBG-NC group, the livers of the mice in the AAV8-TBG-Serpina3c-shRNA group fed a HFD were more yellow in color (Fig. 6c). Moreover, liver histopathology revealed more severe hepatic steatosis, lipid accumulation, inflammatory infiltration, and collagen fiber deposition in the AAV8-TBG-Serpina3c-shRNA group (Fig. 6d). Significant increases in hepatic TG content, serum TG levels, and liver pathological scores were also observed in the AAV8-TBG-Serpina3c-shRNA group (Fig. 6e−h). RT‒qPCR analysis revealed significantly lower expression of key fatty acid oxidation genes (*Ppara*, *Cpt1a*, *Ehhadh*) in the AAV8-TBG-Serpina3c-shRNA group than in the AAV8-TBG-NC group under both NCD and HFD conditions (Supplementary Fig. 10f). The WB results also revealed that the expression levels of key proteins related to fatty acid oxidation (PPARα and CPT1α) in the livers of the mice in the AAV8-TBG-Serpina3c-shRNA group were significantly lower than those in the AAV8-TBG-NC group under both NCD and HFD conditions (Fig. 6i−k). Moreover, the expression of key genes and proteins involved in the insulin signaling pathway and glucose transporters was also significantly reduced in the livers of the mice in the AAV8-TBG-Serpina3c-shRNA group (Fig. 6i, l, m). Although significant alterations in fasting serum glucose and insulin levels and homeostasis model assessment of insulin resistance (HOMA-IR) indices were not observed (Fig. 6n−p), glucose tolerance and insulin sensitivity were significantly reduced in the AAV8-TBG-Serpina3c-shRNA group under HFD conditions (Fig. 6q−t). These results demonstrate that low expression of Serpina3c disrupts hepatic glucose and lipid metabolism, thereby increasing susceptibility to MASLD.

### Serpina3c regulates hepatic glucose and lipid metabolism in PPE offspring through the chymase-Ang Ⅱ-AT1R pathway

**Fig. 7:**
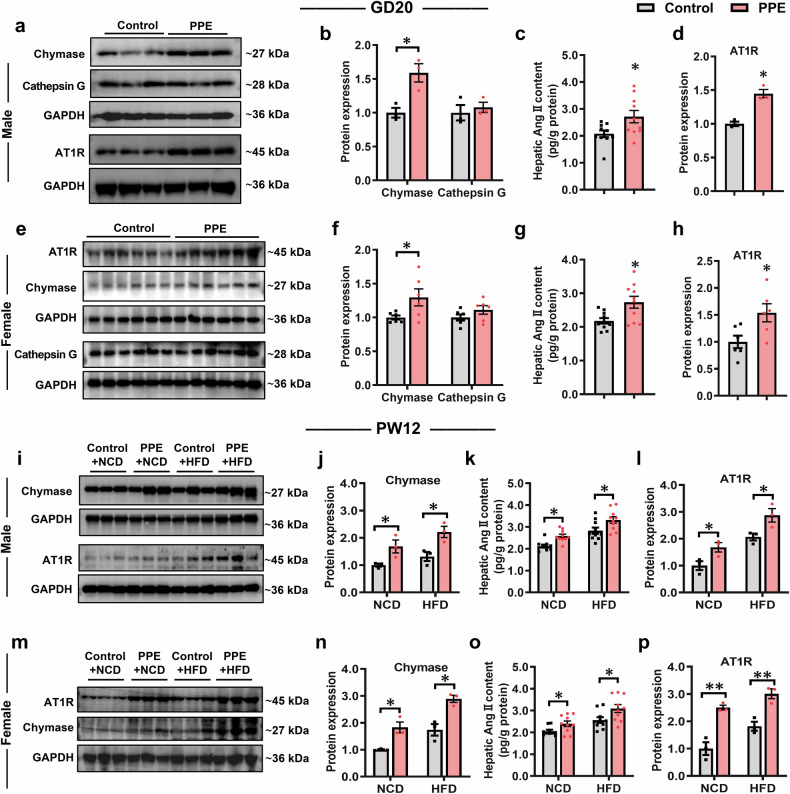
PPE activated the chymase-Ang II-AT1R pathway in male and female offspring rats. Pregnant Wistar rats were intragastrically administered prednisone (0.25 mg/kg) or vehicle control (0.5% CMC-Na) per day from GD0−20, and offspring rats of different ages (GD20 and PW12) were subsequently obtained for further analysis. Among these offspring, some were fed a HFD (D12492) from PW8−12. **a**, **b**, **d**, **e**, **f**, **h** Representative WB images and semiquantitative results of hepatic chymase, cathepsin G and AT1R protein expression in both male and female fetal rats on GD20; **c**, **g** Hepatic Ang II content in both male and female fetal rats on GD20; **i**, **j**, **l**, **m**, **n**, **p** Representative WB images and semiquantitative results of hepatic chymase and AT1R protein expression in both male and female offspring rats on PW12; k, **o** Hepatic Ang II content in both male and female offspring rats on PW12. Mean ± SEM, n = 3 or 6 for the WB data, n = 10 for the hepatic Ang II content data. Statistical significance was determined by two-tailed unpaired Student’s *t* test. ^*^P < 0.05 *vs*. control. Ang II angiotensin II, AT1R angiotensin Ⅱ type 1 receptor, CMC-Na carboxymethyl cellulose sodium, GAPDH glyceraldehyde-3-phosphate dehydrogenase, GD gestational day, HFD high-fat diet, NCD normal chow diet, PPE prenatal prednisone exposure, PW postnatal week, WB western blot

We subsequently focused on the involvement of low Serpina3c expression in mediating the regulatory mechanism of MASLD susceptibility in PPE male and female offspring. As a serine protease inhibitor, Serpina3c exerts its biological functions by targeting and inactivating serine proteases such as chymase and cathepsin G.^57,58^ The results revealed that hepatic chymase protein expression levels in male and female fetal rats were significantly elevated in the PPE group compared with those in the control group, whereas no significant changes in cathepsin G protein expression levels were detected (Fig. 7a, b, e, f). At PW12, the PPE male and female offspring rats presented markedly increased hepatic chymase expression under both NCD and HFD conditions compared with the control group (Fig. 7i, j, m, n). Chymase has been reported to be responsible for ACE-independent angiotensin II (Ang II) formation in multiple tissues.^59–61^ We further assessed hepatic Ang II content and the expression of its receptor AT1R. The results revealed that hepatic Ang II content was significantly increased in PPE male and female offspring rats before and after birth (Fig. 7c, g, k, o), and AT1R protein expression was also significantly increased (Fig. 7a, d, e, h, i, l, m, p). These findings indicated sustained activation of the chymase-Ang II-AT1R pathway in the livers of PPE male and female offspring rats before and after birth. To determine whether this activation results from Serpina3c downregulation, we further examined the chymase-Ang II-AT1R pathway in liver-specific Serpina3c-knockdown mice. Compared with that in the AAV8-TBG-NC group, hepatic chymase protein expression was significantly increased in the AAV8-TBG-Serpina3c-shRNA group (Supplementary Fig. 10g, h). Moreover, the hepatic Ang II content and AT1R expression were significantly increased (Supplementary Fig. 10i−k). In vitro, the knockdown of Serpina3c in AML12 cells resulted in a significant increase in the intracellular Ang II content and AT1R expression, which was more pronounced after stimulation with chymase (100 ng/mL) (Supplementary Fig. 11a−f); this increase was accompanied by a decrease in fatty acid oxidation and glucose uptake function, as well as an increase in intracellular lipid accumulation (Supplementary Figs. 11g−l). Consistent with the results observed after the knockdown of Serpina3, similar results were observed in prednisolone-treated AML12 cells after chymase stimulation (Supplementary Fig. 12). Critically, prednisolone-treated cells overexpressing Serpina3c were significantly resistant to the above changes induced by chymase stimulation (Supplementary Fig. 12). These results suggested that Serpina3c deficiency activated the chymase-Ang II-AT1R pathway, thereby driving hepatic glucose and lipid dysregulation and enhancing susceptibility to MASLD in PPE male and female offspring.

### Glucocorticoid receptor (GR)-histone deacetylase3 (HDAC3) signaling activation mediates the reduction in histone H3 lysine 27 acetylation (H3K27ac) levels at the Serpina3c promoter region and its expression in hepatocytes induced by PPE

**Fig. 8:**
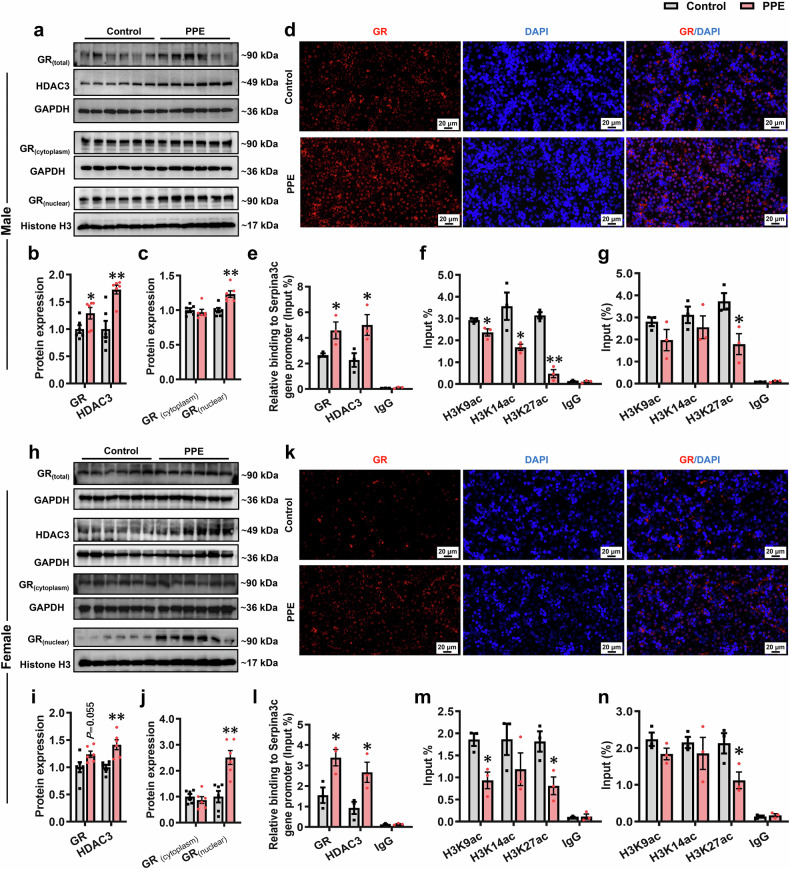
PPE activated GR-HDAC3 signaling and reduced histone acetylation levels of the *Serpina3c* promoter region in vivo. Pregnant Wistar rats were intragastrically administered prednisone (0.25 mg/kg) or vehicle control (0.5% CMC-Na) per day from GD0−20, and offspring rats of different ages (GD20 and PW12) were subsequently obtained for further analysis. (**a**−**c**, **h**−**j**) Representative WB images and semiquantitative results of hepatic GR and HDAC3 protein expression in both male and female fetal rats on GD20; **d**, **k** Representative immunofluorescence images of GR in the livers of male and female fetal rats on GD20, scale bar: 20 μm); **e**, **l** Enrichment of GR and HDAC3 in the *Serpina3c* gene promoter region in the livers of male and female fetal rats on GD20; **f**, **m** H3K9ac, H3K14ac, and H3K27ac in the *Serpina3c* promoter region in the livers of male and female fetal rats on GD20; **g**, **n** H3K9ac, H3K14ac, and H3K27ac in the *Serpina3c* promoter region in the livers of male and female offspring rats on PW12. Mean ± SEM, n = 6 for WB; n = 3 for other data. Statistical significance was determined by two-tailed unpaired Student’s *t* test (or two-tailed unpaired Student’s *t* test with Welch’s correction). ^*^P < 0.05, ^**^P < 0.01 *vs*. control. GAPDH glyceraldehyde-3-phosphate dehydrogenase, GD gestational day, GR glucocorticoid receptor, H3K9ac histone H3 lysine 9 acetylation, H3K14ac histone H3 lysine 14 acetylation, H3K27ac histone H3 lysine 27 acetylation, HDAC3 histone deacetylase 3, PPE prenatal prednisone exposure, WB western blot

**Fig. 9:**
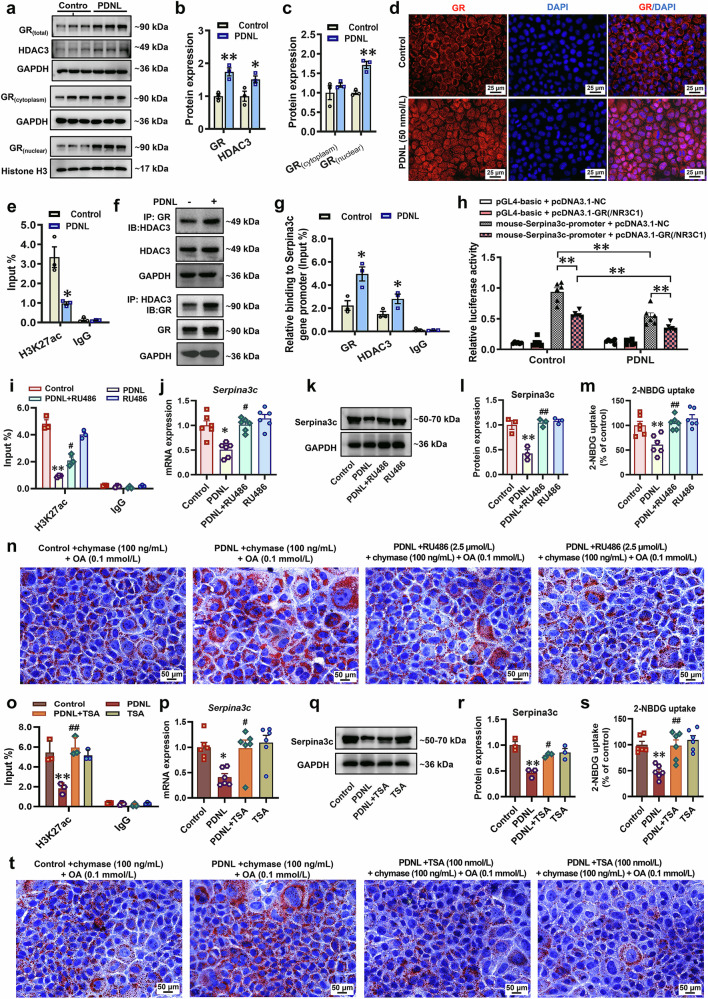
Prednisolone activated GR-HDAC3 signaling and reduced histone acetylation levels of the *Serpina3c* promoter region in AML12 cells in vitro. AML12 cells were cultured with or without TSA (100 nmol/L) or RU486 (2.5 μmol/L) in the presence of prednisolone (0 or 50 nmol/L) for 72 h. **a**−**c** Representative WB images and semiquantitative results of GR and HDAC3. **d** Representative immunofluorescence images of GR; scale bar: 25 μm. **e**, **i**, **o** H3K27ac levels in the *Serpina3c* promoter region. **f** Protein binding between GR and HDAC3. **g** Enrichment of GR and HDAC3 in the *Serpina3c* promoter region. **h** Dual-luciferase reporter gene assay validating the GR regulation of *Serpina3c*. **j**, **p** *Serpina3c* mRNA expression. **k**, **l**, **q**, **r** Representative WB images and semiquantitative results of Serpina3c protein expression; **m**, **s** 2-NBDG uptake; **n**, **t** Representative micrographs of oil red O staining, scale bar: 50 μm. Mean ± SEM, n = 6 for RT‒qPCR and dual luciferase reporter gene assays; n = 3 for other data. Statistical significance was determined by two-tailed unpaired Student’s *t* test (**a**−**g**) and one-way ANOVA with Tukey’s post hoc test (h−t). ^*^P < 0.05, ^**^P < 0.01 *vs*. the control; ^#^*P* < 0.05, ^##^*P* < 0.01 *vs*. the prednisolone-treated group. DAPI, 4’,6-diamidino-2-phenylindole; GAPDH glyceraldehyde-3-phosphate dehydrogenase, GR glucocorticoid receptor, H3K27ac histone H3 lysine 27 acetylation, HDAC3 histone deacetylase 3, PDNL prednisolone, RT‒qPCR reverse-transcription quantitative real-time PCR, RU486 mifepristone, TSA trichostatin A, WB western blot

We further investigated the potential common programming mechanisms underlying the PPE-induced low expression of hepatic Serpina3c in both male and female offspring. We examined DNA methylation levels in the *Serpina3c* promoter region via next-generation sequencing-based bisulfite sequencing PCR (BSP). The results indicated that PPE did not alter DNA methylation levels in the *Serpina3c* promoter region in either male or female fetal rat liver tissues (Supplementary Fig. 13). This finding indicated that DNA methylation was not involved in regulating the low expression of serpina3c in the livers of PPE male and female offspring. Subsequently, predictive analyses via both the JASPAR database (https://jaspar.elixir.no/) and the AnimalTFDB v4.0 database (http://bioinfo.life.hust.edu.cn/AnimalTFDB4/) revealed the presence of GR (/*NR3C1*) binding sites in the *Serpina3c* promoter region (Supplementary Fig. 14a−c). Concurrently, the Cistrome Data Browser (http://cistrome.org/db/#/) demonstrated that the *Serpina3c* promoter region contains not only GR binding sites but also binding sites for the epigenetic modifier HDAC3 (Supplementary Fig. 14c). Moreover, the most common form of epigenetic modification of *Serpina3c* was H3K27ac (Supplementary Fig. 14d). Compared with those in the control group, male fetal livers in the PPE group presented significantly increased total GR protein expression and increased nuclear translocation (Fig. 8a−d). Among the common HDACs, HDAC3 gene expression levels in PPE male fetal rat livers were significantly elevated (Supplementary Fig. 15), and its protein expression levels were significantly increased (Fig. 8a, b). ChIP‒qPCR analysis confirmed enhanced enrichment of both GR and HDAC3 at the *Serpina3c* promoter in the PPE male fetal rat liver (Fig. 8e). We screened the histone acetylation levels at common sites (H3K9, H3K14, and H3K27) in the *Serpina3c* promoter region. The ChIP‒qPCR results revealed significantly reduced H3K27ac levels at the *Serpina3c* promoter in the livers of PPE male offspring before and after birth (Fig. 8f, g). Similarly, consistent results were also observed in the livers of PPE female offspring rats (Fig. 8h−n). Consistent with the in vivo findings, total and nuclear GR protein levels as well as HDAC3 protein expression were significantly increased in AML12 and HepG2 cells after 72 h of prednisolone (50 nmol/L) treatment (Fig. 9a−d; Supplementary Fig. 16a−c). The levels of H3K27ac in the *Serpina3c/SERPINA3* promoter region were also significantly reduced after prednisolone treatment (Fig. 9e; Supplementary Fig. 16d). Co-immunoprecipitation (Co-IP) assays also confirmed that GR binds to HDAC3 proteins (Fig. 9f), and their enrichment in the *Serpina3c* promoter region was significantly increased after prednisolone treatment (Fig. 9g). Dual-luciferase reporter assays in AML12 cells further validated the regulatory effects of GR on Serpina3c (Fig. 9h). These results demonstrated that PPE (/prednisolone) activates GR-HDAC3 signaling both in vivo and in vitro, leading to reduced H3K27ac levels and consequent downregulation of Serpina3c expression.

To verify the above speculations, we treated AML12 cells with the GR antagonist mifepristone (RU486, 2.5 μmol/L) and the deacetylase inhibitor tricostatin A (TSA, 100 nmol/L). The results revealed that RU486 or TSA treatment significantly restored H3K27ac levels in the *Serpina3c* promoter region and that H3K27ac expression was induced by prednisolone, and there was a significant improvement in hepatocyte lipid accumulation and glucose uptake (Fig. 9i−t). Moreover, we knocked down GR and HDAC3 expression in HepG2 cells via GR siRNA and HDAC3 siRNA, respectively (Supplementary Fig. 16e, f). The results also revealed that the prednisolone-induced reduction in SERPINA3 expression could be reversed by GR or HDAC3 knockdown (Supplementary Fig. 16g, h). The above results suggested that GR-HDAC3 signaling mediated the prednisolone-induced reduction in Serpina3c/SERPINA3 expression.

### Postnatal overexpression of hepatic Serpina3c alleviates susceptibility to MASLD in PPE offspring

**Fig. 10:**
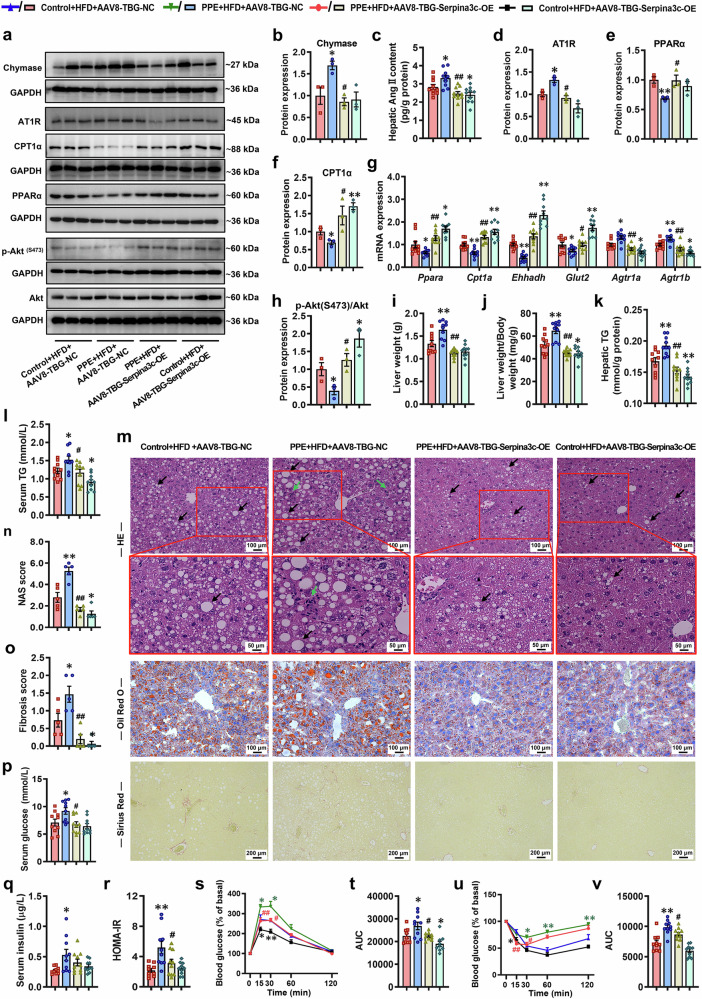
Liver-specific overexpression of Serpina3c improved the chymase-Ang II-AT1R pathway and susceptibility to MASLD in PPE male offspring mice. Pregnant C57BL/6 mice were intragastrically administered vehicle control (0.5% CMC-Na) or prednisone (0.5 mg/kg) per day from GD0−18, and then, male offspring mice at PW8 were fed a HFD and administered AAV8-TBG-Serpina3c-OE (2.5 × 10 ^11^ v.g.) *via* tail vein injection to overexpress Serpina3c in the liver. Samples were obtained at PW12 for further analysis. **a**, **b**, **d**−**f**, **h** Representative WB images and semiquantitative results of PPARα, CPT1α, Akt, p-Akt ^(S473)^, chymase and AT1R protein expression in the liver; **c** hepatic Ang II content; **g** mRNA expression of glucose and lipid metabolism-related genes (*Ppara*, *Cpt1a*, *Ehhadh* and *Glut2*) and AT1R coding genes (*Agtr1a* and *Agtr1b*) in the liver; **i** liver weights; **j** liver weight/body weights; **k** hepatic TG content; **l** serum TG levels; **m** representative micrographs of liver sections stained with HE, oil red O and Sirius red staining, scale bar: 50, 100 and 200 μm; **n** liver NAS score; **o** liver fibrosis score; **p** fasting serum glucose levels; **q** fasting serum insulin levels; **r** HOMA-IR index; **s**, **t** normalized blood glucose levels during the IPGTT and corresponding AUC; **u**, **v** normalized blood glucose levels during the IPITT and corresponding AUC. Mean ± SEM, n = 3 for WB, n = 5 for liver histopathological data, n = 10 for other data. Statistical significance was determined by one-way ANOVA with Tukey’s post hoc test (**a**−**r**, **t**, **v**) and two-way ANOVA for repeated measures followed by Bonferroni post hoc correction (**s**, **u**). ^*^P < 0.05, ^**^P < 0.01 *vs*. control + HFD + AAV8-TBG-NC; ^#^*P* < 0.05, ^##^*P* < 0.01 *vs*. PPE + HFD + AAV8-TBG-NC. AAV8 adeno-associated virus serotype 8, Akt protein kinase B, Ang II angiotensin II, AT1R angiotensin II type 1 receptor, AUC area under the curve, CPT1α (/*Cpt1a*) carnitine palmitoyltransferase 1α, *Ehhadh* enoyl-CoA hydratase and 3-hydroxyacyl CoA dehydrogenase, GAPDH glyceraldehyde-3-phosphate dehydrogenase, *Glut2* glucose transporter 2, HE hematoxylin and eosin, HFD high-fat diet, HOMA-IR homeostatic model assessment of insulin resistance, IPGTT intraperitoneal glucose tolerance test, IPITT intraperitoneal insulin tolerance test, OE overexpression, p-Akt phospho-Akt, PPARα(/*Ppara*) peroxisome proliferator-activated receptor α, PPE prenatal prednisone exposure, TBG thyroxine-binding globulin, TG triglyceride, WB western blot

**Fig. 11:**
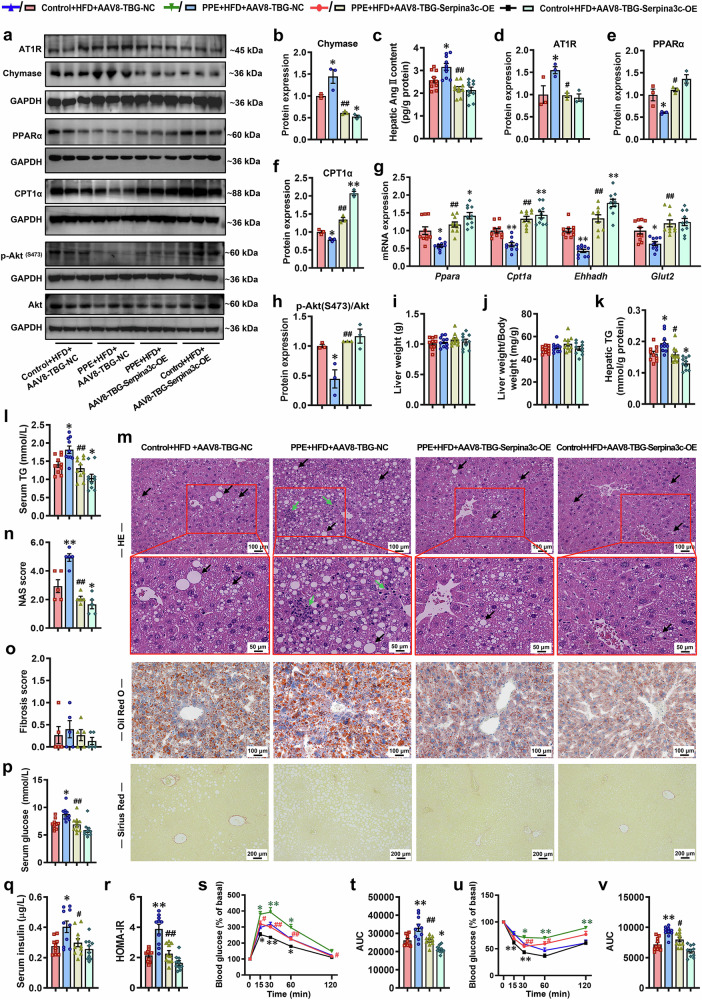
Liver-specific overexpression of Serpina3c improved the chymase-Ang II-AT1R pathway and susceptibility to MASLD in PPE female offspring mice. Pregnant C57BL/6 mice were intragastrically administered vehicle control (0.5% CMC-Na) or prednisone (0.5 mg/kg) per day from GD0−18, and then, female offspring mice at PW8 were fed an HFD and administered AAV8-TBG-Serpina3c-OE (2.5 × 10 ^11^ v.g.) *via* tail vein injection to overexpress Serpina3c in the liver. Samples were obtained at PW12 for further analysis. **a**, **b**, **d**−**f**, **h** Representative WB images and semiquantitative results of PPARα, CPT1α, Akt, p-Akt ^(S473)^, chymase and AT1R protein expression in the liver; **c** hepatic Ang II content; **g** mRNA expression of glucose and lipid metabolism-related genes (*Ppara*, *Cpt1a*, *Ehhadh* and *Glut2*) in the liver; **i** liver weights; **j** liver weight/body weights; **k** hepatic TG content; **l** serum TG levels; **m** representative micrographs of liver sections stained with HE, oil red O and Sirius red staining, scale bar: 200 and 100 μm; **n** liver NAS score; **o** liver fibrosis score; **p** fasting serum glucose levels; **q** fasting serum insulin levels; **r** HOMA-IR index; **s**, **t** normalized blood glucose levels during the IPGTT and corresponding AUC; **u**, **v** normalized blood glucose levels during the IPITT and corresponding AUC. Mean ± SEM, n = 3 for WB, n = 5 for liver histopathological data, n = 10 for other data. Statistical significance was determined by one-way ANOVA with Tukey’s post hoc test (**a**−**r**, **t**, **v**) and two-way ANOVA for repeated measures followed by Bonferroni post hoc correction (**s**, **u**). ^*^P < 0.05, ^**^P < 0.01 *vs*. control + HFD + AAV8-TBG-NC; ^#^*P* < 0.05, ^##^*P* < 0.01 *vs*. PPE + HFD + AAV8-TBG-NC. AAV8, adeno-associated virus serotype 8; Akt protein kinase B, Ang II angiotensin II, AT1R angiotensin II type 1 receptor, AUC area under the curve, CPT1α (/*Cpt1a*) carnitine palmitoyltransferase 1α, *Ehhadh* enoyl-CoA hydratase and 3-hydroxyacyl CoA dehydrogenase, GAPDH glyceraldehyde-3-phosphate dehydrogenase, *Glut2* glucose transporter 2, HE hematoxylin and eosin, HFD high-fat diet, HOMA-IR homeostatic model assessment of insulin resistance, IPGTT intraperitoneal glucose tolerance test, IPITT intraperitoneal insulin tolerance test, OE overexpression, p-Akt phospho-Akt, PPARα(/*Ppara*) peroxisome proliferator-activated receptor α, PPE prenatal prednisone exposure, TBG thyroxine-binding globulin, TG triglyceride, WB western blot

To further determine the feasibility of Serpina3c as a potential target for intervention in postnatal offspring, we injected AAV8-TBG-Serpina3c-OE (2.5 × 10^11^ v.g.) into PPE male and female offspring *via* the tail vein at PW8 for liver-specific Serpina3c overexpression. In male offspring mice fed an HFD, liver-specific Serpina3c overexpression significantly reversed the increase in hepatic chymase protein expression, Ang II content, and AT1R expression induced by PPE (Fig. 10a−d, g). The RT‒qPCR results revealed that hepatic Serpina3c overexpression reversed the altered expression of key genes related to fatty acid oxidation and glucose uptake (*Pparα*, *Cpt1α*, *Ehhadh*, and *Glut2*) in the livers of PPE male offspring mice fed a HFD (Fig. 10g). The WB results also revealed that the reduced expression of key proteins related to fatty acid oxidation and insulin signaling pathways in the livers of PPE male offspring mice fed a HFD was significantly reversed by hepatic Serpina3c overexpression (Fig. 10a, e, f, h). Furthermore, Serpina3c overexpression significantly reduced the liver weight, liver weight/body weight ratio, hepatic TG content, serum TG level, liver NAS and fibrosis score and significantly alleviated hepatic pathological alterations (e.g., hepatic steatosis, lipid accumulation, inflammatory infiltration, and collagen fiber deposition) in PPE male offspring mice fed a HFD (Fig. 10i−o). Moreover, Serpina3c overexpression also significantly improved glucose metabolic homeostasis in PPE male offspring mice fed a HFD, as evidenced by reduced fasting glucose levels and HOMA-IR indices and increased glucose tolerance and insulin sensitivity (Fig. 10p−v). Similarly, the ameliorative effect of liver-specific Serpina3c overexpression was also observed in PPE female offspring mice fed a HFD (Fig. 11). These results suggested that liver-specific Serpina3c overexpression reversed the activation of the hepatic chymase-Ang II-AT1R pathway, which in turn markedly alleviated susceptibility to MASLD in PPE offspring.

## Discussion

In this study, we evaluated the short- and long-term effects of PPE on hepatic glucose and lipid metabolism functions, as well as the susceptibility of offspring to MASLD. The main findings are as follows: ① PPE disrupted hepatic glucose and lipid metabolism in offspring before and after birth, increasing their susceptibility to MASLD, particularly under HFD challenge; ② Serpina3c was identified as a key regulator of hepatic metabolism, and its persistent low expression could cause the activation of the chymase-Ang Ⅱ-AT1R pathway, which could result in the dysfunction of hepatic glucose and lipid metabolism and increase susceptibility to MASLD in PPE offspring; ③ the sustained suppression of Serpina3c was associated with a reduction in H3K27ac levels in the promoter region of Serpina3c caused by the activation of GR-HDAC3 signaling; ④ hepatic Serpina3c could be a potential therapeutic target, and postnatal restoration of its expression can alleviate susceptibility to MASLD in PPE offspring. This study is highly practical for analyzing the effects of PPE on long-term metabolic health in offspring and guiding the prevention and treatment of fetal-originated MASLD.

Clinically, low-dose prednisone (2.5–10 mg/d) is administered orally throughout pregnancy to control maternal disease activity and minimize potential adverse effects.^62–65^ Assuming that the average body weight of pregnant women during pregnancy is approximately 60 kg,^66–68^ the daily dose of prednisone for pregnant women is 0.042–0.1667 mg/(kg·d), which corresponds to an equivalent dose of 0.259–1.028 mg/(kg·d) in rats or 0.518–2.056 mg/(kg·d) in mice according to the dose conversion equation between humans and animals.^69,70^ In addition, the gestation period is approximately 20–23 days for rats and 18–21days for mice. To conform to the clinical dosage, pregnant rats in this study were given 0.25 mg/(kg·d) prednisone *via* oral gavage throughout gestation (GD0–20), whereas pregnant mice were given 0.5 mg/(kg·d) prednisone *via* oral gavage throughout gestation (GD0–18). Therefore, the animal (rat and mouse) model of PPE constructed in this study has important theoretical value and clinical relevance for exploring the short- and long-term risks of prednisone-induced liver developmental and functional toxicity.

Fetal structural and physiologic changes caused by adverse environmental exposures early in life increase susceptibility to multiple diseases later in life. During fetal development, a “first hit” can cause morphological changes and functional adaptations in critical organs, but this alone may not be sufficient to cause phenotypic changes in disease. Subsequent exposure to another type of insult postnatally may act as a “second hit”, during which latent adverse modifications could become apparent or amplified, ultimately leading to disease occurrence. Consequently, the “two-hit” model has been widely adopted in animal studies for investigating the developmental origins of disease.^71,72^ In the current research on the developmental origins of MASLD, the potential “first hit” may encompass various forms, including maternal diseases, overnutrition/undernutrition, and exposure to toxins/pharmaceuticals during pregnancy.^43,73^ A HFD is commonly used as the “second hit” approach, as exemplified by models such as “maternal obstructive sleep apnea + offspring fed with HFD”,^74^ “perinatal bisphenol A exposure + offspring fed with HFD”,^75^ and “maternal sucralose consumption + offspring fed with HFD”.^76^ Therefore, in the present study, we employed a HFD as the “second hit” to establish the MASLD model; specifically, PPE offspring were fed a HFD from PW8 to PW12. In this study, our results demonstrated that both male and female PPE offspring developed hepatic glucose and lipid metabolic disorders, with more pronounced MASLD alterations under HFD challenge. We even observed significant fibrosis progression in the livers of male PPE offspring rats. These findings demonstrate that PPE significantly increases susceptibility to MASLD in offspring, supporting the concept that PPE serves as a “first hit” in the developmental programming of MASLD.

Serpina3c is an extracellular secretory protein belonging to the serpin family, branch “A”, whose human homolog is SerpinA3. It has been implicated in diverse physiological and pathological processes.^57,77,78^ The specificity and function of serpins are determined by their reactive center loop, which extends from the main body of the peptide and guides the binding of the inhibitor to target proteases. Previous studies have shown that Serpina3c exerts a protective effect on the development of atherosclerosis.^79^ Additionally, Choi et al.^80^ reported that Serpina3c is a key regulator of adipogenesis, and its inhibition may have therapeutic benefits for obesity. Under certain pathophysiological conditions, downregulation of Serpina3c expression in various tissues leads to more severe metabolic disorders in mice.^57,77,81^ These findings suggest an association between Serpina3c and metabolic diseases. In this study, mRNA-seq screening revealed that Serpina3c was among the top 3 DEGs in the livers of both male and female PPE fetal rats, with the highest expression abundance in the liver. Further validation confirmed that Serpina3c expression was significantly reduced in the livers of PPE offspring before and after birth in both sexes. Moreover, a marked suppression of *SERPINA3* expression was observed in the livers of MASLD patients. We further observed dysfunctional hepatic glucose and lipid metabolism in liver-specific Serpina3c-knockdown mice and more pronounced MASLD alterations under HFD conditions. These findings indicate that low hepatic Serpina3c expression leads to dysregulated glucose and lipid metabolism, thereby mediating increased susceptibility to MASLD in PPE offspring. Moreover, Serpina3c is a secreted protein. We further discovered through correlation analysis that alterations in serum Serpina3 levels are significantly correlated with changes in hepatic Serpina3 expression. This correlation suggests that circulating Serpina3c levels could serve as potential biomarkers for early prediction of susceptibility to MASLD in offspring.

Serpina3c acts as a serine protease inhibitor that exerts relevant effects under physiological and pathological conditions by targeting and inactivating multiple serine proteases, such as cathepsin G and chymase.^57,58^ Cathepsin G, a member of the neutrophil serine protease family, is renowned for its pathogen-killing properties and serves as a critical modulator of immune responses and inflammation.^82^ Chymase is predominantly recognized as a chymotrypsin-like enzyme produced by mast cells and plays a pivotal role in Ang II formation across diverse tissues.^59–61^ It has been reported that chymase activity is significantly elevated in cirrhotic patients, with a strong correlation observed between chymase levels and fibrosis scores.^83^ Our results revealed that PPE markedly increased hepatic chymase expression in offspring before and after birth, and similar alterations were observed in the livers of liver-specific Serpina3c-knockdown mice. These data suggest that chymase may be involved in mediating the regulatory effect of Serpina3c on susceptibility to MASLD in PPE offspring. Chymase is responsible for ACE-independent Ang II generation in tissues.^59–61^ Ang II has been reported to increase reactive oxygen species levels by binding to its receptor AT1R, thereby inducing insulin resistance, hepatic steatosis, and inflammation.^84,85^ Additionally, Ang II promoted liver fibrosis by inducing α-smooth muscle actin in hepatic stellate cells.^86^ In this study, we further observed that hepatic Ang II content and AT1R expression were consistently elevated in PPE offspring and in Serpina3c-silenced mice, consistent with chymase upregulation. In vitro, hepatocytes with Serpina3c knockdown exhibited reduced resistance to chymase-induced activation of the Ang II-AT1R pathway and subsequent glucose and lipid metabolic disturbances, similar to the effects of prednisolone treatment. Conversely, Serpina3c overexpression in prednisolone-treated cells attenuated chymase-triggered Ang II-AT1R pathway activation and metabolic dysregulation. In summary, low Serpina3c expression might activate the chymase-Ang II-AT1R pathway, thereby mediating susceptibility to MASLD in PPE offspring.

In this study, we identified 93 DEGs that were consistently altered in the livers of both male and female fetal rats in the PPE group. Among these, *LOC103689983, Serpina7, Cyp8b1* and novel.6041—in addition to Serpina3c—were also among the top 10 DEGs in both sexes. On the basis of genomic annotation, *LOC103689983* corresponds to *Hprt1* (hypoxanthine phosphoribosyltransferase 1; Gene ID: 24465), which encodes hypoxanthine-guanine phosphoribosyl transferase, a key enzyme involved in purine metabolism.^87^ *Serpina7* encodes thyroxine-binding globulin, a major serum transport protein for thyroid hormones.^88^ *Cyp8b1* encodes a cytochrome P450 enzyme that catalyzes the side-chain oxidation cleavage of cholesterol within the classical bile acid biosynthetic pathway.^89^ novel.6041 represents the prediction of a novel gene due to the absence of annotation information in the reference genome. Whether these genes also contribute to MASLD susceptibility in PPE offspring warrants further investigation.

Under glucocorticoid action, GR can induce long-term gene expression changes through epigenetic modifications, leading to persistent alterations in organ structure and function.^90–92^ Several animal studies have demonstrated the direct effects of glucocorticoids on fetal tissues by using tissue-specific GR knockout models.^93,94^ Both endogenous and synthetic glucocorticoids alter epigenetic modifications during fetal development, causing gene expression changes that persist from the intrauterine stage into the postnatal stage.^92,95^ Bioinformatic analysis predicted potential GR and HDAC3 binding sites in the *Serpina3c* promoter region. In vivo, PPE increased hepatic GR and HDAC3 expression and enhanced GR nuclear translocation in fetuses. Concurrently, we observed increased GR and HDAC3 enrichment at the Serpina3c promoter region, accompanied by decreased H3K27ac levels. Similar results were obtained in vitro using both mouse AML12 cells and human HepG2 cells. Co-IP experiments also confirmed the interaction between GR and HDAC3. Furthermore, the inhibition of either GR or HDAC3 reversed the above changes. These findings indicate that PPE activates GR through prednisolone, which subsequently upregulates and recruits HDAC3 to form a GR-HDAC3 complex. This further led to a decrease in the level of H3K27ac at the Serpina3c promoter region, which subsequently caused sustained low expression of Serpina3c.

HDAC3, a class I histone deacetylase, is a core component of the NCoR/SMRT corepressor complex and plays crucial roles in gene regulation.^96,97^ It has been shown that HDAC3 mainly deacetylates modifications on the histone H3K27 site.^98–102^ Consistently, our current study confirmed the deacetylation of the H3K27 site in the promoter region of Serpina3c by HDAC3. HDAC3 has been reported to be primarily responsible for transcriptional repression mediated by nuclear receptors and other transcription factors.^96,97^ It was also shown that HDAC3 is involved in mediating the repression of GR on its target genes after dexamethasone exposure.^103,104^ These collective findings suggest that GR-HDAC3 signaling may play an important role in the glucocorticoid-mediated repression of downstream target genes *via* GR.

Fetal-originated diseases present significant challenges for early prevention and treatment because of their unclear etiology, undefined pathogenic mechanisms, and lack of intervention targets.^32^ Currently, “fetal developmental programming” has become one of the most important theories for understanding healthy developmental programming across the entire life course. Many studies have suggested that adverse environments during development increase the risk of chronic diseases.^105,106^ Although many researchers have elucidated the underlying pathophysiology of the intrauterine origin of adult disease, the ultimate goal is to develop primary prevention strategies and/or therapeutic interventions for individuals at increased risk. Prednisone, as an immunosuppressant, is often unavoidably used during pregnancy due to maternal illness or other reasons.^107^ In particular, approximately 55% of pregnant women with rheumatoid arthritis reportedly use prednisone.^6^ However, in this study, we demonstrated that PPE disrupts hepatic glucose and lipid metabolism in offspring, increasing susceptibility to MASLD. Thus, exploring intervention strategies to prevent the occurrence of MASLD in PPE offspring is critically important. Currently, personalized interventions have been proposed to mitigate increased disease susceptibility caused by fetal programming in individuals who have already experienced the “first hit”. For example, postnatal lentiviral delivery of ACE siRNA reversed impaired peak bone mass accumulation in offspring induced by dexamethasone exposure during pregnancy.^108^ Our recent studies also demonstrated that postnatal circGtdc1 or PPARGC1A overexpression ameliorated cartilage quality deterioration and susceptibility to osteoarthritis in offspring induced by PPE.^23,55^ Previous studies have confirmed that Serpina3c significantly improves HFD-induced atherosclerosis and pancreatic dysfunction.^77,79^ In this study, we screened and identified Serpina3c as a key target in MASLD pathogenesis induced by PPE. We further confirmed that low expression of hepatic Serpina3c increased susceptibility to MASLD, while hepatic Serpina3c overexpression markedly reversed the activation of the chymase-Ang II-AT1R pathway, improved hepatic glucose and lipid metabolism, and ultimately reduced susceptibility to MASLD in PPE offspring. Therefore, Serpina3c represents a promising therapeutic target for mitigating MASLD susceptibility in PPE offspring, highlighting the feasibility of the development of drug therapies that target Serpina3c.

Sexual dimorphism in terms of prevalence, age of onset, disease course or severity is common in many fetal-originated diseases.^109–111^ In this study, while we focused primarily on the common alterations in hepatic glucose and lipid metabolism as well as the susceptibility to MASLD induced by PPE in both male and female offspring, we cannot overlook the presence of sex differences. For example, significant liver fibrosis progression was observed in PPE male offspring, whereas this phenomenon was not observed in females. These results suggest that there is a sex difference in the susceptibility of PPE offspring to MASLD, with male offspring showing more severe MASLD phenotypic changes than female offspring do. This finding is consistent with previous epidemiologic reports of higher susceptibility to MASLD in males than in premenopausal females in the population.^112,113^ Similarly, in a model of exposure to dexamethasone (another synthetic glucocorticoid drug) during pregnancy, we previously reported sex differences in the alterations of cholesterol metabolism in offspring,^114,115^ more severe long bone dysplasia in males than in females,^116^ and islet β-cell dysfunction and glucose intolerance in male (but not female) offspring in the early postnatal period.^103^ Other laboratory investigations have similarly shown that gestational dexamethasone exposure leads to hypertension and renal diseases, particularly in male offspring.^117–122^ Collectively, these results suggest that prenatal glucocorticoid exposure may exert more pronounced effects on male offspring. We recently proposed that an epigenetic programming mechanism mediated by the sex hormone receptor *via* SP1/11β-HSD2 may mediate sex differences in postnatal adrenal steroidogenic function in offspring exposed to dexamethasone during pregnancy, which might be an important cause of sex differences in fetal-originated metabolic syndrome and related disorders caused by prenatal dexamethasone exposure.^123^ However, the causes and underlying mechanisms of sex differences caused by PPE during pregnancy are still poorly understood to date, warranting further in-depth investigations in future studies.

**Fig. 12:**
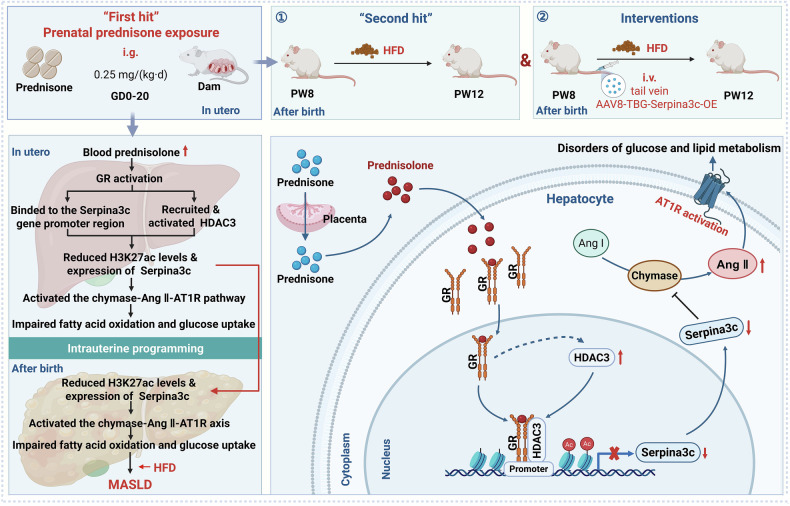
Serpina3c protects against MASLD in offspring induced by prenatal prednisone exposure *via* the inhibition of chymase-dependent Ang II production

In conclusion, we demonstrated for the first time that PPE contributes to hepatic glucose and lipid metabolic dysfunction and increases the susceptibility of offspring to MASLD (Fig. 12). We further confirmed that PPE activates hepatic GR-HDAC3 signaling *via* its active metabolite prednisolone. This leads to reduced H3K27ac levels in the *Serpina3c* promoter region and consequently inhibits Serpina3c expression, an effect that persists into the postnatal period. Sustained activation of the chymase-Ang II-AT1R pathway subsequently impaired both fatty acid oxidation and glucose uptake, ultimately resulting in hepatic metabolic dysfunction and increased susceptibility to MASLD in offspring. Importantly, this study also demonstrated that low expression of hepatic Serpina3c increased susceptibility to MASLD, whereas postnatal restoration of Serpina3c expression effectively ameliorated this susceptibility in PPE offspring. This study has significant practical value, as it elucidates the effects of PPE on the long-term metabolic health of offspring. Furthermore, this study reveals the potential programming mechanism underlying susceptibility to fetal-originated MASLD and identifies Serpina3c as a promising therapeutic target. These findings provide crucial insights for guiding the prevention and treatment of fetal-originated MASLD.

## Materials and methods

### Chemicals and reagents

Prednisone acetate tablets (No. H33021207) were purchased from Zhejiang Xianju Pharmaceutical Co., Ltd (Taizhou, China). Prednisolone (CAS. No. 50-24-8, Cat. No. MB1191, ≥98.5% purity) was purchased from Dalian Meilun Biotechnology Co., Ltd. (Dalian, Liaoning, China). The information of other chemicals and reagents used in this study were described in Supplementary Table 1. All of the other chemicals and reagents used in this study were of analytical grade and obtained commercially.

### Animals and treatment

Spathogen-free 9-week-old female Wistar rats (No. 110324210100821442) and 10-week-old male Wistar rats (No. 110324210100788048) were purchased from SPF (Beijing) Biotechnology Co., Ltd. (Beijing, China). Specific pathogen-free C57BL/6 mice (No. 410000000000003803) were purchased from Henan Skobes Biotechnology Co., Ltd (Anyang, Henan, China). All animal experimental procedures were reviewed and approved by the Institutional Animal Care and Use Committee (IACUC) of the Center for Animal Experiments of Wuhan University (IACUC NOS. WP20210060, WP20230087, and WP20230194). Animal experiments were performed at the Center for Animal Experiments/Animal Biosafety Level-III Laboratory of Wuhan University (Wuhan, Hubei, China), which is accredited by the Association for Assessment and Accreditation of Laboratory Animal Care International (AAALAC International). All animals were housed in the room with a 12-h dark/light cycle, controlled temperature (23 °C ± 2 °C) and humidity (55% ± 10%), and allowed free access to water and standard feed. Prednisone was suspended in 0.5% CMC-Na solution and administered orally to pregnant rats or mice.

The rat model of PPE was established according to our previous studies,^23,55^ as shown in Supplementary Fig. 1. All rats were acclimatized for one week. Subsequently, at approximately 18:00 each evening, rats were placed in the same cage at a female-to-male ratio of 2:1 for mating. The following morning at around 8:00, vaginal secretions from female rats were collected using a saline-moistened cotton swab for smear preparation, which was then examined under an optical microscope for the presence of sperm. The observation of sperm in the field of view was considered indicative of successful conception, and the day was designated as GD0. Pregnant rats were randomly assigned to either the control group or the PPE group. Pregnant rats in the PPE group received oral gavage administration of 0.25 mg/kg prednisone daily from GD0 to GD20, while those in the control group were given an equivalent volume (5 mL/kg) of vehicle (0.5% CMC-Na). For fetal rats: A subset of pregnant rats (n = 11 for control, n = 12 for PPE) was randomly selected from both groups to fast overnight. Then, they were euthanised after 2.5% isoflurane anesthesia on GD20. A cesarean section was then performed to obtain the fetal rats. Fetal rats were sexed (identified on the basis of anal to genital distance), anaesthetized with 2.5% isoflurane, and euthanised, and blood and liver tissue were collected. All samples were stored in a refrigerator at −80 °C for further indicator analysis. For postnatal rats: pregnant rats (n = 10/group) were subjected to spontaneous delivery. On the day of birth, pups were weighed, and litter sizes were recorded. Only litters with 10–16 pups were retained. To avoid nutritional imbalances due to different litter size, litter sizes were adjusted to 12 neonates (6 ♀ + 6 ♂). Body weight was recorded weekly. In PW3, pups were weaned and housed separately by gender. One male and one female offspring from each litter were selected and maintained on a NCD (11.85% fat, 65.08% carbohydrate, 23.07% protein, total 3.40 kcal/g; Beijing Keao Xieli Feed Co., Ltd, Beijing, China) until PW12. In addition, one male and one female offspring from each litter were fed NCD until PW8, and then switched to an HFD (D12492; 60% fat, 20% carbohydrates, 20% protein, total 5.24 kcal/g; Beijing Keao Xieli Feed Co.,Ltd, Beijing, China) until PW12. In PW12, intraperitoneal glucose tolerance test (IPGTT) and intraperitoneal insulin tolerance test (IPITT) were performed. After 2 days, all rats were fasted overnight and euthanised the next morning by anesthesia with 2.5% isoflurane, and blood and liver tissues were collected. All specimens were stored at -80 °C for further analysis. The remaining offspring rats from each litter were reserved for other experimental studies.

The experimental procedure for PPE in mice was similar to that of rats, as shown in supplementary Fig. 2. After 1 week of acclimatization feeding, male and female mice were mated. The day when a vaginal plug appeared was designated as GD0. All pregnant mice were randomly divided into 2 groups for the experiment. Pregnant mice in the PPE group were gavaged orally with 0.5 mg/kg of prednisone per day during pregnancy (GD0 to GD18). Pregnant mice in the control group were gavaged orally with an equal volume (5 mL/kg) of solvent (0.5% CMC-Na). Pregnant mice (n = 10) were subjected to spontaneous delivery. On delivery day, offspring were weighed and litter sizes recorded. Meanwhile, all litters were adjusted to 6 pups per dam to standardize nutrition. After weaning at PW3, male and female offspring housed separately by gender. One male and one female offspring from each litter were selected and maintained on an NCD (11.85% fat, 65.08% carbohydrate, 23.07% protein, total 3.40 kcal/g; Beijing Keao Xieli Feed Co. Ltd, Beijing, China) until PW12. In addition, one male and one female offspring from each litter were fed NCD until PW8, and then switched to an HFD (D12492; 60% fat, 20% carbohydrates, 20% protein, total 5.24 kcal/g; Beijing Keao Xieli Feed Co. Ltd, Beijing, China) until PW12. For Serpina3c overexpression, male offspring mice from both groups were selected at PW8. The recombinant adeno-associated virus overexpression vector (pAAV8-TBG-EGFP-P2A-Serpina3c-3xFLAG-tWPA) targeting Serpina3c in mouse liver and the empty vector (pAAV8-TBG-EGFP-tWPA) were constructed by Obio Technology (Shanghai) Corp., Ltd (Shanghai, China), and then injected into the mice *via* tail vein. A total amount of 2.5 × 10^11 ^v.g. virus (injection volume of 100 μL) was performed for hepatic Serpina3c overexpression. Subsequently, HFD (D12492; 60% fat, 20% carbohydrates, 20% protein, total 5.24 kcal/g; Beijing Keao Xieli Feed Co. Ltd, Beijing, China) was given to feed until PW12. In PW12, IPGTT and IPITT were performed. After 2 days, all offspring mice were fasted overnight and euthanised the next morning by anesthesia with 2.5% isoflurane, and blood and liver tissues were collected. All specimens were stored at −80 °C for further analysis.

Experimental procedure for liver-specific Serpina3c knockdown in mice was illustrated in supplementary Fig. 10. Twenty-eight male C57BL/6 mice were acclimatized for one week and randomly divided into 4 groups (n = 7): ①Empty vector + normal chow diet (AAV8-NC + NCD); ②Serpina3c knockdown + normal-chow diet (AAV8-Serpina3c-shRNA + NCD); ③Empty vector + high-fat diet (AAV8-NC + HFD); ④ Serpina3c knockdown + high-fat diet (AAV8-Serpina3c-shRNA + HFD). Mouse liver-specific knockdown of Serpina3c in AAV (AAV8-GP-1-Serpina3c-mus-227) and empty vector (AAV8-GP-1-SJ-NC) were purchased from Shanghai GenePharma Co. Ltd (Shanghai, China). A mouse model of liver-specific Serpina3c silencing was established by injecting a total amount of 2.5 × 10^11 ^v.g. of virus (injection volume of 100 μL) into mice *via* tail vein. The shRNA sequence targeting mouse Serpina3c was: 5’-GGACACAACTCGACAGTCTCA-3’, with the control sequence: 5’-ACTACCGTTCTTATAGGTG-3’. IPGTT and IPITT tests were performed after 4 weeks of feeding on an NCD or HFD. After 2 days, all mice were fasted overnight and euthanised the next morning by anesthesia with 2.5% isoflurane, and blood and liver tissues were collected. All samples were stored at −80 °C for further analysis.

### Body weights and body weight gain rate

From GD0, the pregnant dams were weighed daily using electronic scales. For offspring, on the day of birth (i.e., PW0), pups were weighed and recorded, then weighed weekly thereafter. Body weight gain rate refers to the percentage increase or decrease in body weight over a specific period. The calculation formula is: Body weight gain rate (%) = (BW_current weight_ − BW_initial weight_)/BW_initial weight_ × 100. Thus, body weight gain rate (%) = (BW_GDX_ – BW_GD0_)/BW_GD0_ × 100 for pregnant dams, body weight gain rate (%) = (BW_PWX_ – BW_PW0_)/BW_PW0_ × 100 for offspring.

### Cell culture and treatment

AML-12 cells (an immortalized normal mouse hepatocyte cell line) and HepG2 cells (a human hepatocellular carcinoma cell line) were obtained from the China Center for Type Culture Collection (CCTCC). AML12 cells were cultured in DMEM/F-12 medium supplemented with 10% fetal bovine serum (FBS), 1% insulin-transferrin-selenium supplement (100×), 40 ng/mL dexamethasone, 100 U/mL penicillin, and 100 μg/mL streptomycin. HepG2 cells were cultured in DMEM medium containing 10% FBS, 100 U/mL penicillin, and 100 μg/mL streptomycin. Our previous study found that the serum concentration of prednisolone, the active metabolite of prednisone, in fetal rats in PPE group ranged from 4.1 to 57.2 nmol/L.^23^ After seeding AML12 and HepG2 cells in 6-well plates overnight (~12 h), both hepatocyte lines (AML12 and HepG2 cells) were treated with different concentrations (2, 10, and 50 nmol/L) of prednisolone for 72 h according to our preliminary study.

To better assess cellular TG content and perform oil red O staining, oleic acid (OA, final concentration 0.1 mmol/L) was added 24 h before cell collection. To investigate cellular responsiveness to chymase, cells were stimulated with chymase (final concentration 100 ng/mL) for 24 h prior to harvesting. To examine the role of Serpina3c in AML12 cells, knockdown experiments were performed using Lipofectamine™ 3000 transfection reagent to transfect Serpina3c siRNA for 6 h and then the medium was changed before subsequent experiments. For overexpression studies, AML12 cells were transfected with constructed Serpina3c overexpression plasmids [pcDNA3.1(+)-mouse-Serpina3c] using Lipofectamine™ 3000 and P3000™ transfection reagents for 6 h, followed by drug treatment.

### Plasmid and siRNA transfection assays

For plasmid overexpression experiments: The mouse Serpina3c overexpression plasmid and control plasmid were constructed by Obio Technology (Shanghai) Corp., Ltd (Shanghai, China). AML12 cells were transfected with the plasmids using Invitrogen™ Lipofectamine 3000 transfection reagents (Cat. No. L3000015; Thermo Fisher Scientific Inc., Waltham, MA, USA) under serum-free conditions, following the manufacturer’s protocol. After 6 h of incubation, the medium was replaced for subsequent experimental treatments. For siRNA knockdown experiments: Mouse Serpina3c siRNA, human NR3C1 siRNA, human HDAC3 siRNA and their corresponding control siRNAs were purchased from Shanghai GenePharma Co. Ltd (Shanghai, China). Detailed sequences were provided in Supplementary Table 2. For Serpina3c knockdown, AML12 cells were transfected with siRNA using Invitrogen™ Lipofectamine 3000 transfection reagents (Cat. No. L3000015; Thermo Fisher Scientific Inc., Waltham, MA, USA) under serum-free conditions, following the manufacturer’s protocol. For NR3C1 or HDAC3 knockdown, HepG2 cells were transfected with siRNA using Lipofectamine™ 3000 under serum-free conditions, following the manufacturer’s protocol. After 6 h of incubation, the medium was replaced for subsequent experimental treatments.

### Luciferase reporter assays

Plasmids pcDNA3.1(+)-mouse-GR(/NR3C1), pcDNA3.1(+)-NC, pGL4-basic, pGL4-mouse-Serpina3c-promoter and pRL-TK were purchased from Shanghai GenePharma Co. Ltd (Shanghai, China). Luciferase reporter assay of GR-Serpina3c was performed as described in the reference.^124,125^ The AML12 cell lines were seeded into a 96-well culture plate the day prior to transfection. Cells were transfected with the plasmids using Invitrogen™ Lipofectamine 3000 transfection reagents (Cat. No. L3000015; Thermo Fisher Scientific Inc., Waltham MA, USA) under serum-free conditions, following the manufacturer’s protocol. The transfection plasmid combinations were as follows: ① pGL4-basic + pcDNA3.1(+)-NC + pRL-TK; ② pGL4-basic + pcDNA3.1(+)-mouse-GR(/NR3C1) + pRL-TK; ③ pGL4- mouse-Serpina3c-promoter + pcDNA3.1(+)-NC + pRL-TK; ④ pGL4-mouse-Serpina3c-promoter + pcDNA3.1(+)-mouse-GR(/NR3C1) + pRL-TK. After 6 h transfection, the transfected AML12 cells were treated with/without prednisolone (50 μmol/L) for 72 h. The luciferase activity was assayed with a Dual-Luciferase® Reporter (DLR™) Assay System [no. E1910, Promega (Beijing) Biotech Co. Ltd, Beijing, China] using GloMax® 20/20 luminometer and was normalized to the Renilla luciferase activity.

### 2-deoxy-2- [(7-nitro-2,1,3-benzoxadiazol-4-yl) amino]-D-glucose (2-NBDG) uptake assays

Cells were seeded in a 96-well plate and subjected to drug treatment. Following treatment, each well was replaced with 100 μL of glucose-free, serum-free culture medium containing the respective drug and incubated for 3 h under starvation conditions. The cells were then washed twice with phosphate buffer saline (PBS) and incubated with 100 μL of glucose-free, serum-free culture medium containing 2-NBDG (100 mmol/L) for 30 min. Subsequently, the culture medium was removed, and the wells were washed twice with PBS before adding 100 μL of PBS. Fluorescence intensity of 2-NBDG was measured using a multifunctional microplate reader with excitation wavelength set at 468 nm and emission wavelength set at 524 nm.

### IPGTT and IPITT

IPGTT and IPITT were performed as previously described.^126–128^ In brief, for IPGTT, offspring rats and mice in PW12 were fasted overnight (~16 h) and then intraperitoneally injected with 20% glucose solution at a dose of 2 g/kg. At 0, 15, 30, 60, and 120 min after glucose administration, blood glucose levels were measured from tail vein blood by using Roche Accu-Chek® Performa glucose meter. For IPITT, offspring rats and mice in PW12 were fasted for 6 h and then intraperitoneally injected with insulin at a dose of 0.75 U/kg. Blood glucose levels were measured as above at the same time intervals. To eliminate baseline glucose variations between animal individuals, we normalized the blood glucose levels at each time point to the 0-minute value for both IPGTT and IPITT. Glucose response curves were plotted and the area under the curve (AUC) was calculated using the trapezoidal rule. The formula for AUC calculation was as follows:

$${\rm{AUC}}=\frac{{(C}_{0}+{C}_{15})}{2}\times {{Time}}_{0-15}+\frac{{(C}_{15}+{C}_{30})}{2}\times {{Time}}_{15-30}+\frac{{(C}_{30}+{C}_{60})}{2}\times {{Time}}_{30-60}+\frac{{(C}_{60}+{C}_{120})}{2}\times {{Time}}_{60-120}$$

${C}_{X}$: Blood glucose value at each time point (mmol/L); Time: Time inter (min)

### Biochemical and ELISA assays

For blood samples: Following complete coagulation, whole blood was centrifuged at 3500 rpm for 15 min at 4 °C to obtain serum, which was subsequently stored at −80 °C until analysis. Serum glucose and TG levels were measured using specific biochemical assay kits (Cat. No. F006-1-1, A110-1-1; Nanjing Jiancheng Bioengineering Institute, Nanjing, Jiangsu, China) according to the manufacturers’ instructions. Serum Serpina3c (α1-antichymotrypsin) concentrations were determined using the ELISA kits (Cat. No. RA22998; Bioswamp® Life Science Lab, Wuhan, Hubei, China) following the manufacturer’s protocols. Serum insulin concentrations were determined using an ultrasensitive insulin ELISA kit (Rat: Cat. No. 10-1251-01; mouse: Cat. No. 10-1249-01; Mercodia, Uppsala, Sweden) following the manufacturer’s protocols. Insulin resistance was calculated using the HOMA-IR method. The calculation formula for HOMA-IR is as follows:

$${\rm{HOMA}}-{\rm{IR}}=\frac{{\rm{F}}{\rm{asting\; insulin}}({\rm{\mu }}{\rm{IU}}/{\rm{mL}})\times {\rm{F}}{\rm{asting\; glucose}}({\rm{mmol}}/{\rm{L}})}{22.5}$$

For liver tissue and cell samples: Liver tissues and cell samples were homogenized in ice-cold PBS and subjected to ultrasonic disruption according to the manufacturer’s instructions. The homogenates were then centrifuged at 2500 rpm for 10 min at 4 °C. The resulting supernatants were used for determining hepatic TG and Ang II content by TG assay kit (Cat. No. A110-1-1; Nanjing Jiancheng Bioengineering Institute, Nanjing, Jiangsu, China) and Ang Ⅱ ELISA kit (Rat: Cat. No. JYM0668Ra; mouse: Cat. No. JYM0080Mo; Wuhan JYMBio Technology Co., Ltd., Wuhan, Hubei, China). And then data were normalized to the total protein content. Protein concentrations were quantified using the bicinchoninic acid (BCA) protein assay kit (Cat. No. P0010S; Beyotime Biotechnology, Shanghai, China).

### Histological analysis

Histopathological analyses were performed as previously described.^21,129^ Briefly, liver tissues (n = 5) were fixed in 4% paraformaldehyde, dehydrated, and embedded in paraffin. The paraffin-embedded liver sections were then cut into 4-μm thick slices for hematoxylin-eosin (HE), periodic acid-Schiff (PAS) and Sirius red staining. For oil red O staining, liver tissues were dehydrated in sucrose solution and embedded in optimal cutting temperature (OCT) compound, followed by cryosectioning at 10-μm thickness. Both tissue sections and 4% paraformaldehyde-fixed cell samples were stained using a commercial oil red O staining kit (Cat. No. G1015; Wuhan Servicebio Technology Co., Ltd., Wuhan, Hubei, China) according to the manufacturer’s protocol. Microscopic images were captured and processed using NIS-Elements Br 4.20 software (Nikon, USA). Histopathological assessments, including NAS and liver fibrosis scores (supplementary Table 3), were conducted in accordance with the guidelines established by the Nonalcoholic Steatohepatitis Clinical Research Network (NASH CRN) system.^130,131^ NAS is defined as a composite score ranging from 0 to 8 that assesses the histologic features, incorporating steatosis, hepatocyte ballooning, and lobular inflammation levels. When the NAS is > 5 points, it can be diagnosed as NASH, NAS of 3–4 points indicates suspected non-NASH, NAS < 3 points excludes the diagnosis of NASH.

### Immunofluorescence (IF) assays

For tissue sections: Paraffin-embedded liver tissues were sectioned at 4-μm thickness for IF staining, performed as previously described.^21^ Briefly, the liver paraffin sections were sequentially deparaffinized to water, antigenically repaired and serum blocked, then incubated with primary antibody (Anti-Glucocorticoid Receptor antibody, 1:200 dilution) overnight. Subsequently, the sections were incubated with secondary antibody (1:200 dilution) for 1 h under the protection of light, and then counterstained with DAPI staining solution. For cell samples: Cells grown on coverslips were fixed with 4% paraformaldehyde for 15 min at 4 °C, then sequentially permeabilized with 0.2% Triton X-100 and sealed with 3% BSA, incubated with primary antibody (Anti-Glucocorticoid Receptor antibody, 1:200 dilution) overnight. Subsequently, samples were incubated with secondary antibody (1:200 dilution) for 1 h in the dark, then counterstained with DAPI. Fluorescence images were captured and analyzed using an Olympus AH-2 fluorescence microscope (Olympus, Tokyo, Japan).

### mRNA-seq analysis

mRNA-seq of fetal rat livers was done by Novogene Co., Ltd (Beijing, China). The specific method is as follows.

Total RNA was isolated from fetal rat livers on GD20. RNA integrity was assessed using the RNA Nano 6000 Assay Kit of the Bioanalyzer 2100 system (Agilent Technologies, CA, USA). A total amount of 1 μg RNA per sample was used as input material for the RNA sample preparations. Briefly, mRNA was purified from total RNA using poly-T oligo_-_attached magnetic beads. Fragmentation was caried out using divalent cations under elevated temperature in First Strand Synthesis Reaction Buffer (5×). First strand cDNA was synthesized using random hexamer primer and M-MuLV Reverse Transcriptase (RNase H-). Second strand cDNA synthesis was subsequently performed using DNA Polymerase I and RNase H. Remaining overhangs were converted into blunt ends *via* exonuclease/polymerase activities. After adenylation of 3 ends of DNA fragments, Adaptor with hairpin loop structure were ligated to prepare for hybridization_._ In order to select cDNA fragments of preferentially 370–420 bp in length, the library fragments were purified with AMPure XP system (Beckman Coulter, Beverly, USA). Then PCR was performed with Phusion High_-_Fidelity DNA polymerase, Universal PCR primers and Index (X) Primer. At last, PCR products were purified (AMPure XP system) and library quality was assessed on the Agilent Bioanalyzer 2100 system. The clustering of the index_-_coded samples was performed on a cBot Cluster Generation System using TruSeq PE Cluster Kit v3-cBot-HS (llumia) according to the manufacturer’s instructions. After cluster generation, the library preparations were sequenced on an Ⅱ umina Novaseq platform and 150 bp paired-end reads were generated.

Raw data (raw reads) offastq format were firstly processed through in-house Perl scripts. In this step, clean data (clean reads) were obtained by removing reads containing adapter, reads containing ploy-N and low quality reads from raw data. At the same time, Q20, Q30 and GC content the clean data were calculated. All the downstream analyses were based on the clean data with high quality. Reference genome and gene model annotation files were downloaded from genome website directly. Index of the reference genome was built using Hisat2 v2.0.5and paired-end clean reads were aligned to the reference genome using Hisat2 v2.0.5. We selected Hisat2 as the mapping tool for that Hisat2 can generate a database of splice junctions based on the gene model annotation file and thus a better mapping result than other non-splice mapping tools. featureCounts v1.5.0-p3 was used to count the reads numbers mapped to each gene. And then FPKM of each gene was calculated based on the length of the gene and reads count mapped to this gene. FPKM, expected number of Fragments Per Kilobase of transcript sequence per Millions base pairs sequenced, considers the effect of sequencing depth and gene length for the reads count at the same time, and is currently the most commonly used method for estimating gene expression levels. Differential expression analysis of two groups was performed using the DESeq2 R package (1.20.0). DESeq2 provide statistical routines for determining differential expression in digital gene expression data using a model based on the negative binomial distribution. Genes with a *P*-value < 0.05 and |log2foldchange| > 1 were considered DEGs. KEGG enrichment analysis of DEGs was conducted using the clusterProfiler R package.

The raw sequence data from mRNA-seq reported in this paper have been deposited in the Genome Sequence Archive^132^ in the National Genomics Data Center,^133^ China National Center for Bioinformation / Beijing Institute of Genomics, Chinese Academy of Sciences (GSA: CRA034707) that are publicly accessible at https://ngdc.cncb.ac.cn/gsa.

### Evaluation of *serpina3c* promoter DNA methylation with Next generation sequencing-based BSP

*Serpina3c*-specific DNA methylation was assessed by a next generation sequencing-based BSP in Wuhan GeneRead Biotechnology Co., Ltd. (Wuhan, Hubei, China), according to previously published method.^134–137^ In brief, BSP primers were designed using the online MethPrimer software (http://www.urogene.org/methprimer/index1.html) and listed in supplementary Table 4. Genomic DNA (1 μg) was converted using the ZYMO EZ DNA Methylation-Gold Kit (Zymo Research, lrvine, CA, USA) and one twentieth of the elution products were used as templates for PCR amplification with 35 cycles using KAPA HiFi HotStart Uracil+ ReadyMix PCR Kit (Kapa Biosystems, Wilmington, MA, USA). For each sample, BSP products of multiple genes were pooled equally, 5′-phosphorylated, 3′-dA-tailed and ligated to barcoded adapter using T4 DNA ligase (NEB). Barcoded libraries from all samples were sequenced on lllumina platform.

### Reverse-transcription quantitative real-time PCR (RT-qPCR) assays

RT-qPCR analysis was performed as previously described.^103,124,138^ Briefly, total RNA extraction, cDNA synthesis, and qPCR analysis were conducted using TRIzol Reagent (Cat.No. 15596026; Thermo Fisher Scientific Inc., Waltham MA, USA), HiScript III ^RT^ SuperMix for qPCR (+gDNA wiper) (Cat. No. R323-01; Vazyme Biotech Co. Ltd, Nanjing, Jiangsu, China), and Taq Pro Universal SYBR qPCR Master Mix (Cat. No. Q712-02; Vazyme Biotech Co. Ltd, Nanjing, Jiangsu, China), respectively, following the manufacturers’ protocols. The qPCR reactions were performed on a QuantStudio^®^ 5 Real-Time PCR System (Thermo Fisher Scientific Inc., Waltham, MA, USA) to obtain Ct values. The relative expression levels of target genes were calculated using the 2^−ΔΔCt^ method with glyceraldehyde-3-phosphate dehydrogenase (GAPDH) as the internal reference gene. All primers were synthesized by Wuhan Tianyi Huayu Gene Technology Co., Ltd (Wuhan, Hubei, China), with sequences listed in Supplementary Table 5.

### Chromatin immunoprecipitation-quantitative PCR (ChIP-qPCR) assays

As previously described,^139,140^ liver tissue or cell homogenates were cross-linked with formaldehyde, followed by ChIP using specific antibodies (H3K9ac, H3K14ac, H3K27ac, or GR antibody). Total chromatin was used as the input. The isolated DNA was then subjected to qPCR with serpina3 promoter primers (Forward: 5′- GAAATCATCCCGTCTGCCCA-3′; Reverse: 5′-CTGAGTCTCATGGGTTGGCC-3′). qPCR reactions were performed on a QuantStudio® 5 Real-Time PCR System (Thermo Fisher Scientific Inc., Waltham, MA, USA) to obtain Ct values. Negative control (IgG) values were subtracted as background, and the input values were normalized to their corresponding values of IP samples following the formula (IP/input = 2^Ct input DNA^ – Ct^IP DNA^).

### Co-IP assays

The co-IP analysis was performed as previously described.^104,140^ Briefly, cells were lysed on ice for 30 min using Cell Complete Lysis Buffer for Western and IP (Cat. No. P0037; Beyotime Biotechnology, Shanghai, China) containing protease inhibitor cocktail. After centrifugation (12,000 rpm, 4 °C), the supernatant was incubated with the target antibody (GR or HDAC3 antibody) overnight at 4 °C on a rotator mixer, followed by a 2-h incubation with rProtein A/G Beads 4FF to immunoprecipitate the protein of interest. The precipitates were then washed with cell lysis buffer and boiled for 10 min in 1× sodium dodecyl sulfate-polyacrylamide gel electrophoresis (SDS-PAGE) sample loading buffer. The supernatant was collected for subsequent SDS-PAGE analysis.

### WB analysis

WB analysis was performed as previously described.^103,124^ In brief, total proteins were extracted from liver tissues (50 μg) or cells using RIPA lysis buffer containing both protease inhibitor cocktail and phosphatase inhibitor cocktail. Nuclear and cytoplasmic proteins were extracted using commercial kits according to manufacturer’s instructions. Protein concentrations were quantified with the enhanced BCA protein assay kit according to the manufacturer’s instructions. Then, the protein supernatant was collected and added to an equal volume of 2× SDS-PAGE sample loading buffer, followed by boiling for 10 min to ensure complete protein denaturation. Samples were stored at −20 °C for subsequent SDS-PAGE analysis. Protein samples were separated on 10% or 12% SDS-PAGE gels prepared with a Omni-Easy™ one-step color PAGE gel rapid preparation kit and transferred onto polyvinylidene fluoride (PVDF) membranes. PVDF membranes were blocked with protein free rapid blocking buffer (1×) for 15 min at room temperature, then incubated overnight at 4 °C with primary antibodies diluted in primary antibody dilution buffer for WB (GAPDH: 1:5000; other primary antibodies: 1:1000). After tris-buffered saline with tween 20 (TBST) washes, membranes were incubated with corresponding secondary antibody conjugated with horseradish peroxidase (1:5000 dilution in TBST) for 1 h at room temperature. Specific targeting protein bands were visualized using an ECL kit on a Tanon 5200 Chemiluminescence Imaging System (Tanon, Shanghai, China) or Amersham ImageQuant ™ 800 System (Cytiva, Washington, D.C., USA), and further analyzed using Image Pro Plus 6.0 (Media Cybernetics, MD, USA).

### Statistical analyses

The experimental data are presented as mean ± standard error of mean (S.E.M.) and were statistically analyzed and plotted using IBM SPSS Statistics 20 (SPSS Science Inc., Chicago, IL, USA) and GraphPad Prism 9.0 (GraphPad Software, La Jolla, CA, USA). After assessing normality and homogeneity of variance, unpaired Student’s *t*-test and one-way analysis of variance (ANOVA) were employed for comparisons between two groups and multiple groups with normally distributed and equal-variance data, respectively. For normally distributed data with unequal variances, Welch’s correction was applied. For data in certain two groups that did not conform to a normal distribution, nonparametric test (Mann-Whitney U test) was employed for comparisons. Two-way repeated measures ANOVA was used to evaluate continuous time-point data (i.e., body weight during PW0−12, blood glucose levels in IPGTT and IPITT). Values of *P* < 0.05 (two-tailed) were considered to be statistically significant.

## Supplementary information

Supplementary material

## Data Availability

The raw sequence data from mRNA-seq reported in this paper have been deposited in the Genome Sequence Archive in the National Genomics Data Center, China National Center for Bioinformation / Beijing Institute of Genomics, Chinese Academy of Sciences (GSA: CRA034707) which are publicly accessible at https://ngdc.cncb.ac.cn/gsa. The expression profiling by array from MASLD patients and the healthy population cited in the study is accessible through a public repository hosted on the Gene Expression Omnibus (GEO: GSE48452) (https://www.ncbi.nlm.nih.gov/geo/query/acc.cgi?acc=GSE48452). Additional data related to this study can be obtained from the corresponding author upon reasonable request.
