# Supplementary material for: Serpina3c protects against metabolic dysfunction-associated steatotic liver disease in offspring induced by prenatal prednisone exposure
Source: Signal Transduct Target Ther. 2026 Feb 18;11:71. doi: 10.1038/s41392-025-02569-1 (PMC12917113; doi:10.1038/s41392-025-02569-1)

Supplementary Materials for

**Serpina3c protects against metabolic dysfunction-associated steatotic liver disease in offspring induced by prenatal prednisone exposure**

Yongguo Dai ^1, 4, #^, Zhengjie Lu ^1, 2, 5, #^, Yu Peng ^1^, Kexin Liu ^1, 3^, Xiaoqian Lu ^1^, Xiaoxiang Sun ^1^, Yuxi Wang ^1^, Xuerong Yan ^1^, Zijie Chen ^1^, Ziyi Zhang ^1^, Ning Zhang ^1^, Aihemaitijiang Ailikaiti ^1^, Yiming Chen ^1^, Quanrui Yue ^1^, Yu Guo ^1, 3^, Liaobin Chen ^2, 3^, Hui Wang ^1, 3,*^

**^#^ Yongguo Dai and Zhengjie Lu contributed equally to this study.**

Correspondence to: wanghui19@whu.edu.cn.

**This PDF file includes:**

Supplementary Figs. 1 to 16

Supplementary Tables 1 to 5

Supplementary original images of western blot bands

**Supplementary figures**


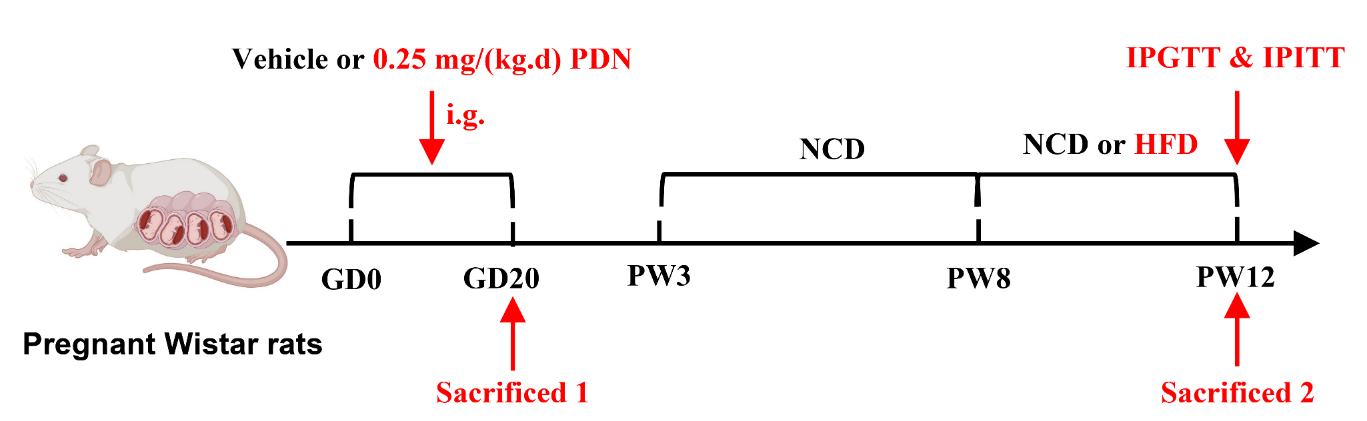


**Supplementary Fig. 1 Experimental procedures in rats exposed to prednisone during pregnancy.** GD, gestational day; HFD, high-fat diet; i.g., intragastrical administration; IPGTT, intraperitoneal glucose tolerance test; IPITT: intraperitoneal insulin tolerance test; NCD, normal-chow diet; PDN, prednisone; PW, postnatal week.


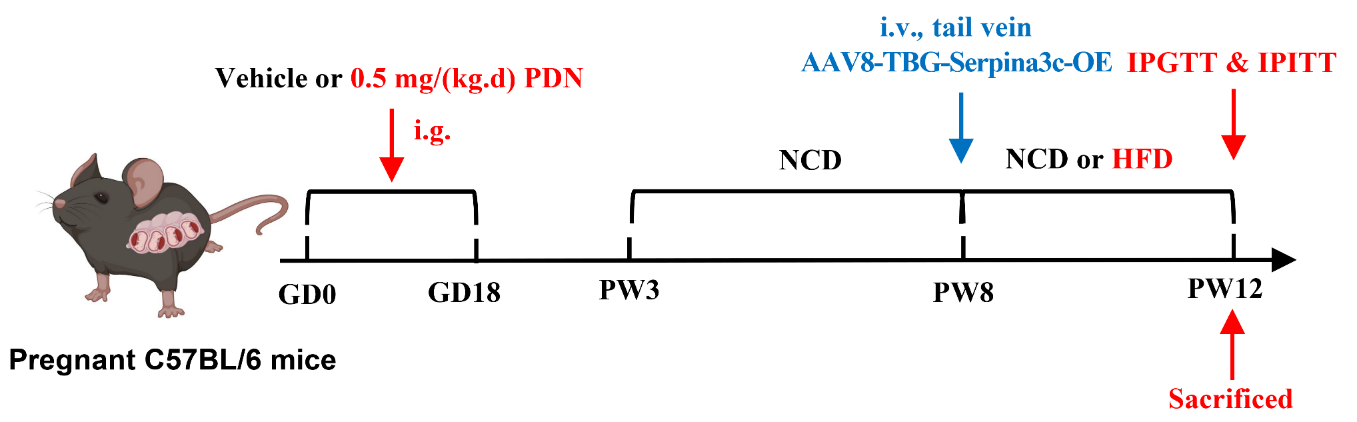


**Supplementary Fig. 2** **Experimental procedures in mice exposed to prednisone during pregnancy.** AAV8, adeno-associated virus serotype 8; GD, gestational day; HFD, high-fat diet; i.g., intragastrical administration; IPGTT, intraperitoneal glucose tolerance test; IPITT: intraperitoneal insulin tolerance test; i.v., intravenous injection; NCD, normal-chow diet; OE, overexpression; PDN, prednisone; PW, postnatal week; TBG, thyroxine-binding globulin.


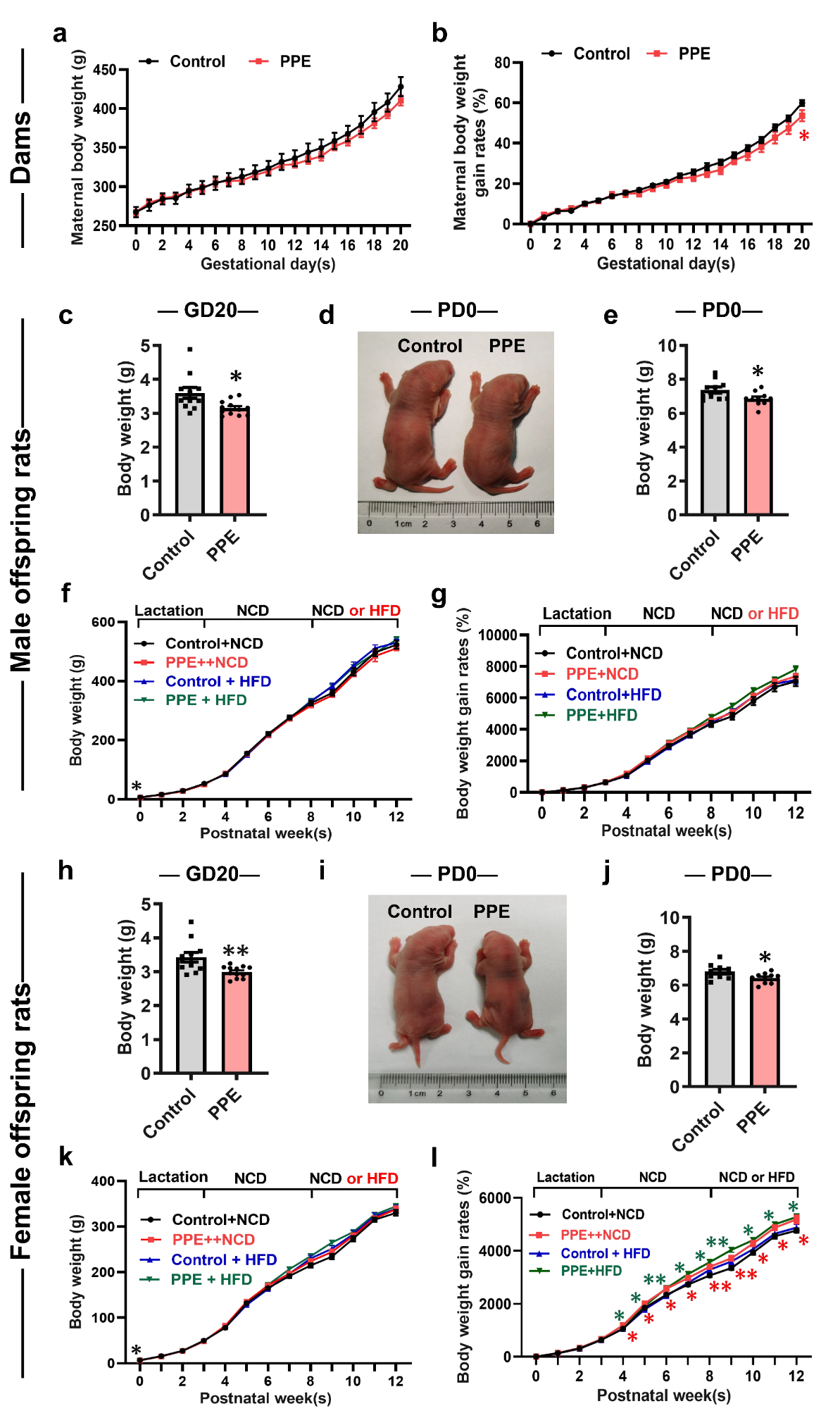


**Supplementary Fig. 3 The effect of PPE on the body weights and body weight gain rates in dams and offspring rats.** Pregnant Wistar rats were intragastrically administrated with prednisone (0.25 mg/kg) or vehicle control (0.5% CMC-Na) per day from GD0-20, and then offspring rats of different ages (GD20 and PW12) were obtained for further analysis. Among these offspring, part of them were fed with HFD (D12492) during PW8-12. Body weight gain rate (%) = (BW_GDX_ – BW_GD0_) / BW_GD0_ × 100 for pregnant dams, body weight gain rate (%) = (BW_PWX_ – BW_PW0_) / BW_PW0_ × 100 for offspring. (a, b) Body weights and body weight growth rates of dams during GD0-20; (c, h) Body weights of male and female fetal rats on GD20; (d, i) Representative photos of male and female neonatal rats on PD0; (e, j) Body weights of male and female neonatal rats on PD0; (f, k) Body weights of male and female offspring rats during PW0-12; (g, l) Body weight growth rates of male and female offspring rats during PW0-12. Mean ± S.E.M., n = 11-12 for data on GD20, n = 10 for data after birth. Statistical significance was determined by two-tailed unpaired Student’s *t*-test (B1−B3, C1−C3), and two-way ANOVA for repeated measures followed by Bonferroni's post-hoc test (A1, A2, B4, B5, C4, C5). ^⁎^*P <* 0.05, ^⁎⁎^*P <* 0.01 *vs.* control. GD, gestational day; HFD, high-fat diet; NCD, normal-chow diet; PD, postnatal day; PPE, prenatal prednisone exposure; PW, postnatal week.


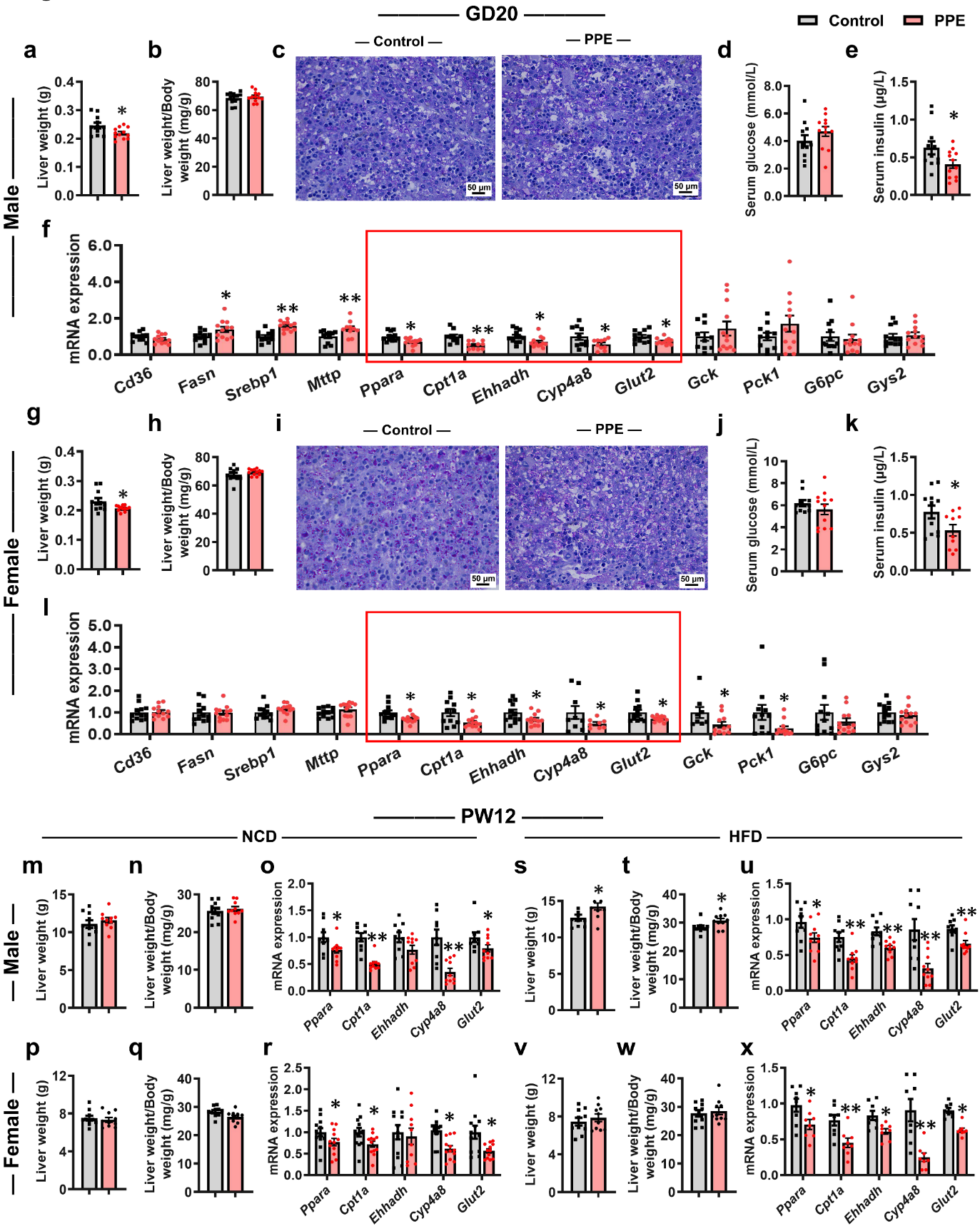


**Supplementary Fig. 4 The effect of PPE on liver weights and expression of genes related to hepatic glucose and lipid metabolism in male and female offspring rats.** Pregnant Wistar rats were intragastrically administrated with prednisone (0.25 mg/kg) or vehicle control (0.5% CMC-Na) per day from GD0-20, and then offspring rats of different ages (GD20 and PW12) were obtained for further analysis. Among these offspring, part of them were fed with HFD (D12492) during PW8-12. (a, g, m, p, s, v) Liver weights; (b, h, n, q, t, w) Liver weight/body weight; (c, i) Representative micrographs of liver sections stained with PAS staining, scale bar: 50 μm; (d, j) Serum glucose levels; (e, k) Serum insulin levels; (f, l, o, r, u, x) mRNA expression of hepatic glucose and lipid metabolism-related genes (*Cd36*, *Fasn*, *Srebp1*, *Ppara*, *Cpt1a*, *Ehhadh*, *Cyp4a8*, *Glut2*, *Gck*, *Pck1*, *G6pc* and *Gys2*). Mean ± S.E.M., n = 11-12 for data on GD20, n = 10 for data in PW12. Statistical significance was determined by two-tailed unpaired Student’s *t*-test (or two-tailed unpaired Student’s *t*-test with Welch’s correction). ^⁎^*P <* 0.05, ^⁎⁎^*P <* 0.01 *vs.* control. *Cd36*, cluster of differentiation 36; *Cpt1a*, carnitine palmitoyltransferase 1α; *Ehhadh*, enoyl-CoA hydratase and 3-hydroxyacyl CoA dehydrogenase; *Fasn*, fatty acid synthase; *G6pc*, glucose-6-phosphatase; *Gck*, glucokinase; GD, gestational day; *Glut2*, glucose transporter 2; *Gys2*, glycogen synthase 2; HFD, high-fat diet; *Mttp*, microsomal triglyceride transfer protein; NCD, normal-chow diet; *Pck1*, phosphoenolpyruvate carboxykinase 1; *Ppara*, peroxisome proliferators activated receptor α; PPE, prenatal prednisone exposure; *Srebp1*; PW, postnatal week; sterol-regulatory element binding protein-1.


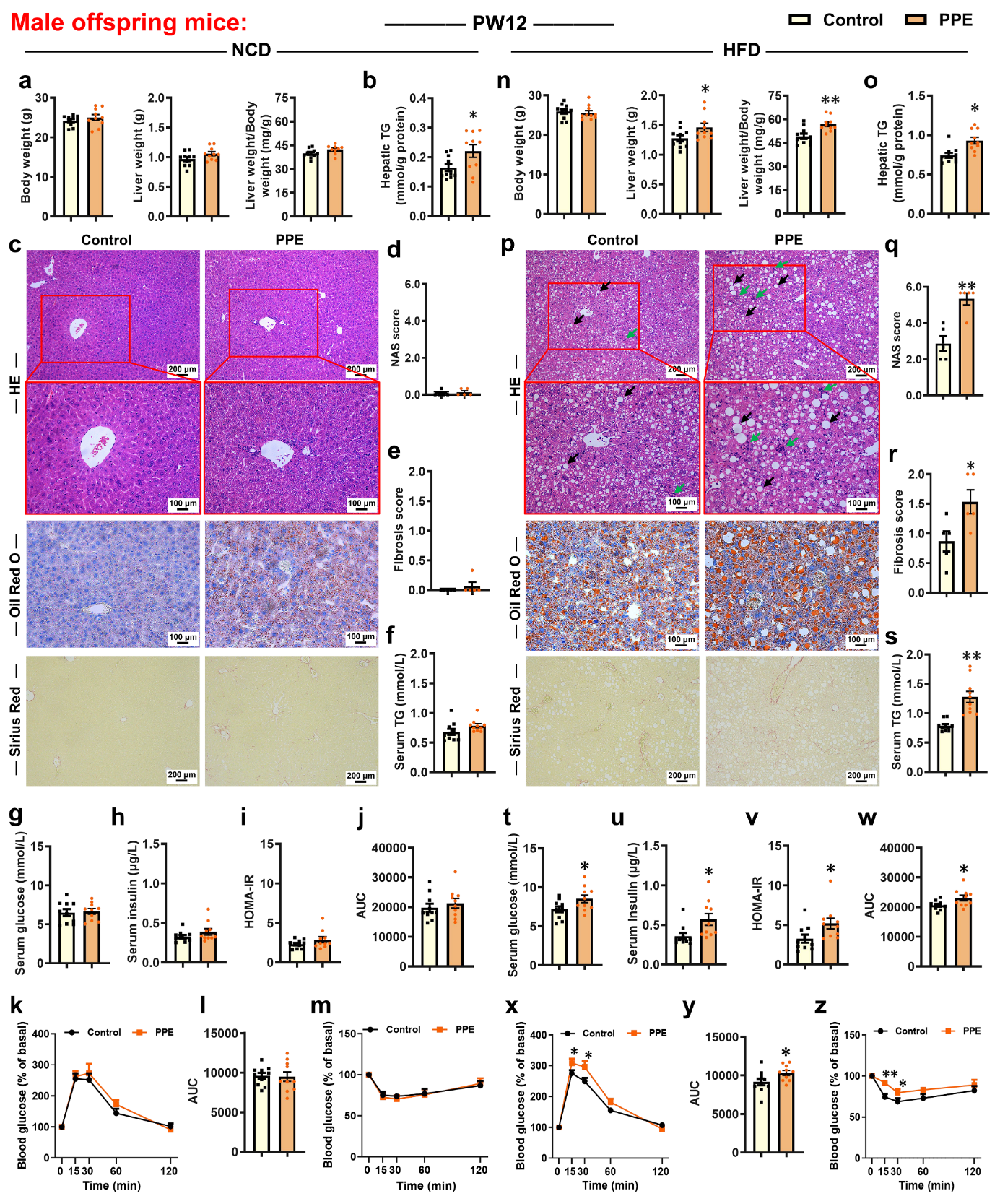


**Supplementary Fig. 5 The effect of PPE on** **susceptibility to MASLD in male offspring mice.** Pregnant C57BL/6 mice were intragastrically administrated with prednisone (0.5 mg/kg) or vehicle control (0.5% CMC-Na) per day from GD0-18, and then offspring mice in PW12 were obtained for further analysis. Among these offspring, part of them were fed with HFD (D12492) during PW8-12. (a, n) Body weights, liver weights and liver weight/Body weight; (b, o) Hepatic TG content; (c, p) Representative micrographs of liver sections stained with HE, oil red O and sirius red staining, scale bar: 100 μm and 200 μm; (d, q) Liver NAS score; (e, r) Liver fibrosis score; (f, s) Serum TG levels; (g−i, t−v) Fasting serum glucose, insulin and HOMA-IR index; (j, k, w, x) Normalized blood glucose levels during IPGTT and corresponding AUC; (l, m, y, z) Normalized blood glucose levels during IPITT and corresponding AUC. Mean ± S.E.M., n = 5 for histopathological data, n = 10 for others data. Statistical significance was determined by two-tailed unpaired Student’s *t*-test (a−j, l, n−w, y) and two-way ANOVA for repeated measures followed by Bonferroni’s post-hoc test (k, m, x, z). ^⁎^*P <* 0.05, ^⁎⁎^*P <* 0.01 *vs.* control. AUC, area under the curve; CMC-Na, carboxymethyl cellulose sodium; HE, hematoxylin eosin; HFD, high-fat diet; HOMA-IR, homeostatic model assessment of insulin resistance; IPGTT, intraperitoneal glucose tolerance test; IPITT, intraperitoneal glucose tolerance test; NCD, normal-chow diet; PPE, prenatal prednisone exposure; PW, postnatal week; TG, triglyceride.

**
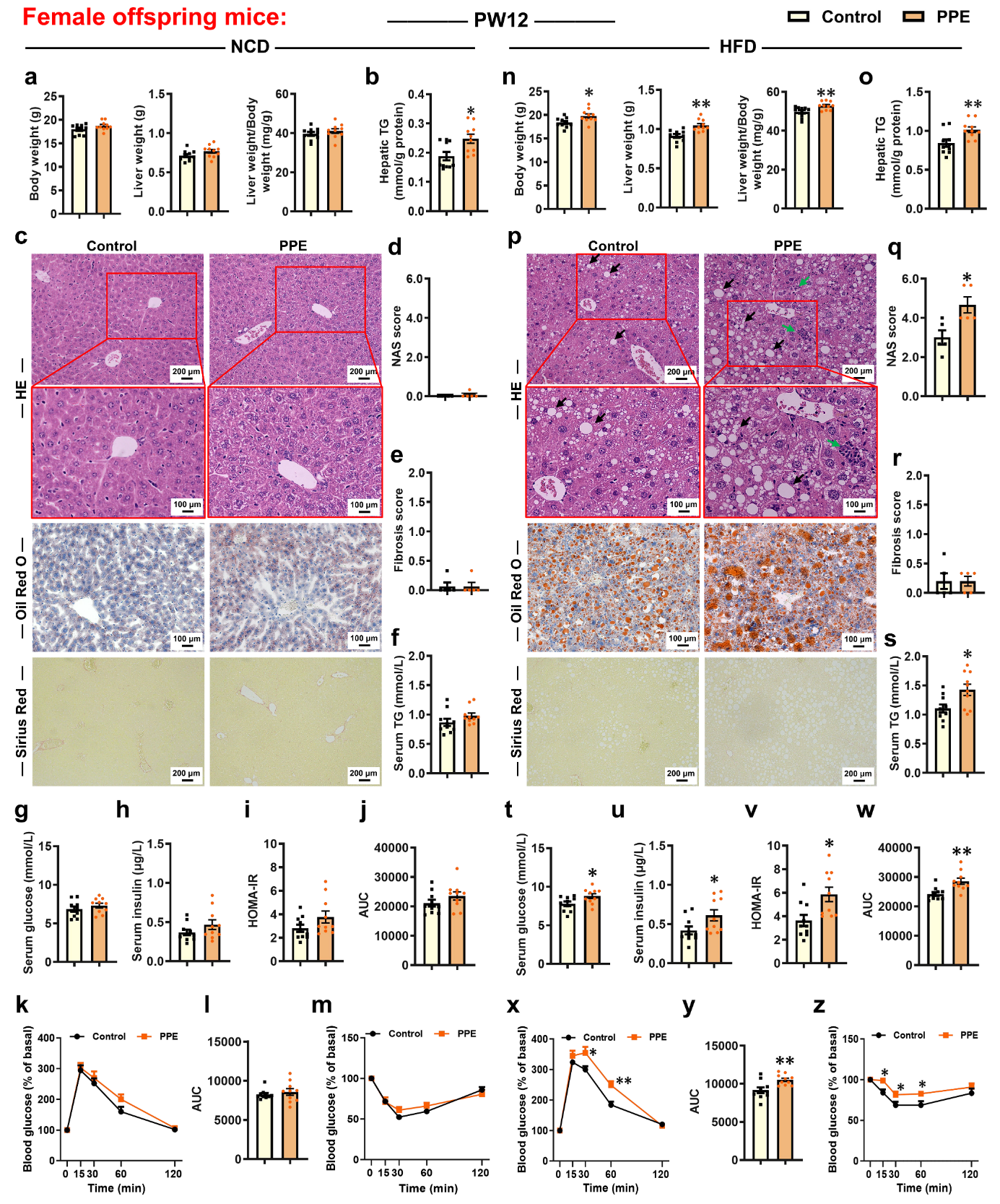
**

**Supplementary Fig. 6 The effect of PPE on** **susceptibility to MASLD in female offspring mice.** Pregnant C57BL/6 mice were intragastrically administrated with prednisone (0.5 mg/kg) or vehicle control (0.5% CMC-Na) per day from GD0-18, and then offspring mice in PW12 were obtained for further analysis. Among these offspring, part of them were fed with HFD (D12492) during PW8-12. (a, n) Body weights, liver weights, liver weight/Body weight; (b, o) Hepatic TG content; (c, p) Representative micrographs of liver sections stained with HE, oil red O and sirius red staining, scale bar: 100 μm and 200 μm; (d, q) Liver NAS score; (e, r) Liver fibrosis score; (f, s) Serum TG levels; (g−i, t−v) Fasting serum glucose, insulin and HOMA-IR index; (j, k, w, x) Normalized blood glucose levels during IPGTT and corresponding AUC; (l, m, y, z) Normalized blood glucose levels during IPITT and corresponding AUC. Mean ± S.E.M., n = 5 for histopathological data, n = 10 for others data. Statistical significance was determined by two-tailed unpaired Student’s *t*-test (a−j, l, n−w, y) and two-way ANOVA for repeated measures followed by Bonferroni’s post-hoc test (k, m, x, z). ^⁎^*P <* 0.05, ^⁎⁎^*P <* 0.01 *vs.* control. AUC, area under the curve; CMC-Na, carboxymethyl cellulose sodium; HE, hematoxylin eosin; HFD, high-fat diet; HOMA-IR, homeostatic model assessment of insulin resistance; IPGTT, intraperitoneal glucose tolerance test; IPITT, intraperitoneal glucose tolerance test; NCD, normal-chow diet; PPE, prenatal prednisone exposure; PW, postnatal week; TG, triglyceride.


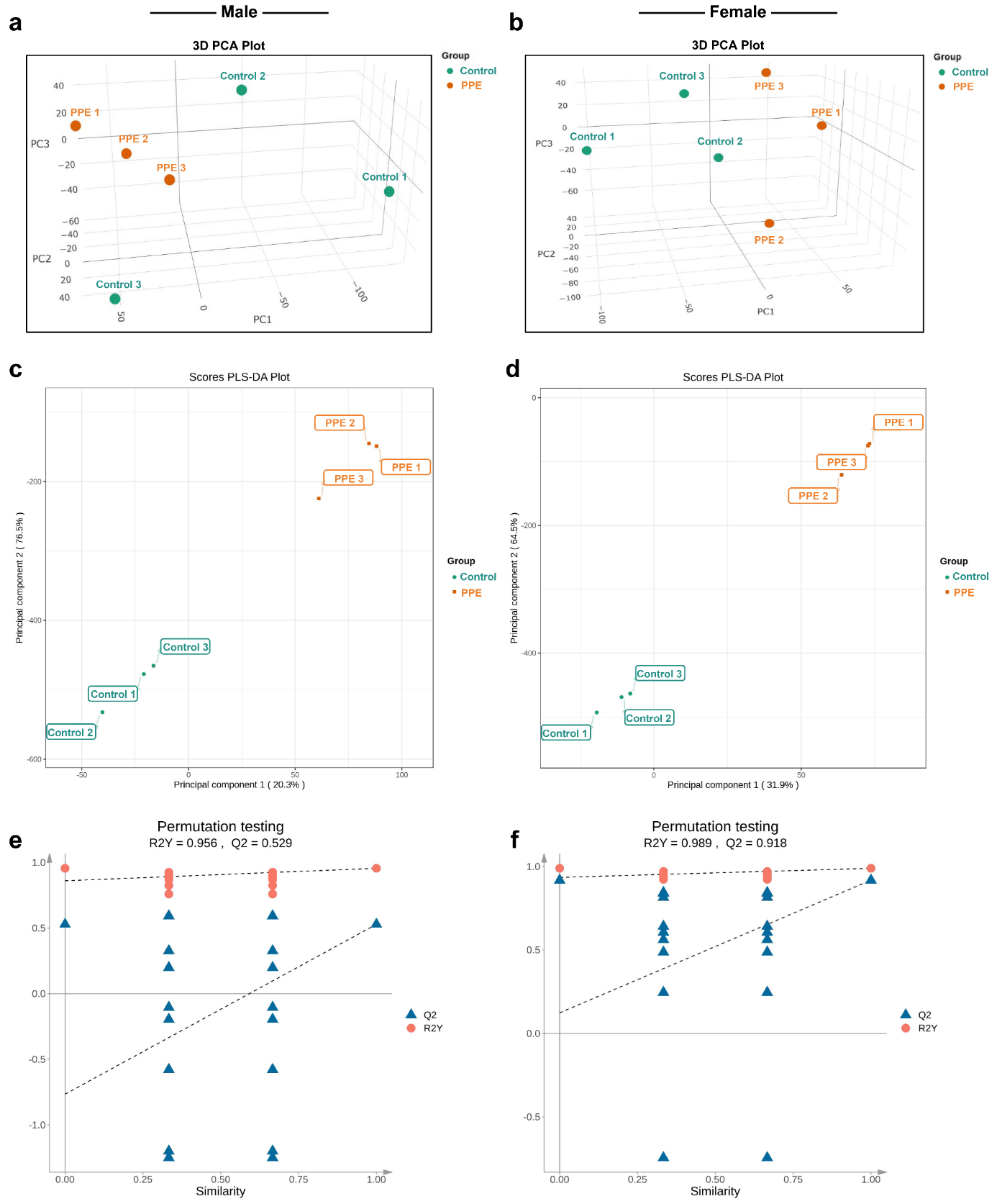


**Supplementary Fig. 7 PCA and PLS-DA score plot of RNA-seq data in male and female fetal liver samples obtained from PPE and control groups.** (a, b) 3D-PCA score plot; (c, d) PLS-DA score plot; (e, f) permutation tests from PLS-DA. PCA, principal component analysis; PLS-DA, partial least-squares discriminant analysis; PPE, prenatal prednisone exposure.


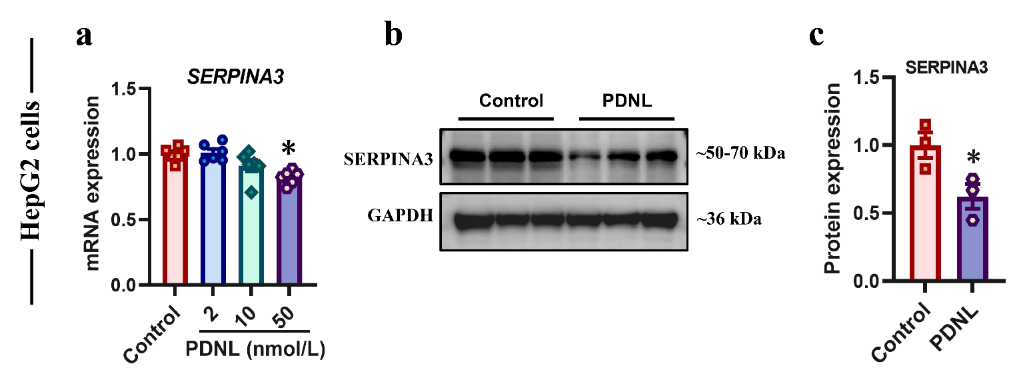


**Supplementary Fig. 8 The effect of** **prednisolone on** **SERPINA3 expression in HepG2 cells.** HepG2 cells were treated with different concentrations (0, 2, 10, 50 nmol/L) of prednisolone for 72 h, and then harvested for further analysis. (a) The mRNA expression of *SERPINA3*; (b, c) Representative WB images and semi-quantitative results of SERPINA3 protein expression. Mean ± S.E.M., n = 6 for RT-qRCR, n = 3 for WB. Statistical significance was determined by one-way ANOVA with Dunnett’s post-hoc test (a) and two-tailed unpaired Student’s *t*-test (b, c). ^*^*P* < 0.05 *vs.* control. GAPDH, glyceraldehyde-3-phosphate dehydrogenase; PDNL, prednisolone; SERPINA3, serpin family A member 3; WB, western blot.

**
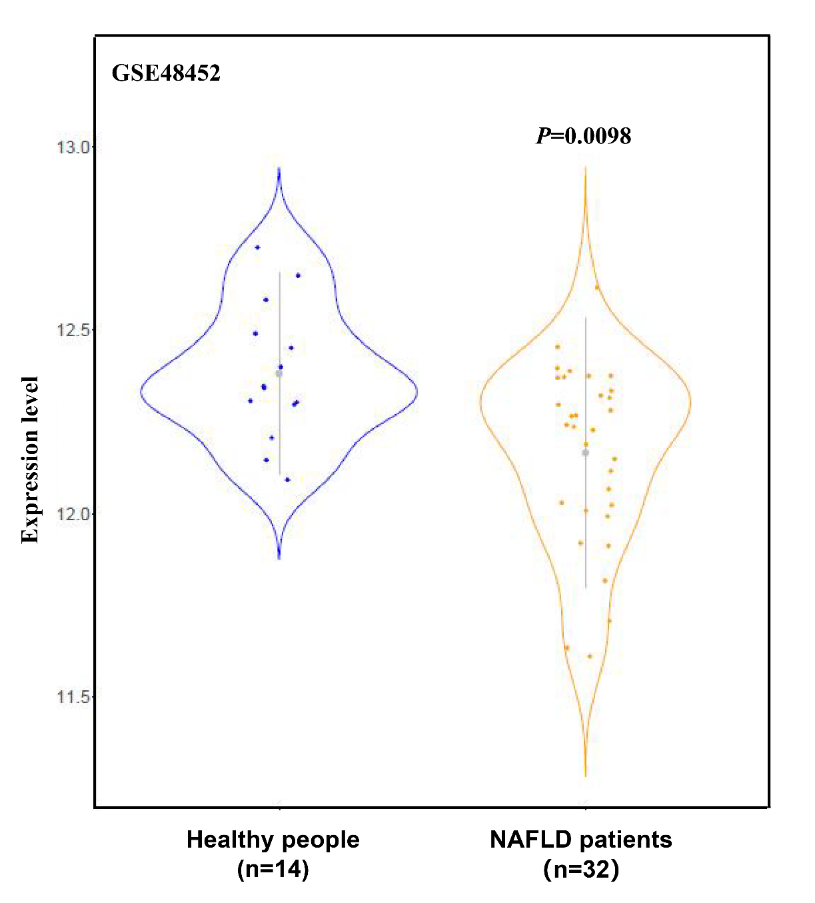
**

**Supplementary Fig. 9 *SERPINA3* expression in patients with NAFLD.** Data analyzed were from the GEO database (GSE48452).


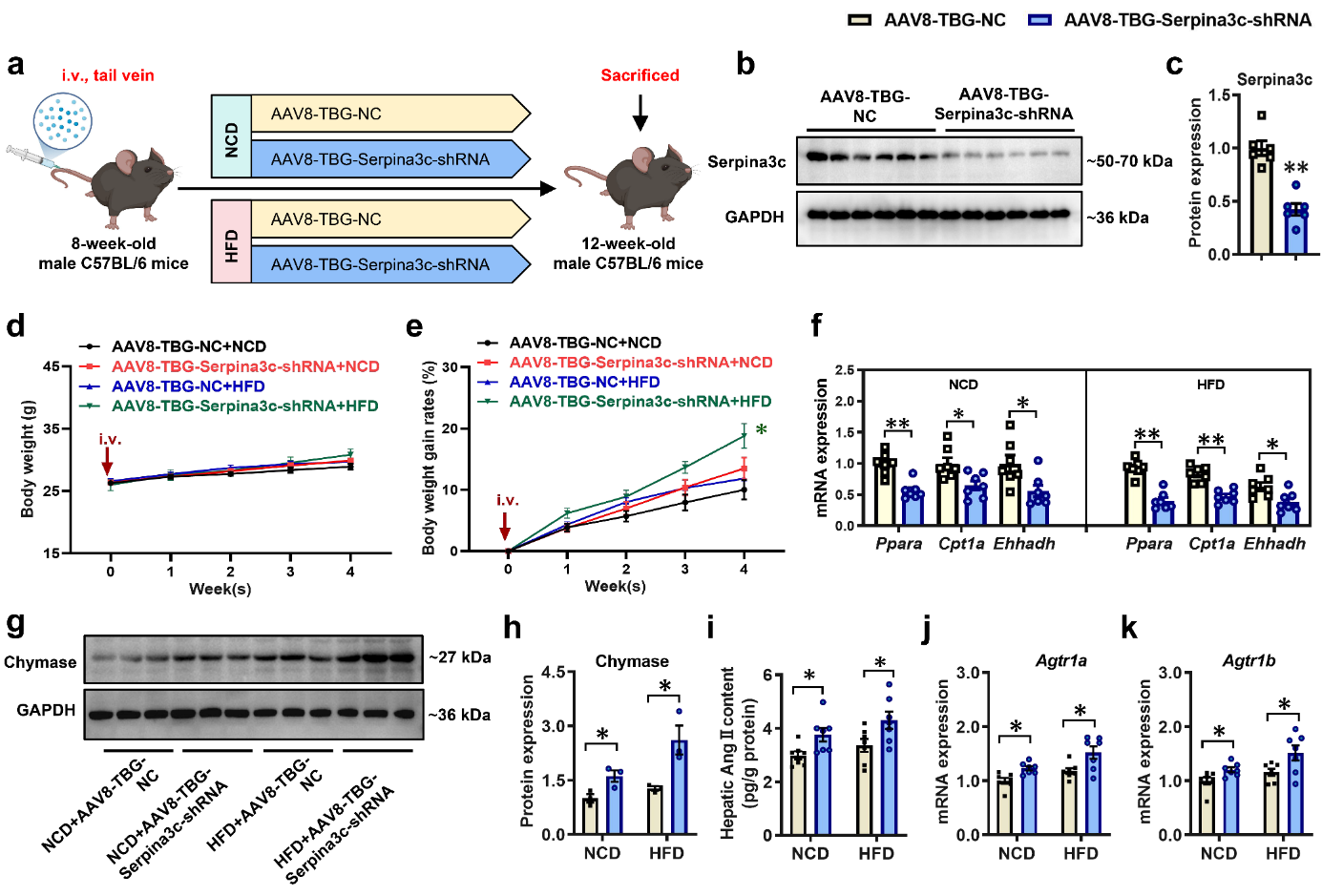


**Supplementary Fig. 10 The effect of liver-specific knockdown of Serpina3c on body weights and gene expression of hepatic lipid metabolism and chymase-Ang II-AT1R axis in male mice.** 8-week-old male C57BL/6 mice were administered with AAV8-TBG-Serpina3c-shRNA *via* tail vein injection for knocking down Serpina3c in mice livers, and samples were obtained at 12 weeks of age for further analysis. Body weight gain rate (%) = (BW_X_ – BW_0_) / BW_0_ × 100. (a) Experimental procedures; (b, c) Representative WB images and semi-quantitative results of hepatic Serpina3c protein expression; (d, e) Body weights and body weight gain rates; (f) The mRNA expression of lipid metabolism-related genes (*Ppara*, *Cpt1a* and *Ehhadh*) in livers; (g, h) Representative WB images and semi-quantitative results of hepatic chymase protein expression; (i) Hepatic Ang II content; (j, k) The mRNA expression of *Agtr1a* and *Agtr1b* in livers. Mean ± S.E.M., n = 6 for WB, n = 7 for other data. Statistical significance was determined by two-tailed unpaired Student’s *t*-test (b, c, f−k), and and two-way ANOVA for repeated measures followed by Bonferroni’s post-hoc test (d, e). **P* < 0.05, ***P* < 0.01 *vs.* AAV8-TBG-NC. AAV8, adeno-associated virus serotype 8; *Cpt1a*, carnitine palmitoyltransferase 1α; *Ehhadh*, enoyl-CoA hydratase and 3-hydroxyacyl CoA dehydrogenase; HFD, high-fat diet; i.v., intravenous injection; NCD, normal-chow diet; *Ppara*, peroxisome proliferators activated receptor α; TBG, thyroxine-binding globulin; WB, western blot.


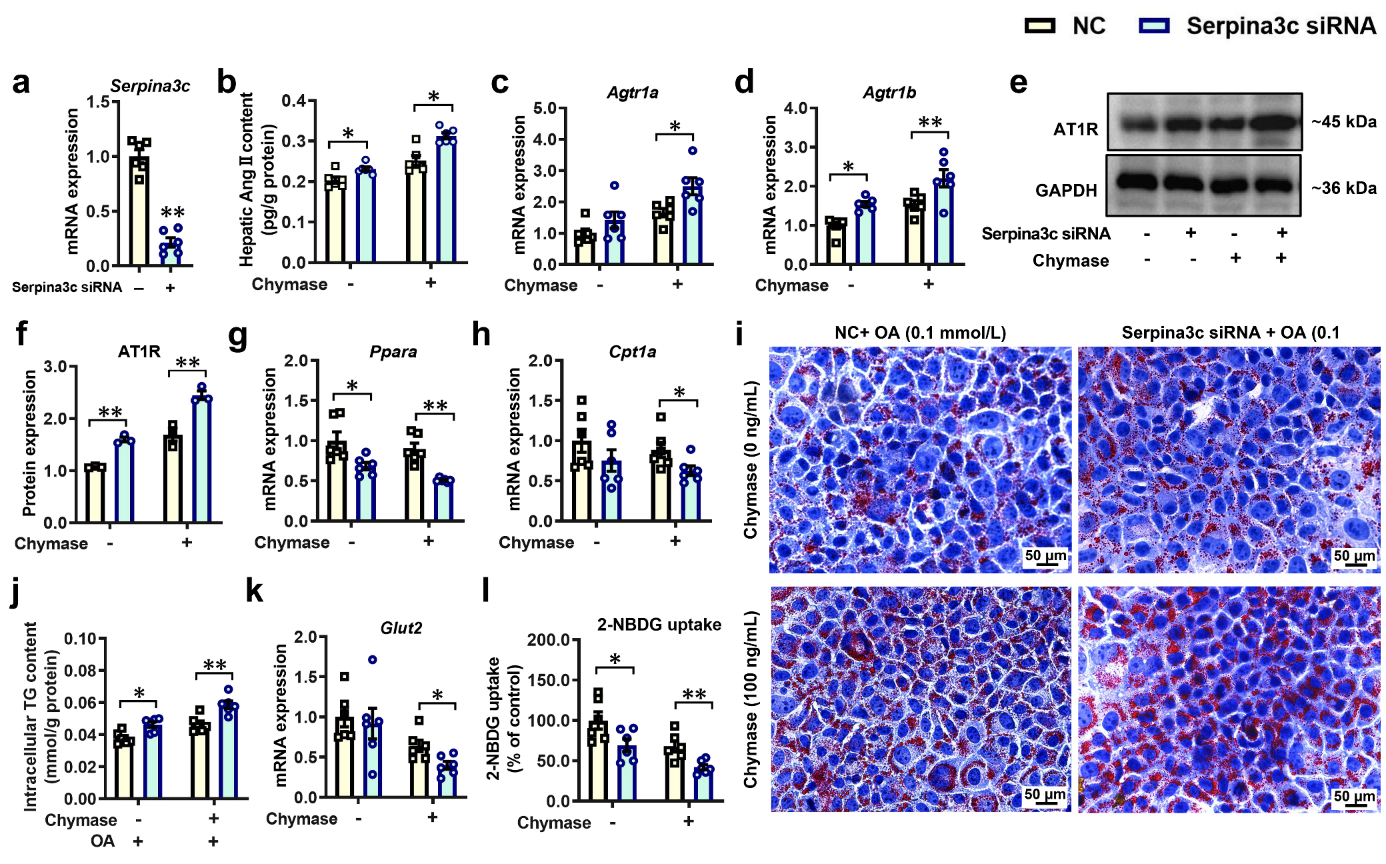


**Supplementary Fig. 11 Effects of knockdown of** **Serpina3c on the chymase-Ang II-AT1R axis as well as glucose and lipid metabolism in AML12 cells.** AML12 cells were transfected with/without Serpina3c siRNA (100 pmol) for 6 h in 6-well plates, and then cultured for 72 h before harvesting the cells for subsequent analysis. Oleic acid (OA, final concentration 0.1 mmol/L) and chymase (final concentration 100 ng/mL) were added 24 h before cell collection. (a) Serpina3c mRNA expression; (b) Intracellular Ang II content; (c, d) *Agtr1a* and *Agtr1b* mRNA expression; (e, f) Representative WB images and semi-quantitative results of AT1R protein expression; (g, h) *Ppara* and *Cpt1a* mRNA expression; (i) Representative micrographs of Oil Red O staining, scale bar: 50 μm; (j) Intracellular TG content; (k) *Glut2* mRNA expression; (l) 2-NBDG uptake. Mean ± S.E.M., n = 3 for WB and Oil Red O staining, n = 6 for other data. Statistical significance was determined by two-tailed unpaired Student’s *t*-test. ^*^*P* < 0.05, ^**^*P* < 0.01 *vs.* NC group. 2-NBDG, 2-Deoxy-2- [(7-nitro-2,1,3-benzoxadiazol-4-yl) amino]-D-glucose; *Agtr1a*, angiotensin II type 1a receptor gene; *Agtr1b*, angiotensin II type 1b receptor gene; Ang II, angiotensin II; AT1R, angiotensinⅡtype 1 receptor; Akt, protein kinase B; *Cpt1a*, carnitine palmitoyltransferase 1α; GAPDH, glyceraldehyde-3-phosphate dehydrogenase; *Glut2*, glucose transporter 2; OA, oleic acid; p-Akt, phospho-Akt; *Ppara*, peroxisome proliferators activated receptor α; Serpina3c, serine (or cysteine) peptidase inhibitor, clade A, member 3C; TG, triglyceride; WB, western blot.


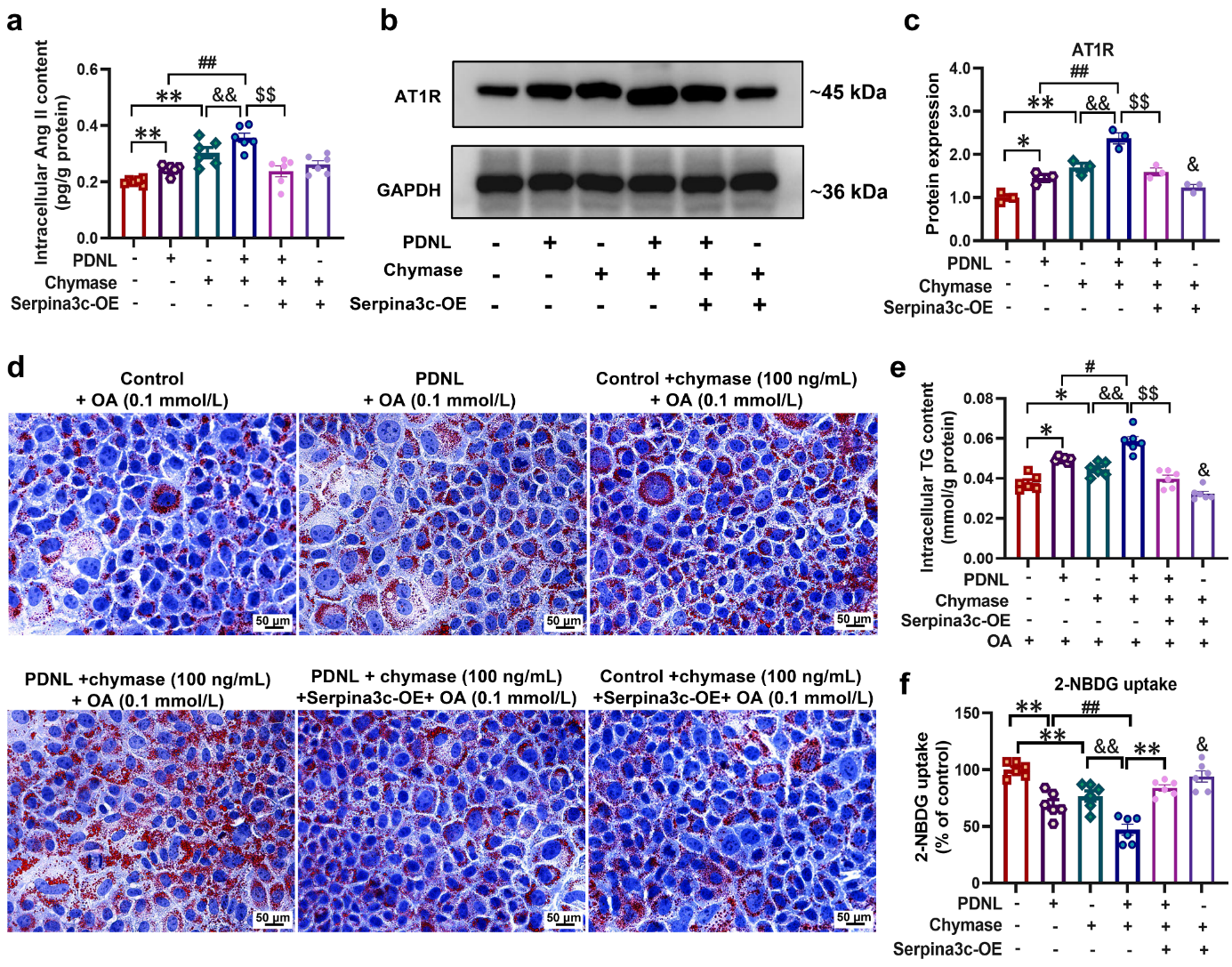


**Supplementary Fig. 12 Overexpression of Serpina3c opposes the stimulatory response to chymase in prednisolone-treated AML12 cells.** AML12 cells were transfected with/without construct Serpina3c overexpression plasmid for 6 h in 6-well plates, and then cultured for 72 h in the presence of prednisolone (0 or 50 nmol/L). Oleic acid (OA, final concentration 0.1 mmol/L) and chymase (final concentration 100 ng/mL) were added 24 h before cell collection. (a) Intracellular Ang II content; (b, c) Representative WB images and semi-quantitative results of AT1R protein expression; (d) Representative micrographs of Oil Red O staining, scale bar: 50 μm; (e) Intracellular TG content; (f) 2-NBDG uptake. Mean ± S.E.M., n = 3 for WB and Oil Red O staining, n = 6 for other data. Statistical significance was determined by one-way ANOVA with Tukey’s post-hoc test. ^*^*P* < 0.05, ^**^*P* < 0.01 *vs.* Control group; ^#^*P* < 0.05, ^##^*P* < 0.01 *vs.* PDNL group; ^&^*P* < 0.05, ^&&^*P* < 0.01 *vs.* Chymase group; ^$^*P* < 0.05, ^$$^*P* < 0.01 *vs.* PDNL + Chymase group. 2-NBDG, 2-Deoxy-2- [(7-nitro-2,1,3-benzoxadiazol-4-yl) amino]-D-glucose; Akt, protein kinase B; Ang II, angiotensin II; AT1R, angiotensin Ⅱ type 1 receptor; GAPDH, glyceraldehyde-3-phosphate dehydrogenase; *Glut2*, glucose transporter 2; OA, oleic acid; OE, Overexpression; p-Akt, phospho-Akt; PDNL, prednisolone; TG, triglyceride; WB, western blot.


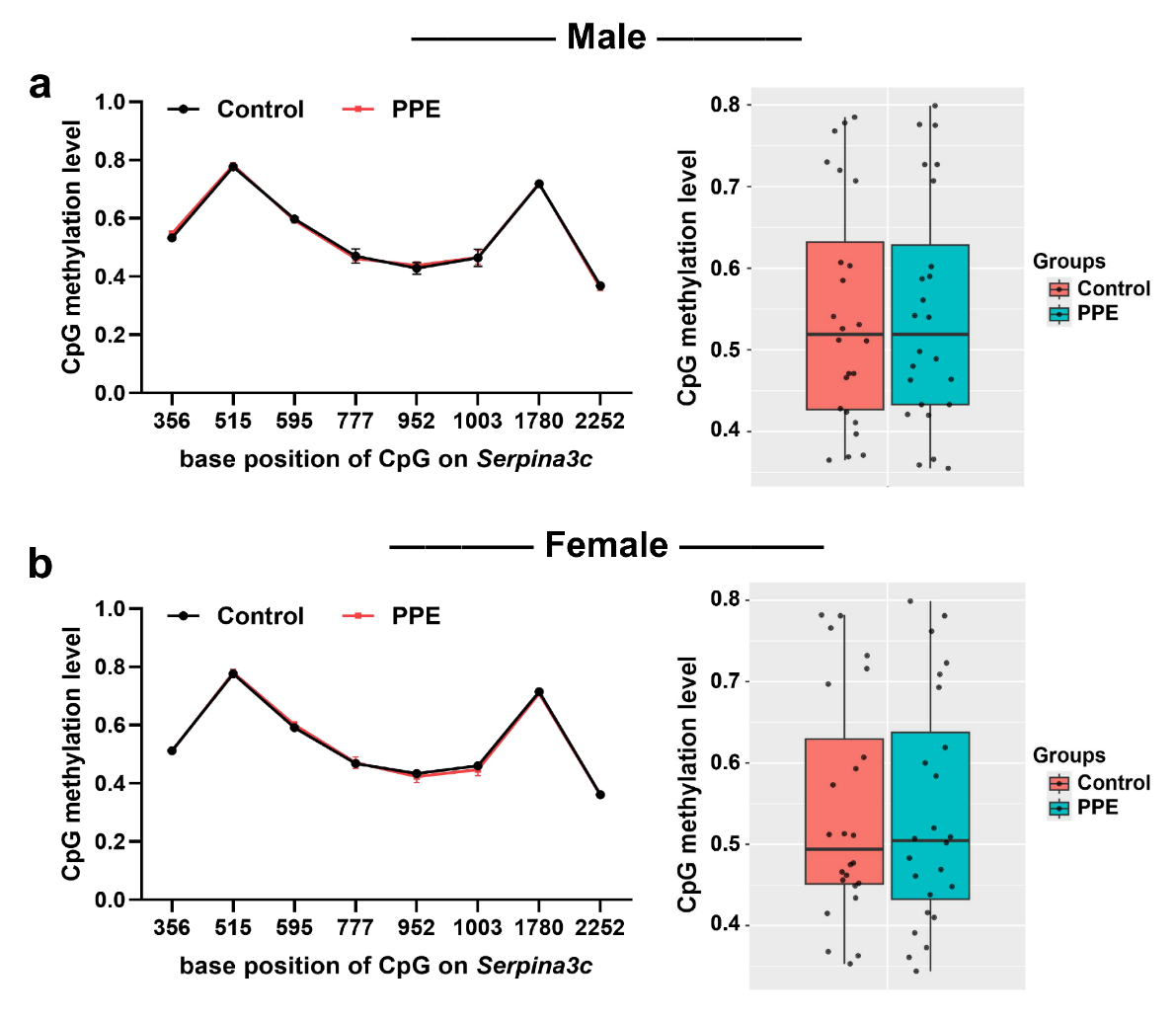


**Supplementary Fig. 13 Effect of PPE on DNA methylation levels in the *Serpina3c* promoter region in both male and female fetal rat liver tissues.** Pregnant Wistar rats were intragastrically administrated with prednisone (0.25 mg/kg) or vehicle control (0.5% CMC-Na) per day from GD0-20, and then fetal rats on GD20 were obtained for further analysis. Mean ± S.E.M., n = 3. Statistical significance was determined by two-tailed unpaired Student’s *t*-test. CMC-Na, Sodium carboxymethyl cellulose; GD, gestational day; PPE, prenatal prednisone exposure.


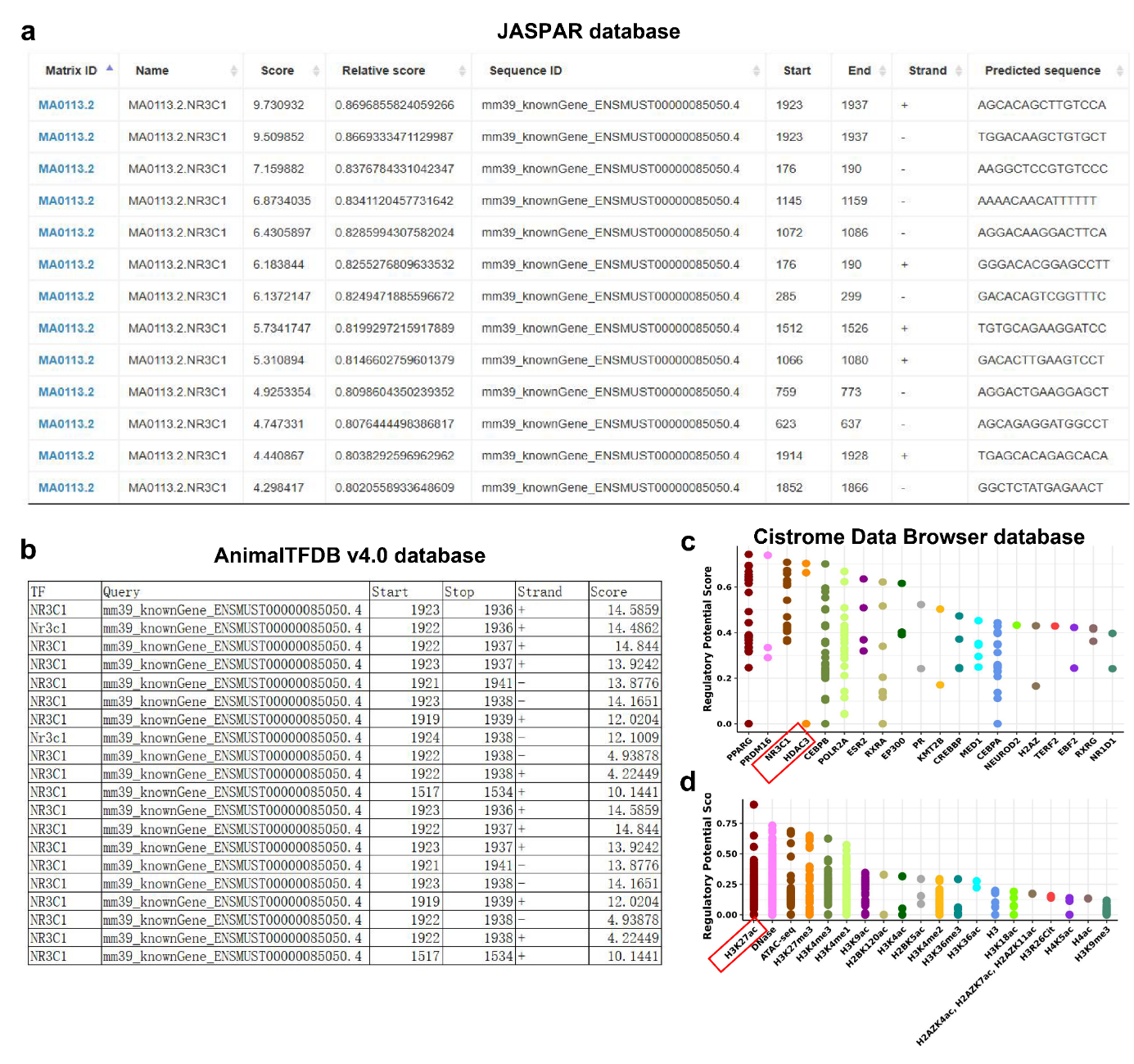


**Supplementary Fig. 14 Database prediction of GR binding and common histone modification forms in the *Serpina3c* gene promoter region.** (a) Prediction of *Serpina3c* promoter region and GR binding site by JASPAR database (https://jaspar.elixir.no/); (b) Prediction of *Serpina3c* promoter region and GR binding site by AnimalTFDB v4.0 database (http://bioinfo.life.hust.edu.cn/AnimalTFDB4/); (c) Prediction of transcription factors and chromatin regulators in the *Serpina3c* promoter region by the Cistrome Data Browser database (http://cistrome.org/db/#/); (d) Prediction of common histone modification forms in the *Serpina3c* promoter region by the Cistrome Data Browser database (http://cistrome.org/db/#/).


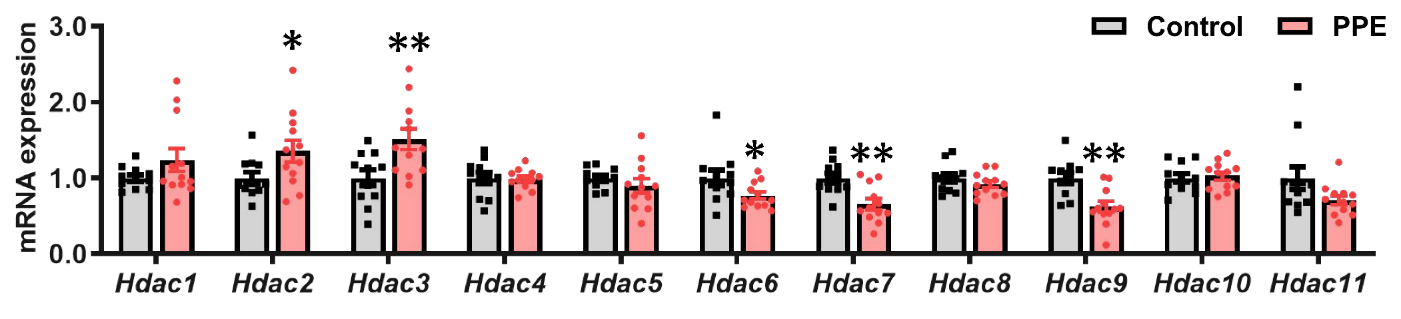


**Supplementary Fig. 15 Effect of PPE on the *Hdacs* expression in livers of male fetal rats.** Pregnant Wistar rats were intragastrically administrated with prednisone (0.25 mg/kg) or vehicle control (0.5% CMC-Na) per day from GD0-20, and then fetal rats on GD20 were obtained for further analysis. Mean ± S.E.M., n = 11-12. Statistical significance was determined by two-tailed unpaired Student’s *t*-test. ^*^*P* < 0.05, ^**^*P* < 0.01 *vs.* control group. CMC-Na, Sodium carboxymethyl cellulose; GD, gestational day; *Hdacs*, histone deacetylases; PPE, prenatal prednisone exposure.


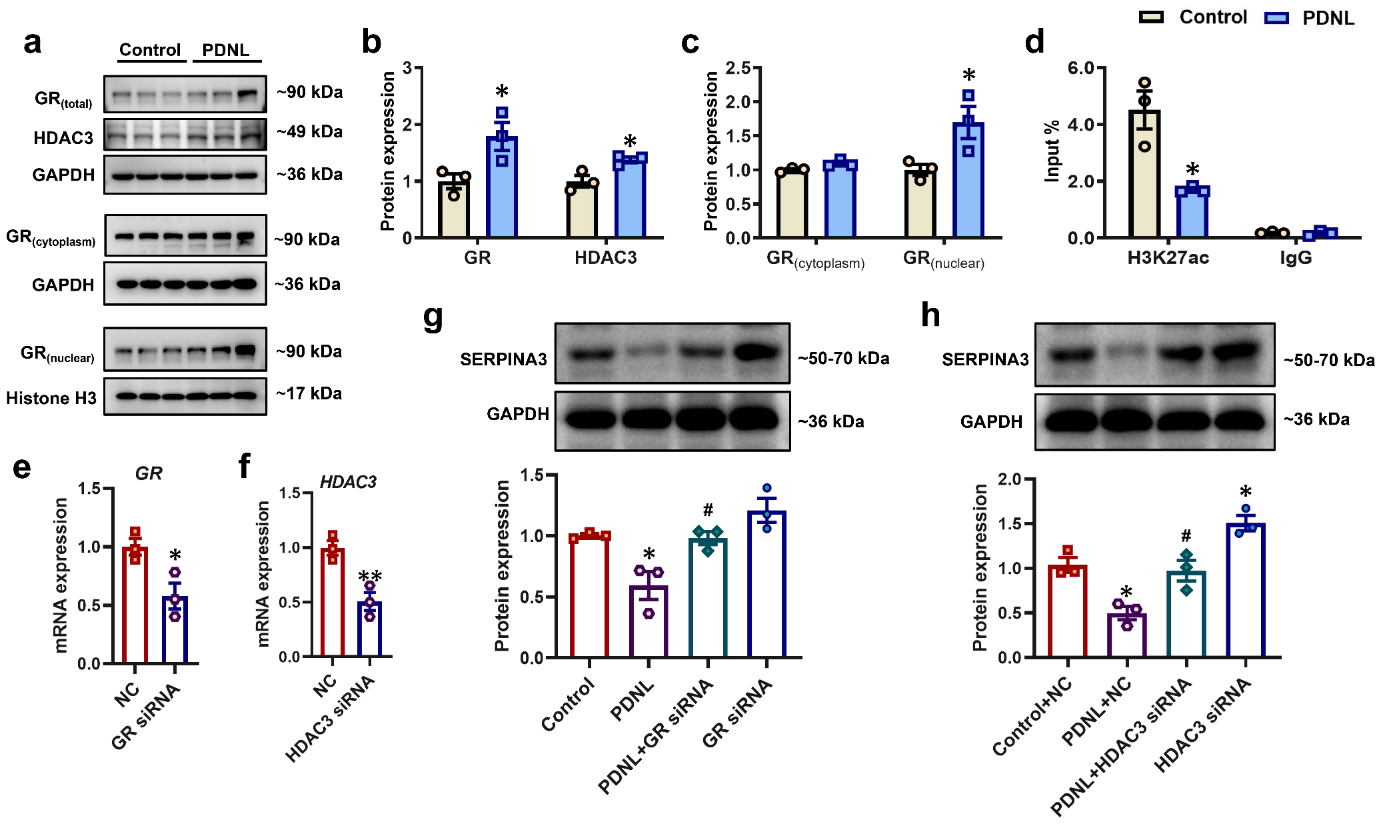


**Supplementary Fig. 16 Effect of prednisolone on GR-HDAC3 signaling and histone acetylation levels of the** **SERPINA3 gene promoter region in HepG2 cells.** HepG2 cells were transfected with/without GR siRNA (100 pmol) or HDAC3 siRNA (100 pmol) for 6 h in 6-well plates and then cultured for 72 h in the presence of prednisolone (0 or 50 nmol/L). (a−c) Representative WB images and semi-quantitative results of GR and HDAC3 protein expression; (d) H3K27ac levels in the *Serpina3c* gene promoter region; (e) *GR(/NR3C1)* mRNA expression; (f) *HDAC3* mRNA expression; (g, h) Representative WB images and semi-quantitative results of SERPINA3 protein expression. Mean ± S.E.M., n = 3. Statistical significance was determined by two-tailed unpaired Student’s *t*-test (a−f), and one-way ANOVA with Tukey’s post-hoc test (g, h). ^*^*P* < 0.05, ^**^*P* < 0.01 *vs.* control; ^#^*P <* 0.05, ^##^*P <* 0.01 *vs.* prednisolone-treated group. GAPDH, glyceraldehyde-3-phosphate dehydrogenase; GR, Glucocorticoid receptor; HDAC3, histone deacetylase3; PDNL, prednisolone.

**Supplementary Tables**

**Supplementary Table 1 The information of main chemicals and reagents used in this study**

| **Chemicals and reagents** | **Source** |
| --- | --- |
| Isoflurane (CAS. No. 26675-46-7, Cat. No. R510-22) | RWD Life Science Co., Ltd. (Shenzhen, China) |
| Sodium carboxymethyl cellulose (CMC-Na; CAS. No. 9004-32-4, Cat. No. 30036365) | Sinopharm Chemical Reagent Co., Ltd. (Shanghai, China) |
| Mifepristone (RU486; CAS. No. 84371-65-3, Cat. No. 475838, ≥98%) | Sigma-Aldrich Corp. (St. Louis, MO, USA) |
| Trichostatin A (TSA; CAS. No. 58880-19-6, Cat. No. 647925, ≥98%) | Sigma-Aldrich Corp. (St. Louis, MO, USA) |
| Methyl sulfoxide (DMSO; CAS. No. 67-68-5, Cat. No. D2650, ≥99.7%) | Sigma-Aldrich Corp. (St. Louis, MO, USA) |
| 2-Deoxy-2- [(7-nitro-2,1,3-benzoxadiazol-4-yl) amino]-D-glucose (2-NBDG; CAS No.186689-07-6, Cat. No. SML4177, ≥97%) | Sigma-Aldrich Corp. (St. Louis, MO, USA) |
| Triton X-100 (CAS. No. 9036-19-5, Cat. No. 93443) | Sigma-Aldrich Corp. (St. Louis, MO, USA) |
| Sodium oleate (CAS. No. 143-19-1, Cat. No. S817542, ≥98%) | Shanghai Macklin Biochemical Technology Co., Ltd. (Shanghai, China) |
| Recombinant Human Chymase (CMA1, Cat. No. CSB-EP005599HU) | Wuhan Huamei Bioengineering Co., Ltd. (Wuhan, Hubei, China) |
| Dexamethasone (CAS. No. 50-02-2, Cat. No. MB1434, ≥99%) | Dalian Meilun Biotechnology Co., Ltd. (Dalian, Liaoning, China) |
| Penicillin/Streptomycin,sterile (100×) (Cat. No. MA0110) | Dalian Meilun Biotechnology Co., Ltd. (Dalian, Liaoning, China) |
| Gibco™ Fetal Bovine Serum (FBS; Cat. No. A5670701) | Thermo Fisher Scientific Inc. (Waltham MA, USA) |
| Gibco™ Dulbecco's Modified Eagle Medium (DMEM; Cat. No. 11995065) | Thermo Fisher Scientific Inc. (Waltham MA, USA) |
| Gibco™ DMEM /Nutrient Mixture F-12 (DMEM/F-12; Cat. No. 11320033) | Thermo Fisher Scientific Inc. (Waltham MA, USA) |
| Gibco™ Trypsin-EDTA (0.25%) (Cat. No. 25200072) | Thermo Fisher Scientific Inc. (Waltham MA, USA) |
| Invitrogen™ Lipofectamine 3000 Transfection Reagent (Cat. No. L3000015) | Thermo Fisher Scientific Inc. (Waltham MA, USA) |
| TRIzol Reagent (Cat.No. 15596026) | Thermo Fisher Scientific Inc. (Waltham MA, USA) |
| HiScript III RT SuperMix for qPCR (+gDNA wiper) (Cat. No. R323-01) | Vazyme Biotech Co., Ltd. (Nanjing, Jiangsu, China) |
| Taq Pro Universal SYBR qPCR Master Mix (Cat. No. Q712-02) | Vazyme Biotech Co., Ltd. (Nanjing, Jiangsu, China) |
| 180 kDa and 250 kDa Plus Prestained Protein Marker (Cat. No. MP202-01; MP202-01) | Vazyme Biotech Co., Ltd. (Nanjing, Jiangsu, China) |
| Prestained Protein Marker (11-180kDa) (Cat. No. MKP008) | Shanghai Wansheng Haotian Biotechnology Co., Ltd. (Shanghai, China) |
| NcmColor Prestained Colored Protein Marker (Cat. No. P9008) | New Cell & Molecular Biotech Co., Ltd. (Suzhou, Jiangsu, China) |
| Triglyceride (TG) assay kit (Cat. No. A110-1-1) | Nanjing Jiancheng Bioengineering Institute (Nanjing, Jiangsu, China) |
| Glucose assay kit (Cat. No. F006-1-1) | Nanjing Jiancheng Bioengineering Institute (Nanjing, Jiangsu, China) |
| Ultrasensitive Rat Insulin ELISA kit (Cat. No. 10-1251-01) | Mercodia (Uppsala, Sweden) |
| Ultrasensitive Mouse Insulin ELISA kit (Cat. No. 10-1249-01) | Mercodia (Uppsala, Sweden) |
| Hematoxylin-Eosin (HE) Stain Kit (Cat. No. G1120) | Beijing solarbio science﹠technology co., Ltd. (Beijing, China) |
| Rat Angiotensin Ⅱ (Ang Ⅱ) ELISA Kit (Cat. No. JYM0668Ra) | Wuhan JYMBio Technology Co., Ltd. (Wuhan, Hubei, China) |
| Mouse Angiotensin Ⅱ (Ang Ⅱ) ELISA Kit (Cat. No. JYM0080Mo) | Wuhan JYMBio Technology Co., Ltd. (Wuhan, Hubei, China) |
| Rat Alpha-1-Antichymotrypsin (a1ACT) ELISA Kit (Cat. No. RA22998) | Bioswamp® Life Science Lab (Wuhan, Hubei, China) |
| Oil Red O Saturated Solution (Cat. No. G1015) | Wuhan Servicebio Technology Co., Ltd. (Wuhan, Hubei, China) |
| Hematoxylin Stain Solution (Cat. No. G1004) | Wuhan Servicebio Technology Co., Ltd. (Wuhan, Hubei, China) |
| Sirius Red Stain Solution (Cat. No. G1018) | Wuhan Servicebio Technology Co., Ltd. (Wuhan, Hubei, China) |
| Tris Buffered Saline (TBS, Powder; Cat. No. G0001) | Wuhan Servicebio Technology Co., Ltd. (Wuhan, Hubei, China) |
| Tween 20 (Cat. No. GC204002) | Wuhan Servicebio Technology Co., Ltd. (Wuhan, China) |
| RIPA lysis buffer (Cat. No. P0013B) | Beyotime Biotechnology (Shanghai, China) |
| Cell Complete Lysis Buffer for Western and IP (Cat. No. P0037) | Beyotime Biotechnology (Shanghai, China) |
| Enhanced bicinchoninic acid (BCA) protein assay kit (Cat. No. P0010S) | Beyotime Biotechnology (Shanghai, China) |
| Protease inhibitor cocktail for general use, 100× (Cat. No. P1005) | Beyotime Biotechnology (Shanghai, China) |
| Phosphatase inhibitor cocktail A, 50× (Cat. No. P1081) | Beyotime Biotechnology (Shanghai, China) |
| SDS-PAGE sample loading buffer, 5× (Cat. No. P0015) | Beyotime Biotechnology (Shanghai, China) |
| Insulin-Transferrin-Selenium (ITS) Media Supplement (100×) (Cat. No. C0341) | Beyotime Biotechnology (Shanghai, China) |
|  |  |
| Omni-Easy™ one-step color PAGE gel rapid preparation kit (Cat. No. PG210, PG212, PG213, PG214) | Shanghai Epizyme Biomedical Technology Co., Ltd. (Shanghai, China) |
| Primary antibody dilution buffer for western blot (Cat. No. PS114) | Shanghai Epizyme Biomedical Technology Co., Ltd. (Shanghai, China) |
| Protein free rapid blocking buffer (1×) (Cat. No. PS108P) | Shanghai Epizyme Biomedical Technology Co., Ltd. (Shanghai, China) |
| Western blot fast stripping buffer (Cat. No. PS107) | Shanghai Epizyme Biomedical Technology Co., Ltd. (Shanghai, China) |
| TIANguick Midi Purification Kit (Cat. No. DP210831) | TIANGEN Biotech (Beijing) Co., Ltd. (Beijing, China) |
| Enhanced Chemiluminescence (ECL) kit (Cat. No. D046) | Bridgen (Beijing, China) |
| rProtein A/G Beads 4FF (Cat. No. SA032025) | smart-Lifesciences Biotechnology Co.,Ltd. (Changzhou, Jiangsu, China) |
| Dual-Luciferase^®^ Reporter (DLR™) Assay System (Cat. No. E1910) | Promega (Beijing) Biotech Co., Ltd. (Beijing, China) |
| Primers for RT-qPCR and ChIP-qPCR | Wuhan Tianyi Huayu Gene Technology Co., Ltd (Wuhan, Hubei, China) |
| pAAV8-TBG-EGFP-P2A-Serpina3c-3xFLAG-tWPA | Obio Technology (Shanghai) Corp., Ltd (Shanghai, China) |
| pAAV8-TBG-EGFP-tWPA | Obio Technology (Shanghai) Corp., Ltd (Shanghai, China) |
| pcDNA3.1(+)-mouse-Serpina3c | Obio Technology (Shanghai) Corp., Ltd (Shanghai, China) |
| AAV8-GP-1-Serpina3c-mus-227 | Shanghai GenePharma Co.,Ltd (Shanghai, China) |
| AAV8-GP-1-SJ-NC | Shanghai GenePharma Co.,Ltd (Shanghai, China) |
| SiRNA (Mouse-Serpina3c, Human-NR3C1, Human-HDAC3 and Control) | Shanghai GenePharma Co.,Ltd (Shanghai, China) |
| pcDNA3.1(+)-mouse-GR(/NR3C1) | Shanghai GenePharma Co.,Ltd (Shanghai, China) |
| pcDNA3.1(+)-NC | Shanghai GenePharma Co.,Ltd (Shanghai, China) |
| pGL4-basic | Shanghai GenePharma Co.,Ltd (Shanghai, China) |
| pGL4-mouse-Serpina3c-promoter | Shanghai GenePharma Co.,Ltd (Shanghai, China) |
| pRL-TK | Shanghai GenePharma Co.,Ltd (Shanghai, China) |
| DAPI Staining Solution (Cat. No. RM02978) | ABclonal Biotechnology Co., Ltd. (Wuhan, Hubei, China) |
| Pan-Akt Rabbit mAb (Cat. No. A18675) | ABclonal Biotechnology Co., Ltd. (Wuhan, Hubei, China) |
| Phospho-Akt-S473 Rabbit pAb (Cat. No. AP1068) | ABclonal Biotechnology Co., Ltd. (Wuhan, Hubei, China) |
| HRP Goat Anti-Rabbit IgG (H + L) (Cat. No. AS014) | ABclonal Biotechnology Co., Ltd. (Wuhan, Hubei, China) |
| GAPDH Mouse mAb (Cat. No. AC002) | ABclonal Biotechnology Co., Ltd. (Wuhan, Hubei, China) |
| HRP Goat Anti-Mouse IgG (H + L) (Cat. No. AS003) | ABclonal Biotechnology Co., Ltd. (Wuhan, Hubei, China) |
| CPT1A Rabbit mAb (Cat. No. A5307) | ABclonal Biotechnology Co., Ltd. (Wuhan, Hubei, China) |
| PPARα Rabbit mAb (Cat. No. A25296) | ABclonal Biotechnology Co., Ltd. (Wuhan, Hubei, China) |
| Mast Cell Chymase (CMA1) Rabbit mAb (Cat. No. A11480) | ABclonal Biotechnology Co., Ltd. (Wuhan, Hubei, China) |
| Alpha-1 Antichymotrypsin (SERPINA3) Rabbit pAb (Cat. No. A2803) | ABclonal Biotechnology Co., Ltd. (Wuhan, Hubei, China) |
| Acetyl-Histone H3-K9 Rabbit pAb (Cat. No. A7255) | ABclonal Biotechnology Co., Ltd. (Wuhan, Hubei, China) |
| Histone H3 Rabbit pAb (Cat. No. A2348) | ABclonal Biotechnology Co., Ltd. (Wuhan, Hubei, China) |
| Acetyl-Histone H3-K14 Rabbit pAb (Cat. No. A7254) | ABclonal Biotechnology Co., Ltd. (Wuhan, Hubei, China) |
| Rabbit Control IgG (Cat. No. AC005) | ABclonal Biotechnology Co., Ltd. (Wuhan, Hubei, China) |
| Acetyl-Histone H3-K27 Rabbit pAb (Cat. No. A7253) | ABclonal Biotechnology Co., Ltd. (Wuhan, Hubei, China) |
| Mouse Control IgG (Cat. No. AC011) | ABclonal Biotechnology Co., Ltd. (Wuhan, Hubei, China) |
| ABflo^®^ 594-conjugated Goat Anti-Rabbit IgG (H + L) (Cat. No. AS039) | ABclonal Biotechnology Co., Ltd. (Wuhan, Hubei, China) |
| ABflo^®^ 488-conjugated Goat Anti-Rabbit IgG (H + L) (Cat. No. AS073) | ABclonal Biotechnology Co., Ltd. (Wuhan, Hubei, China) |
| ABflo^®^ 594-conjugated Goat Anti-Mouse IgG (H + L) (Cat. No. AS054) | ABclonal Biotechnology Co., Ltd. (Wuhan, Hubei, China) |
| ABflo^®^ 488-conjugated Goat Anti-Mouse IgG (H + L) (Cat. No. AS076) | ABclonal Biotechnology Co., Ltd. (Wuhan, Hubei, China) |
| Mouse monoclonal PPARα antibody (Cat. No. sc-398394) | Santa Cruz Biotechnology (California, USA) |
| Mouse monoclonal Glut2 antibody (Cat. No. sc-518022) | Santa Cruz Biotechnology (California, USA) |
| Histone Deacetylase 3 (HDAC3) Antibody (Cat. No. sc-376957) | Santa Cruz Biotechnology (California, USA) |
| Anti-SerpinA3c Antibody, Rabbit Polyclonal (Cat. No. 50375-RP02) | Sino Biological Inc. (Beijing, China) |
| Anti-Cathepsin G antibody [EPR24170-169] (Cat. No. ab282105) | Abcam Plc. (Cambridge, UK) |
| Anti-Glucocorticoid Receptor antibody [EPR19621] (Cat. No. ab183127) | Abcam Plc. (Cambridge, UK) |
| Anti-Angiotensin II Type 1 Receptor antibody [1E10-1A9] (Cat. No. ab9391) | Abcam Plc. (Cambridge, UK) |

**Supplementary Table 2 List of siRNA sequences**

| **Name** | **Sense sequence (5’→3’)** | **Antisense sequence (5’→3’)** |
| --- | --- | --- |
| **Mouse-Serpina3c siRNA** | ACACAACUCGACAGUCUCA TT | UGAGACUGUCGAGUUGUGUCC |
| **Mouse-Control siRNA** | UUCUCCGAACGUGUCACGUTT | ACGUGACACGUUCGGAGAATT |
| **Human-NR3C1 siRNA** | GGAGAUGACAACUUGACUUTT | AAGUCAAGUUGUCAUCUCCTT |
| **Human-HDAC3 siRNA** | GGUA GUGGACUUCUACCAATT | UUGGUAGAAGUCCACUACCTG |
| **Human-Control siRNA** | UUCUCCGAACGUGUCACGUTT | ACGUGACACGUU CGGAGAATT |

**Supplementary Table 3 Nonalcoholic fatty liver disease (NAFLD) activity score (NAS) and liver fibrosis scores as per the nonalcoholic steatohepatitis Clinical Research Network (NASH-CRN) System.**

| **Histologic Feature** | **Score** | **Definition** |
| --- | --- | --- |
| **Steatosis** | 0 | < 5% |
|  | 1 | 5%–33% |
|  | 2 | 34%–66% |
|  | 3 | >66% |
| **+** |  |  |
| **Hepatocyte ballooning** | 0 | None |
|  | 1 | Few |
|  | 2 | Many |
| **+** |  |  |
| **Lobular Inflammation** | 0 | None |
|  | 1 | < 2 foci per 20× field |
|  | 2 | 2–4 foci per 20× field |
|  | 3 | > 4 foci per 20× field |
| **= NAFLD activity score (NAS); range 0–8** | | |
| **Fibrosis** | 0 | No fibrosis |
|  | 1 |  |
|  | 1a | Zone 3 mild perisinusoidal fibrosis |
|  | 1b | Zone 3 moderate perisinusoidal fibrosis |
|  | 1c | Portal or portal fibrosis only |
|  | 2 | Zone 3 + portal/periportal fibrosis |
|  | 3 | Bridging fibrosis |
|  | 4 | Cirrhosis |
| **= Fibrosis score; range 0**–**4** | | |

**Supplementary Table 4 The primers sequence of serpina3c promoter DNA methylation sequencing**

| **Name** | **Primers sequence (5’→3’)** | **Start** | **End** |
| --- | --- | --- | --- |
| **Primer 1** | Forward primer: ATTTGAAGGGTAAATAGGGAATTGGA | 191 | 216 |
|  | Reverse primer: CCAACAATCTCTTTACTCTAAAAATAATTTTAAC | 381 | 414 |
| **Primer 2** | Forward primer: TTTTTGTTGAGGGYGAAATTTTTGAGTG | 463 | 490 |
|  | Reverse primer: CTATCATAACAAAAATAACACAAAACTATTACC | 596 | 628 |
| **Primer 3** | Forward primer: TAAAATTTTTTAGTAGAGTTAGAGGAGTAG | 652 | 681 |
|  | Reverse primer: TTCCCAAACCTATATAATACTAACTATAAAC | 839 | 869 |
| **Primer 4** | Forward primer: GAGGTAGGAATTTTGGGTATTATTTG | 887 | 912 |
|  | Reverse primer: CAAAACACAAATTATCCAAAACAATATTTCC | 1045 | 1075 |
| **Primer 5** | Forward primer: TTTATAGGATTTGAAAGAGTAATTTTTAATTTTATATGG | 1629 | 1667 |
|  | Reverse primer: ATCAACATACCTATCTTCATACCAAC | 1781 | 1806 |
| **Primer 6** | Forward primer: TGAGGATATAATAGTAAAGTATTAGTAAATATATTTTTG | 2200 | 2238 |
|  | Reverse primer: TTACTAACAACATATAAATCAAAAAACCATCAC | 2322 | 2354 |

**Supplementary Table 5 List of primers sequences used for RT-qPCR.**

| **Genes** | **Forward primer sequence (5’→3’)** | **Reverse primer sequence (5’→3’)** |
| --- | --- | --- |
| **Rat** |  |  |
| *Gapdh* | GGGTGTGAACCACGAGAAAT | ACTGTGGTCATGAGCCCTTC |
| *Srebp1* | CGCCCATCGGTTTAAGGACT | ACACTCGTTTCTTTCGGGCT |
| *Fasn* | TGGCTCAGCATGGCCGCTTC | CAGCTGTCGTTGGCCCCCTC |
| *Cd36* | CCAGAACCCAGACAACCACT | CACAGGCTTTCCTTCTTTGC |
| *Mttp* | AGATGTGCAACGTGGTATTC | CCTGACTAGGCTCGACTTTA |
| *Ppara* | GCTGAAGTACGGTGTGTATG | TAGGAACTCTCGGGTGATG |
| *Cpt1a* | CCTTTCCTGAAGGAGGTATTG | AGGACACATAGTCAGGGTT |
| *Ehhadh* | TGGTTGGCAACTGCTATG | GCTTGCTACCTTCCTCTAAC |
| *Cyp4a8* | GAGGATCTACCCACCTGTAA | AGGAGAGCATGACTGTGATA |
| *Glut2* | ACATCCTACTTGGCCTATCT | GACTTCCTCTTCCAACTTTAG |
| *Gck* | GGGACTTTGAGATGGATGTG | CATTGGCGGTCTTCATAGTAG |
| *Pck1* | CCCGAAGGCAAGAAGAAATA | GTCATCACCCACACATTCA |
| *G6pc* | GCAGGTGTATACTACGTTATGG | CAGTATCCCAACCACAAGAC |
| *Serpina3c* | CATGGTGCTGGTGAACTATC | TCACAGACCTCTTCTCATCC |
| *Agtr1a* | GTGGCCAGTGTCTTTCTT | CCGAAGCGATCTTACATAGG |
| *Agtr1b* | CTTGCCCTGGCTGATTTAT | GCCGAAGCGATCTTACATAG |
| *Hdac1* | GACATGCCAAGTGTGTGGAGT | CACAGCTGTCTCGTAAGTCCAG |
| *Hdac2* | GGACAAGAGGACAGATGTTAAGG | GGGTTGTTGAGTTGTTCTGATTT |
| *Hdac3* | CCCCAGATTTCACGCTCCAT | TGAATCTGGACACTGGGTGC |
| *Hdac4* | CTACATCAGAGACCCAATGC | GTGACTGTCTCAGCTTCTTC |
| *Hdac5* | TCCCGTCCGTCTGTCTGTTA | GACATGCCATCCGACTCGTT |
| *Hdac6* | GCACGCTGTCTCATCCTACCT | CCCGAGTTTTCATCTTTTCTGTG |
| *Hdac7* | CTAGGGTCTCAGAACAGATCA | TCTCAAGTTCCCTGGGATAG |
| *Hdac8* | CAGCCAAGAAGGGGATGAGA | CTTCCCGTCAATCAGGCACT |
| *Hdac9* | GGAGGACACACTGGGACAAG | CCTGCTCCCCACATTCCATT |
| *Hdac10* | GTCCCTGCCCAATATCAC | GCTGTTCGGTTTGCCCTC |
| *Hdac11* | CTAAAGAGGCCATCAGGCGG | CACCACAACATCAGGCAGGT |
| **Mouse** |  |  |
| *Serpina3c* | CTGGGGCTCGTGATAACTGG | TCGAGTTGTGTCCCATTTTCTTT |
| *Agtr1a* | GTGGCCAGTGTCTTTCTT | CCGAAGCGATCTTACATAGG |
| *Agtr1b* | CTTGCCCTGGCTGATTTAT | GCCGAAGCGATCTTACATAG |
| *Gapdh* | AGGTCGGTGTGAACGGATTTG | TGTAGACCATGTAGTTGAGGTCA |
| *Glut2* | TCATTGCTGGACGAAGTGTATC | ACATTGGAACCAGTCCTGAAA |
| *Ppara* | CGTACGGCAATGGCTTTAT | GTCACTGTCATCCAGTTCTAAG |
| *Cpt1a* | CTCCGCCTGAGCCATGAAG | CACCAGTGATGATGCCATTCT |
| *Ehhadh* | ATGGCTGAGTATCTGAGGCTG | GGTCCAAACTAGCTTTCTGGAG |
| **Human** |  |  |
| *GAPDH* | ACAACTTTGGTATCGTGGAAGG | GCCATCACGCCACAGTTTC |
| *SERPINA3* | TCCATCTCGAGGGACTATAAC | GTGCCCTCCTCAAATACATC |
| *GR* (/*NR3C1*) | ACAGCATCCCTTTCTCAACAG | AGATCCTTGGCACCTATTCCAAT |
| *HDAC3* | CCTGGCATTGACCCATAGCC | CTCTTGGTGAAGCCTTGCATA |

**Supplementary images of western blot bands by Tanon 5200 Chemiluminescent Imaging System** **or Amersham ImageQuant ™ 800 System (Cytiva, Washington, D.C., USA)**

**Fig. 3e**
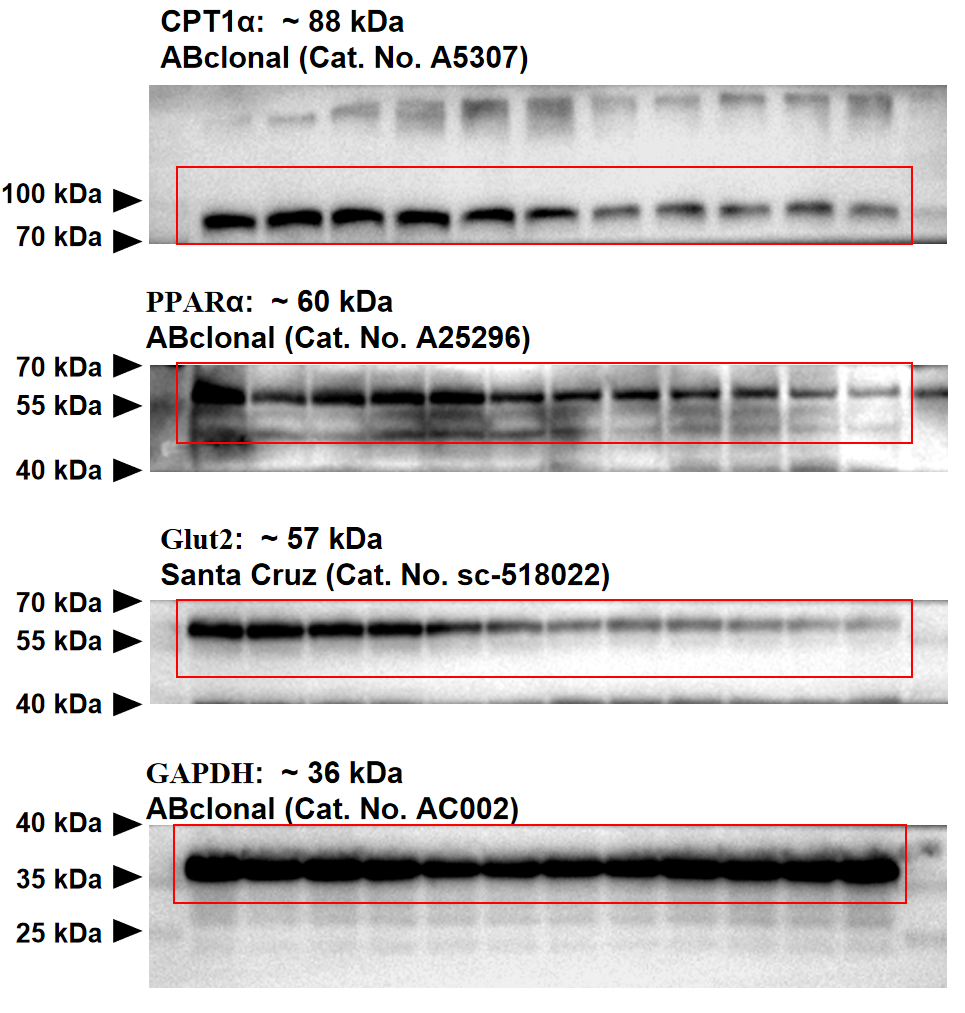


**Fig. 3g**


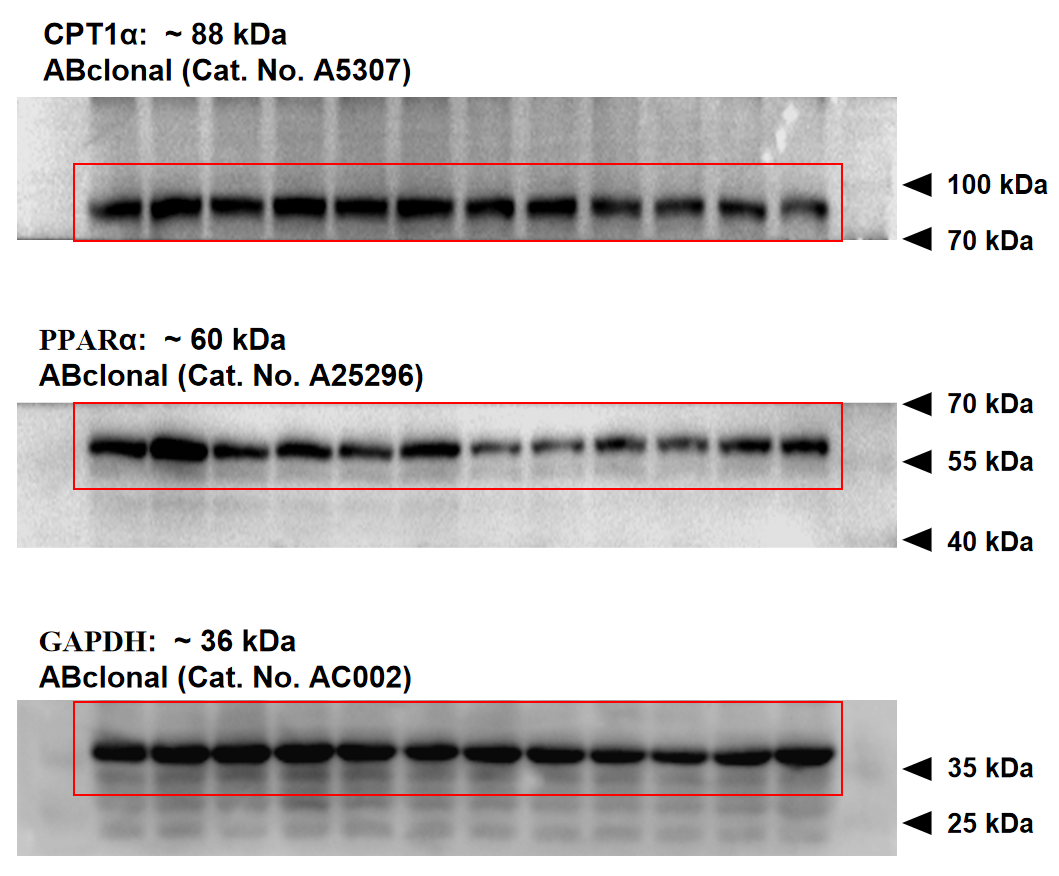


**Fig. 3i**
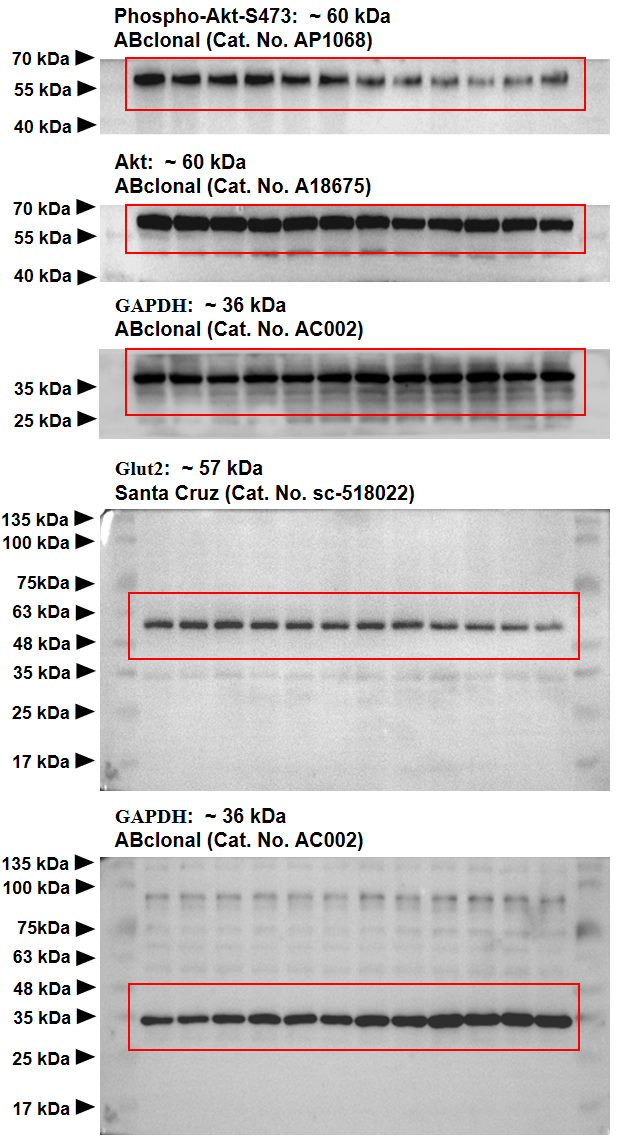


**Fig. 3k**


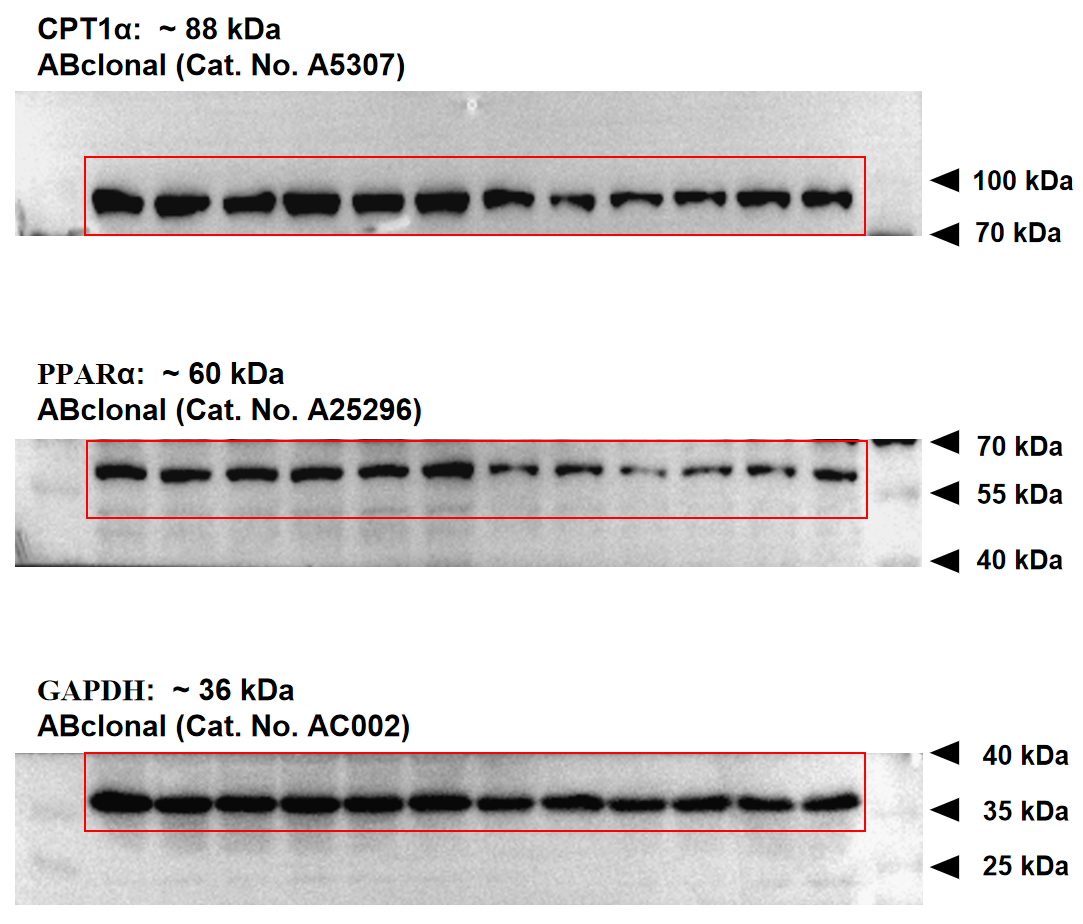


**Fig. 3m**


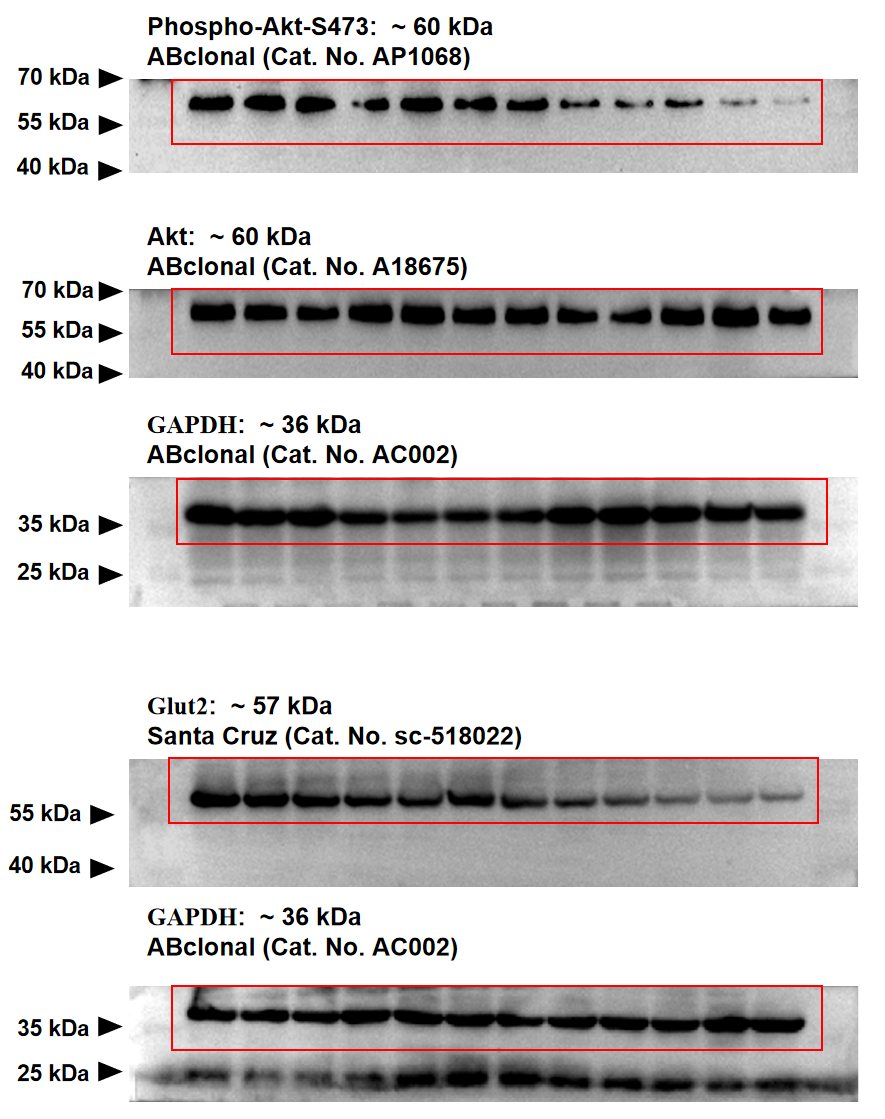


**Fig. 4e**


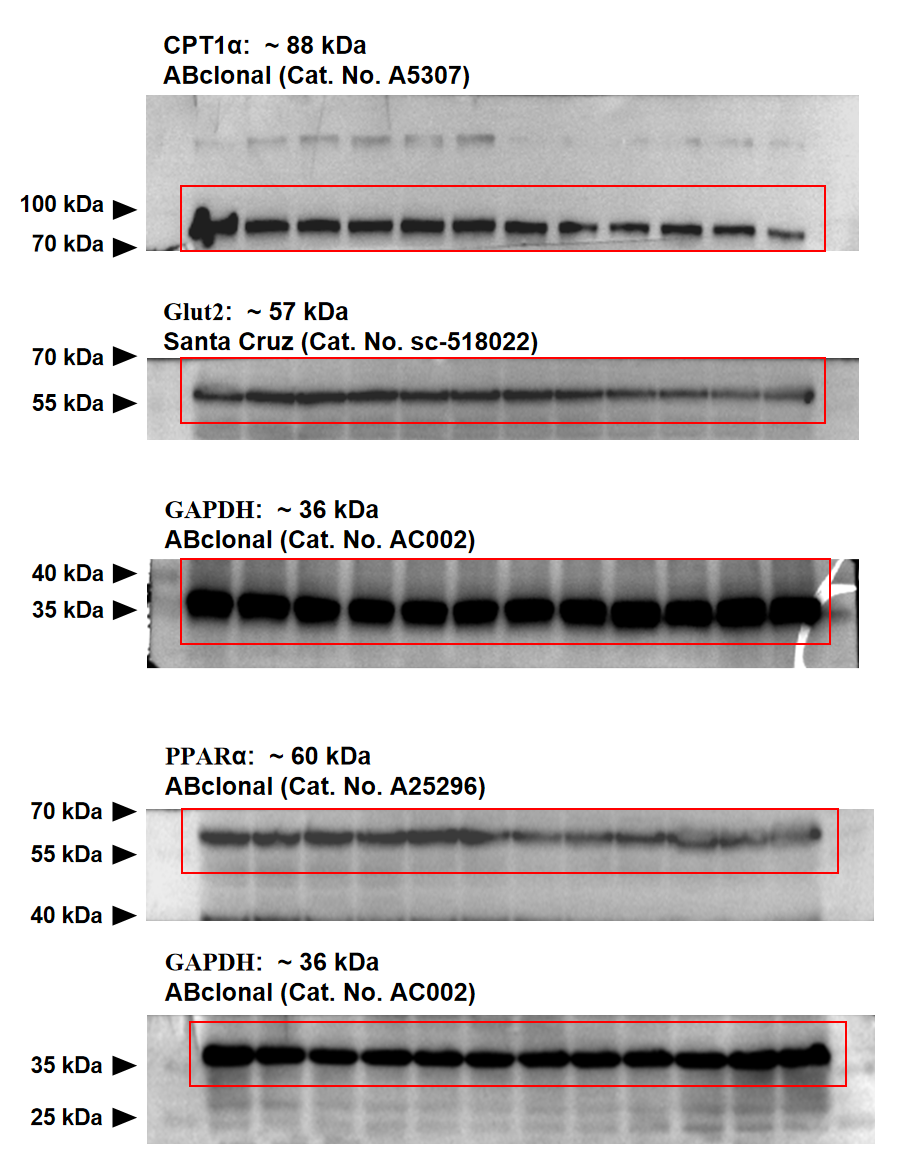


**Fig. 4g**
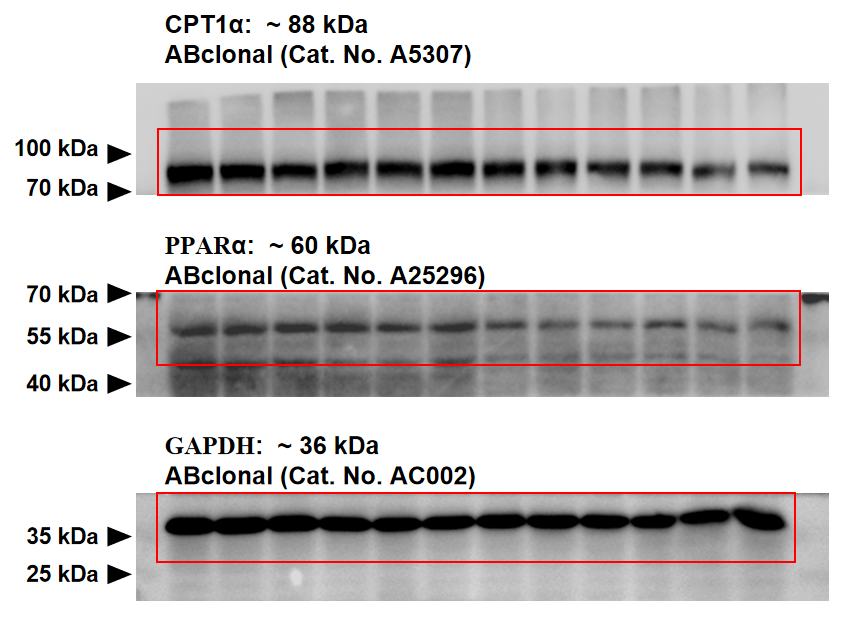


**Fig. 4i**
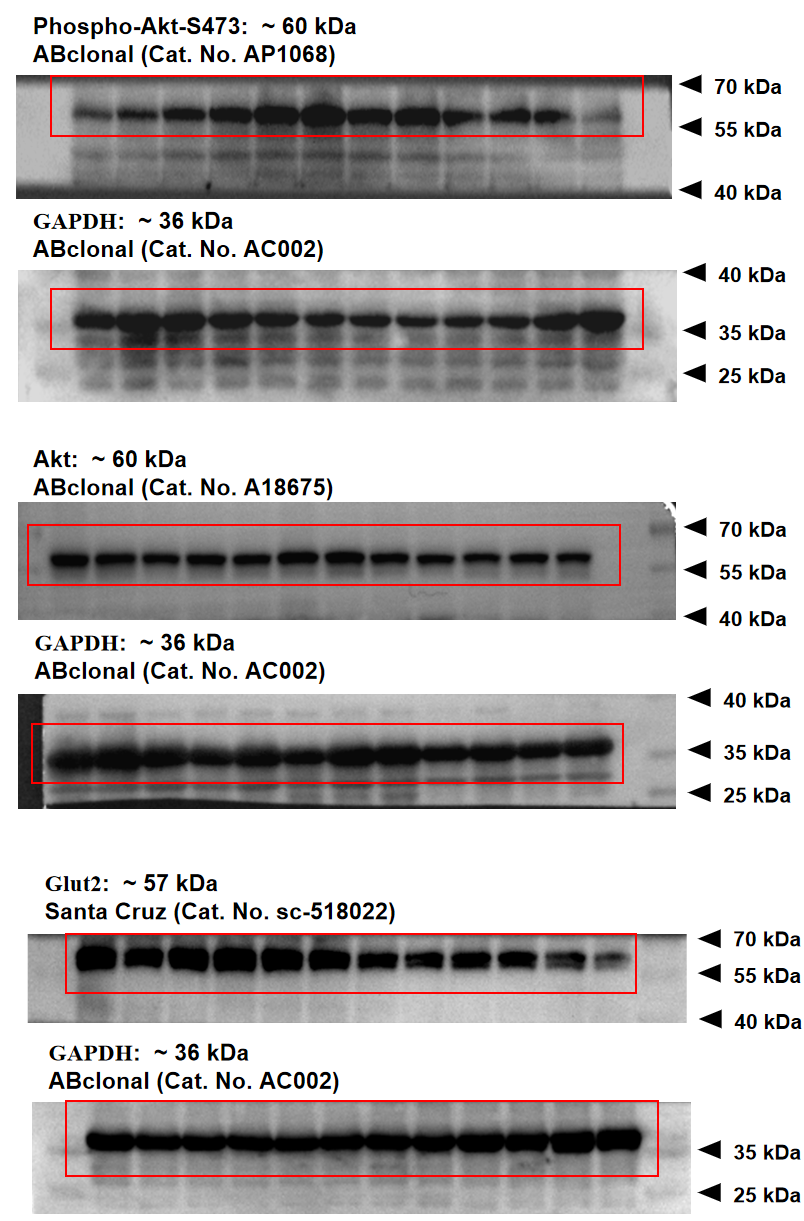


**Fig. 4k**
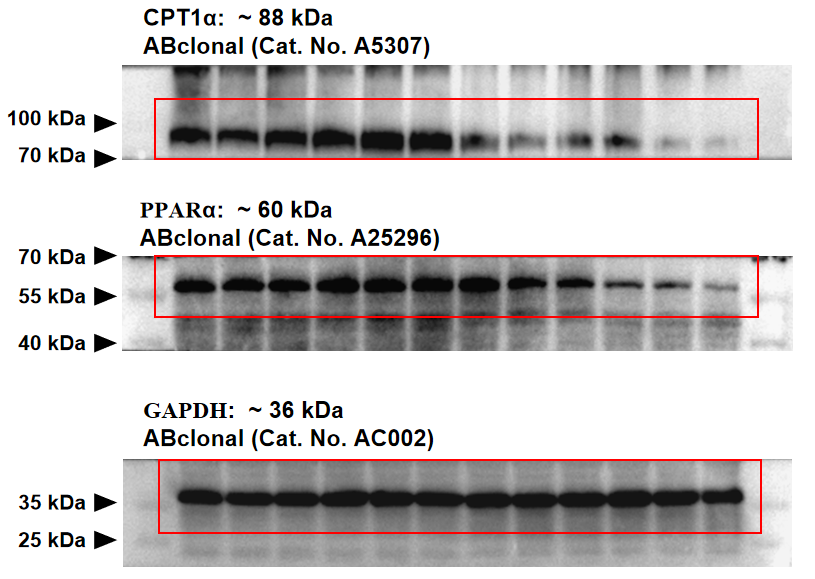


**Fig. 4m**
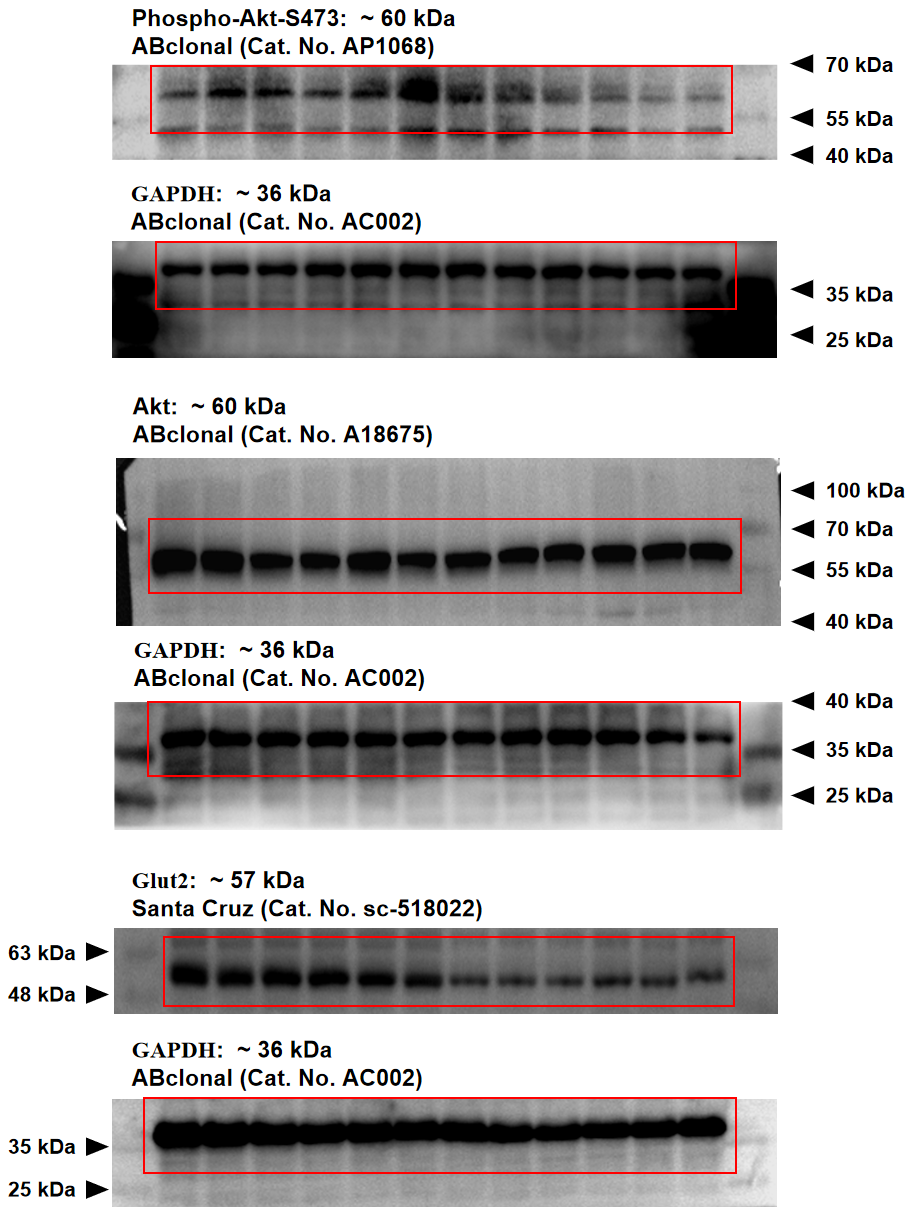


**Fig. 5e**


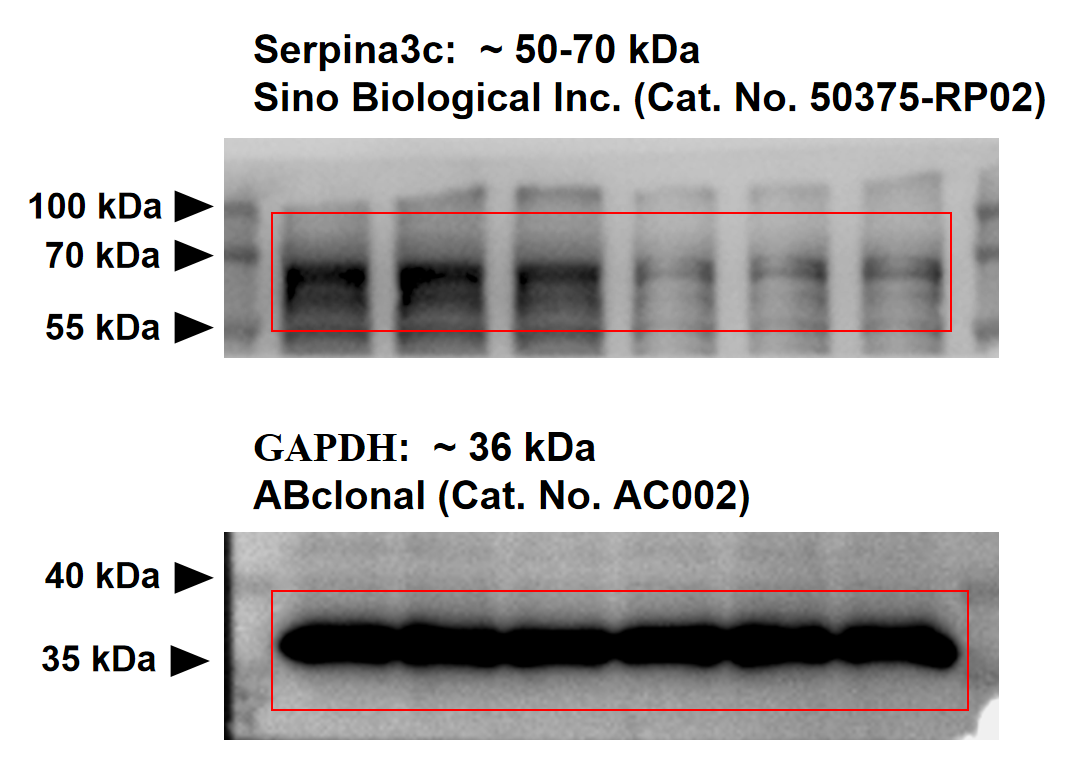


**Fig. 5i**
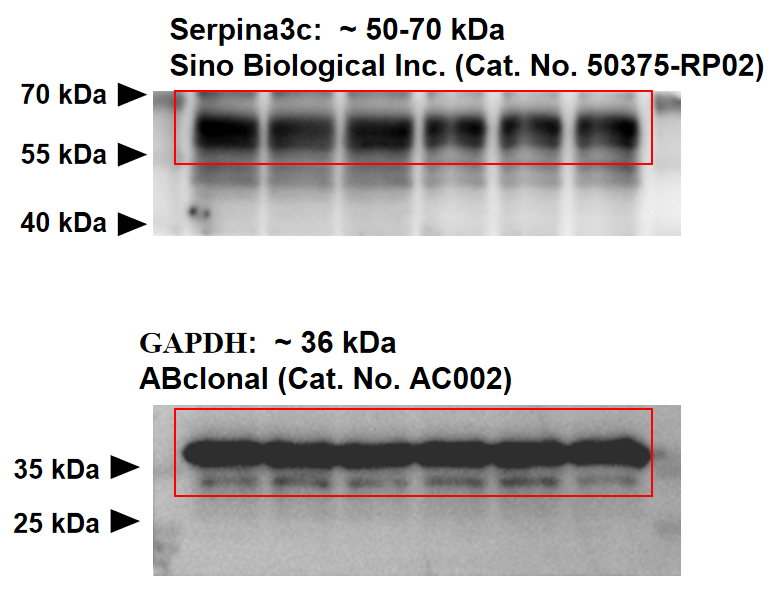


**Fig. 5r**


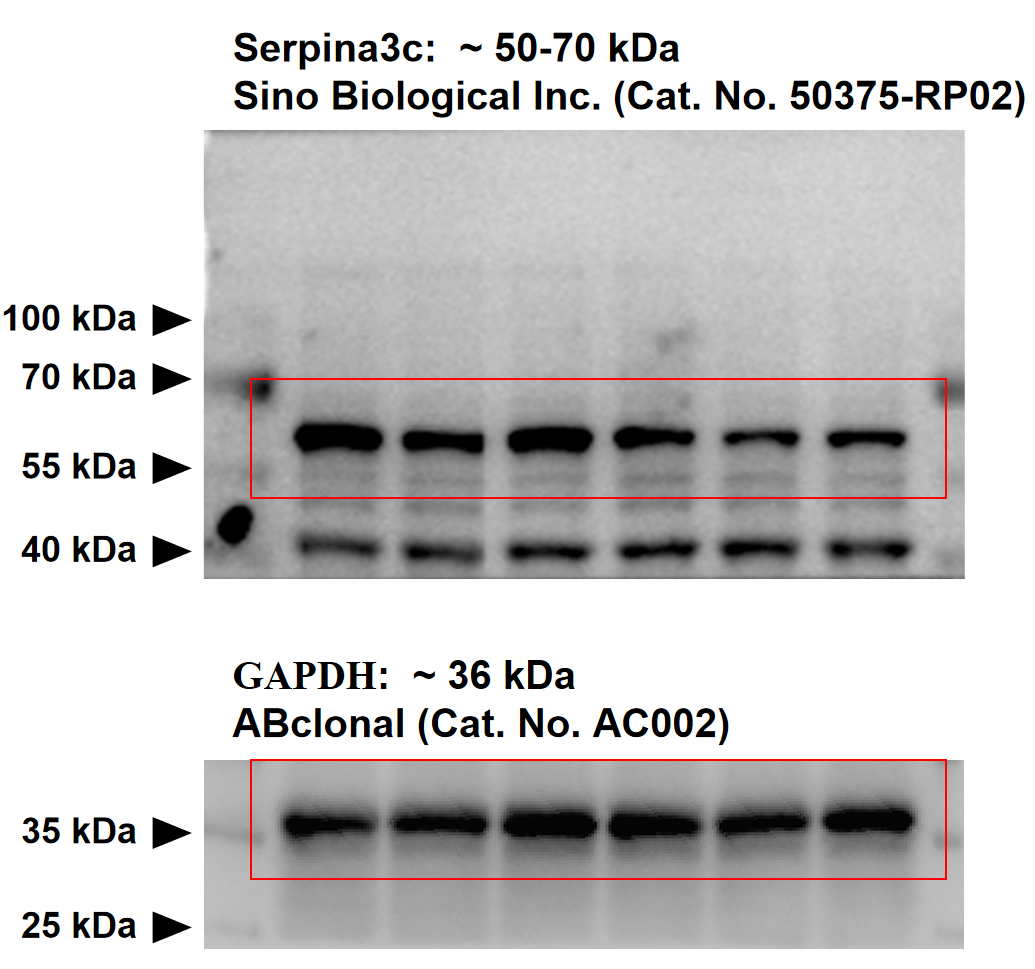


**Fig. 6i**
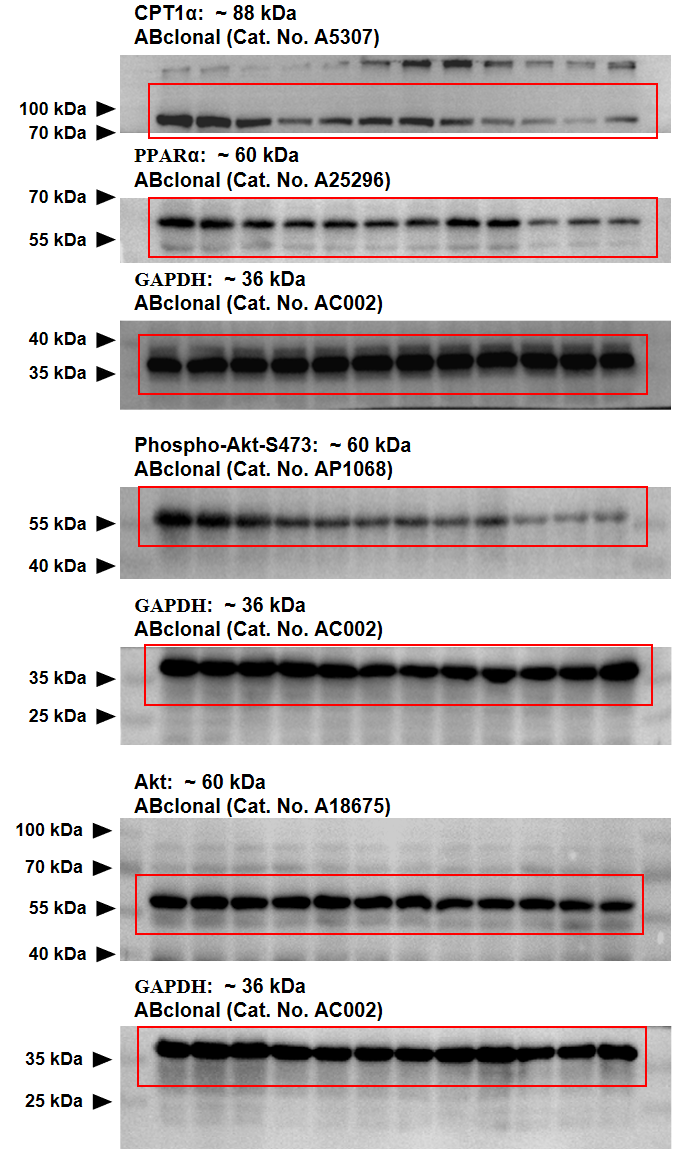


**Fig. 7a**
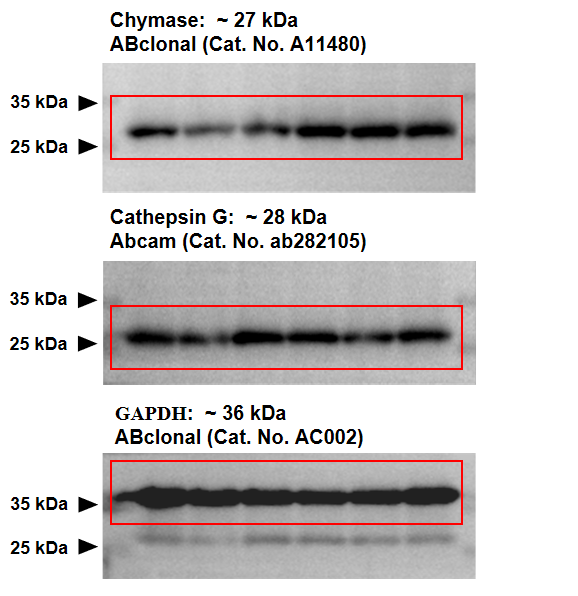


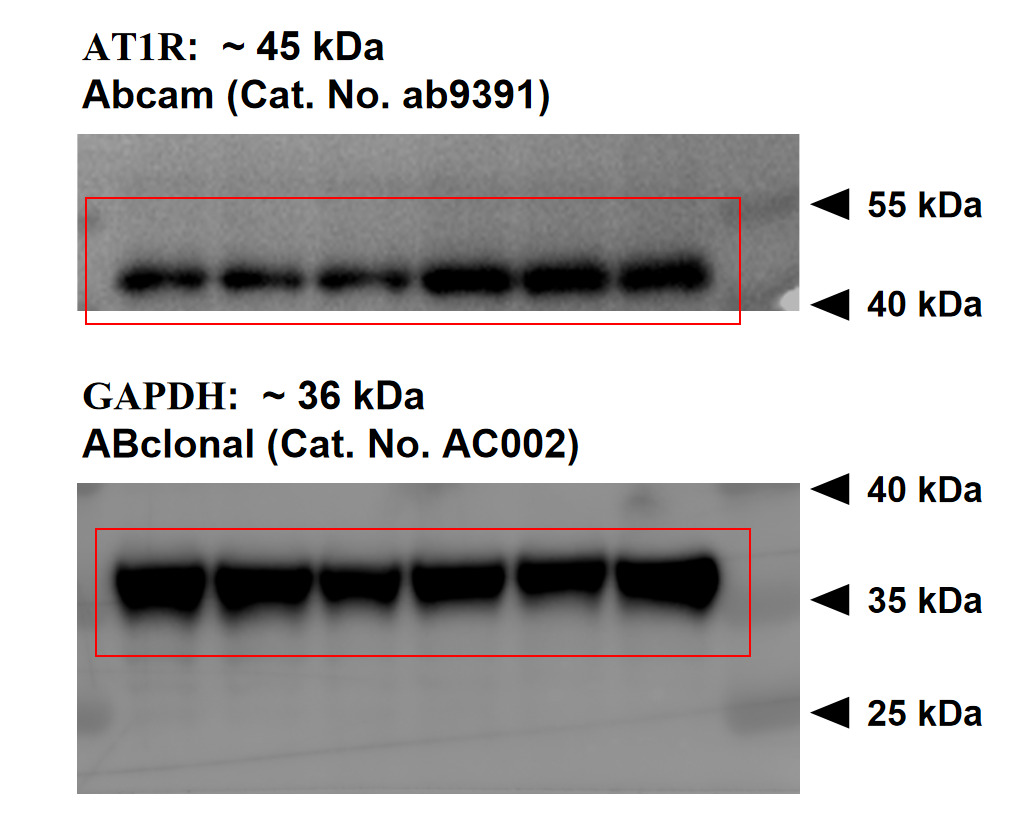


**Fig. 7e**


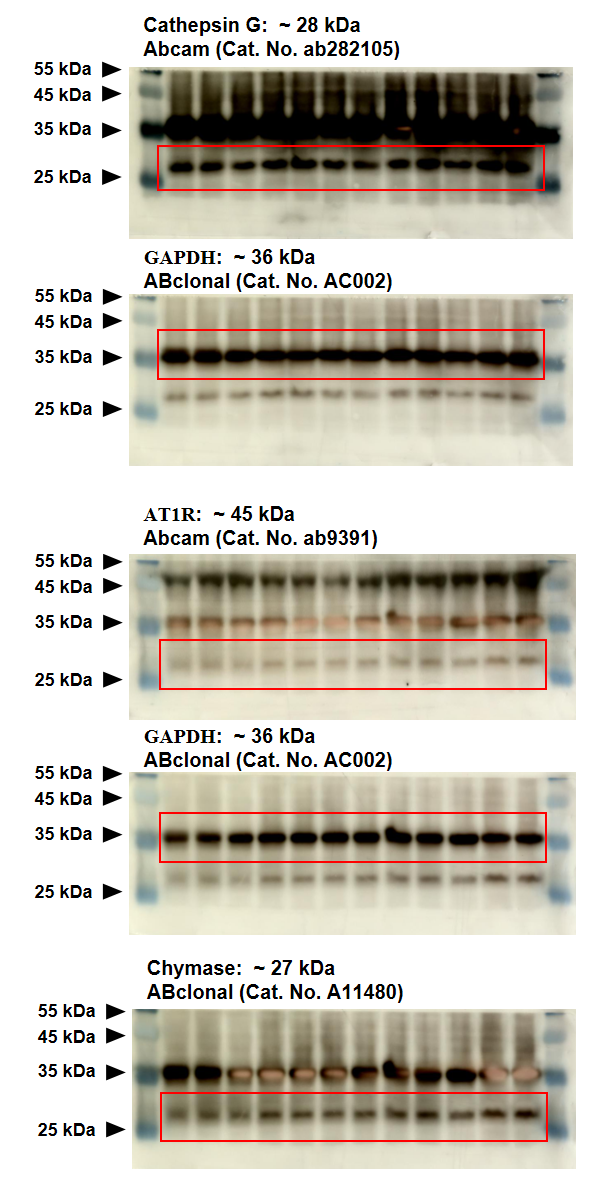


**Fig. 7i**
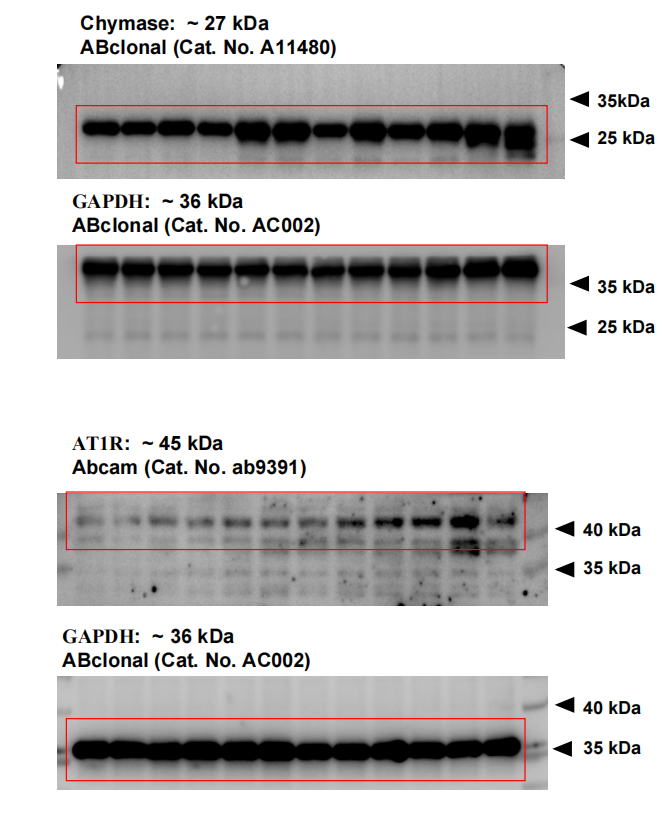


**Fig. 7m**


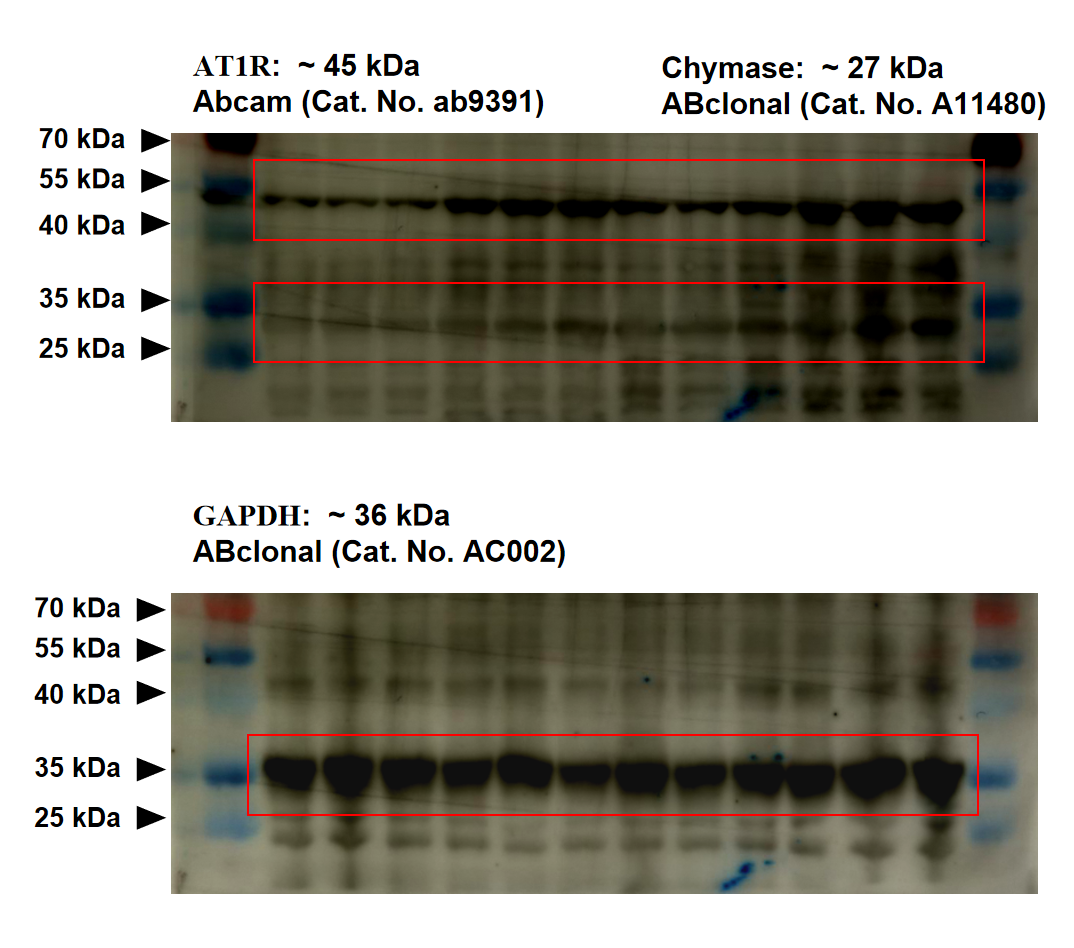


**Fig. 8a**


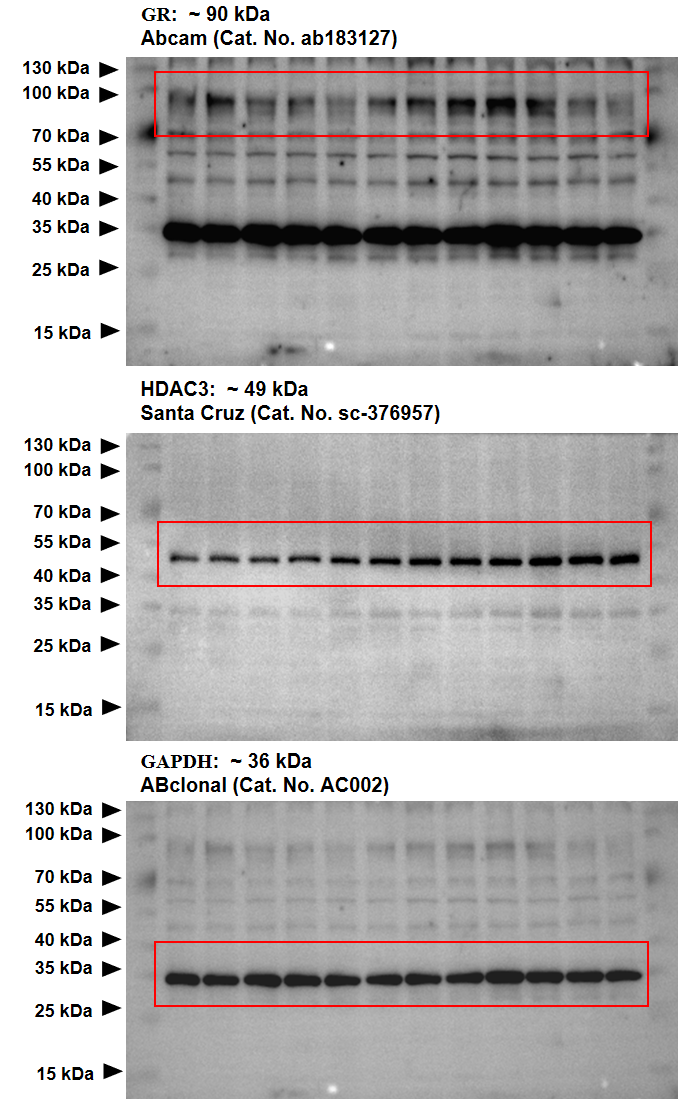


**Fig. 8a**


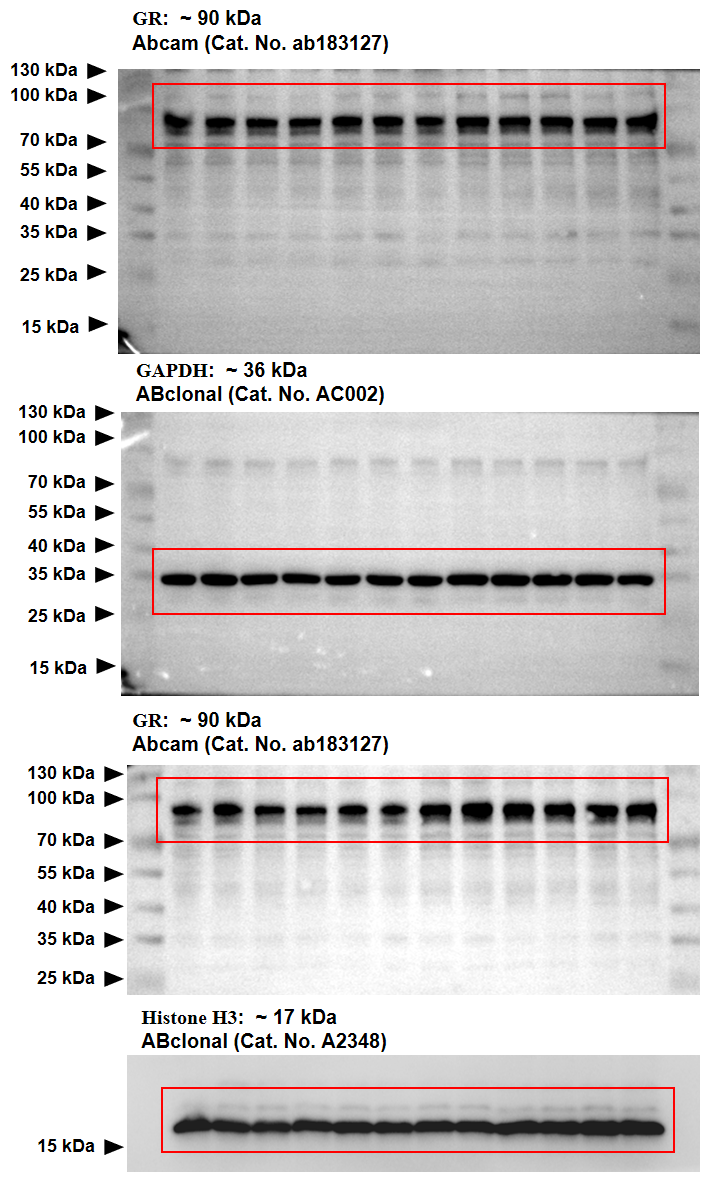


**Fig. 8h**


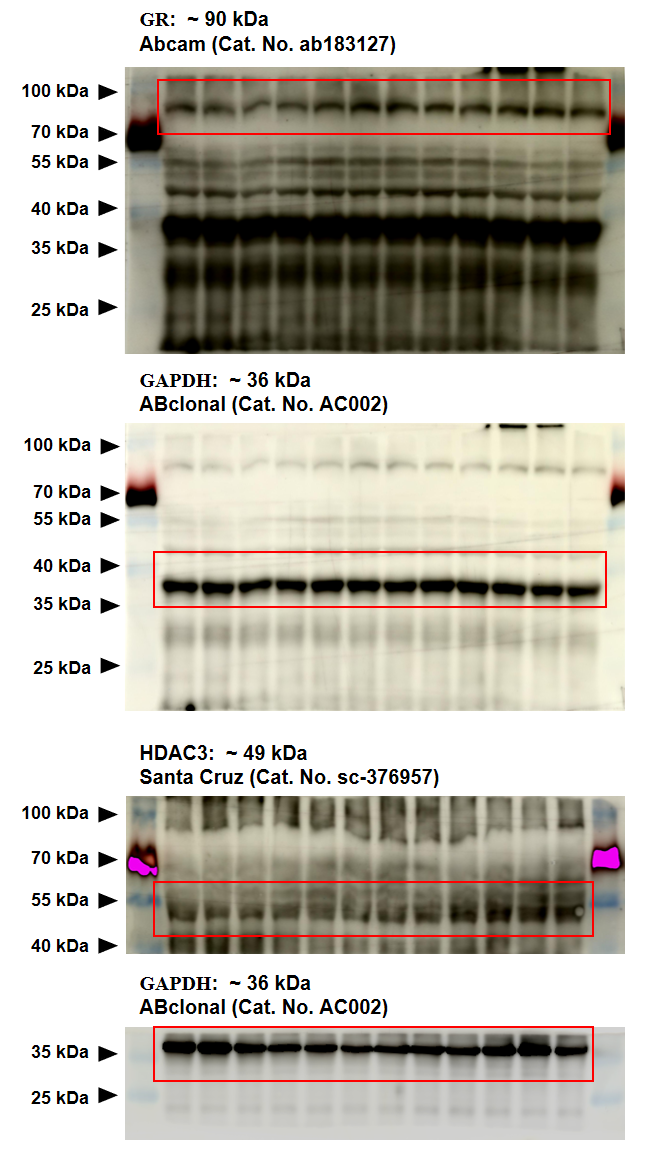


**Fig. 8h**


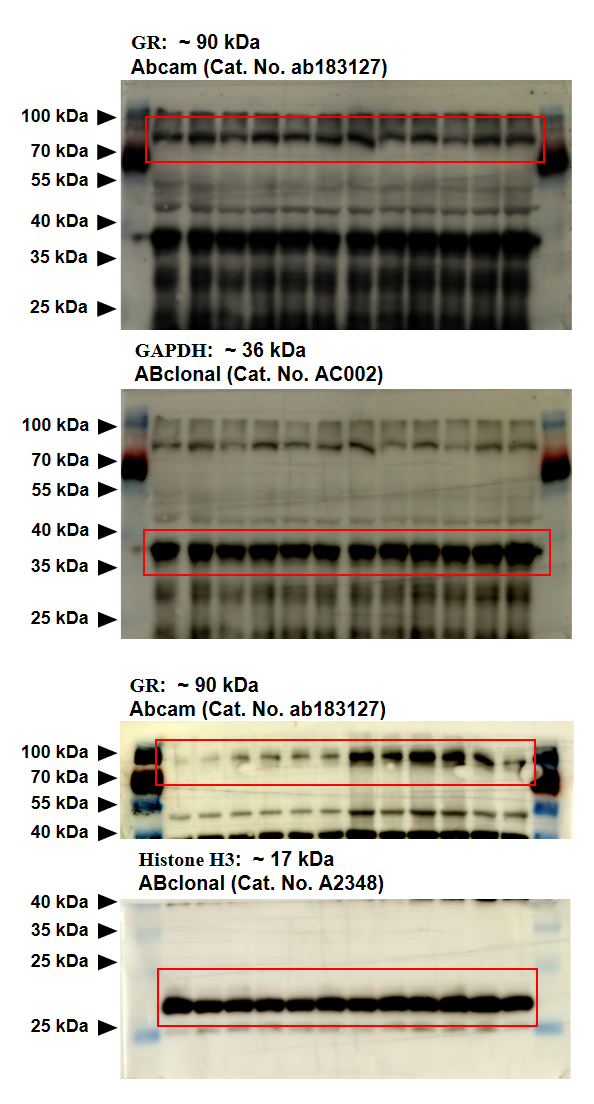


**Fig. 9a**
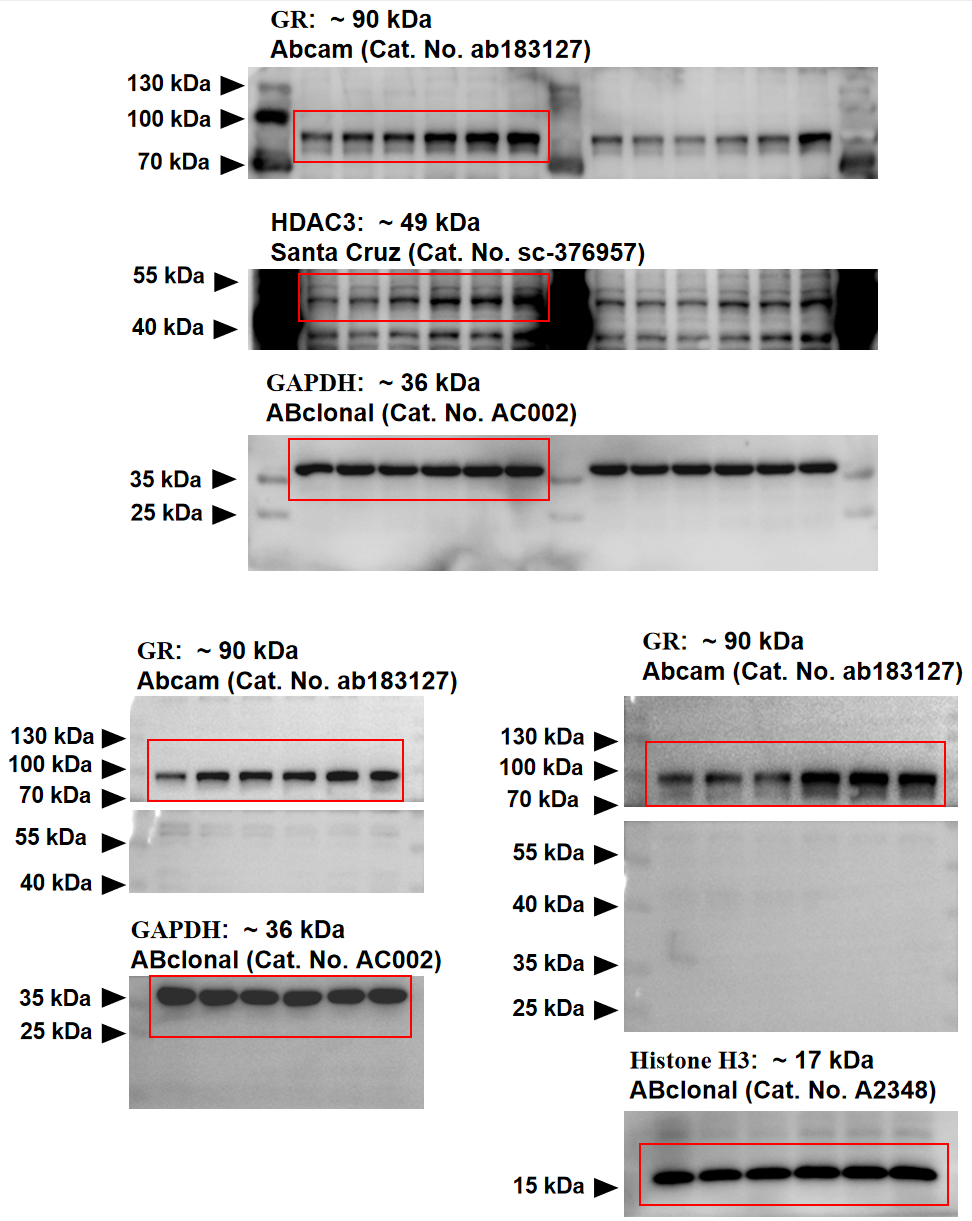


**Fig. 9f**


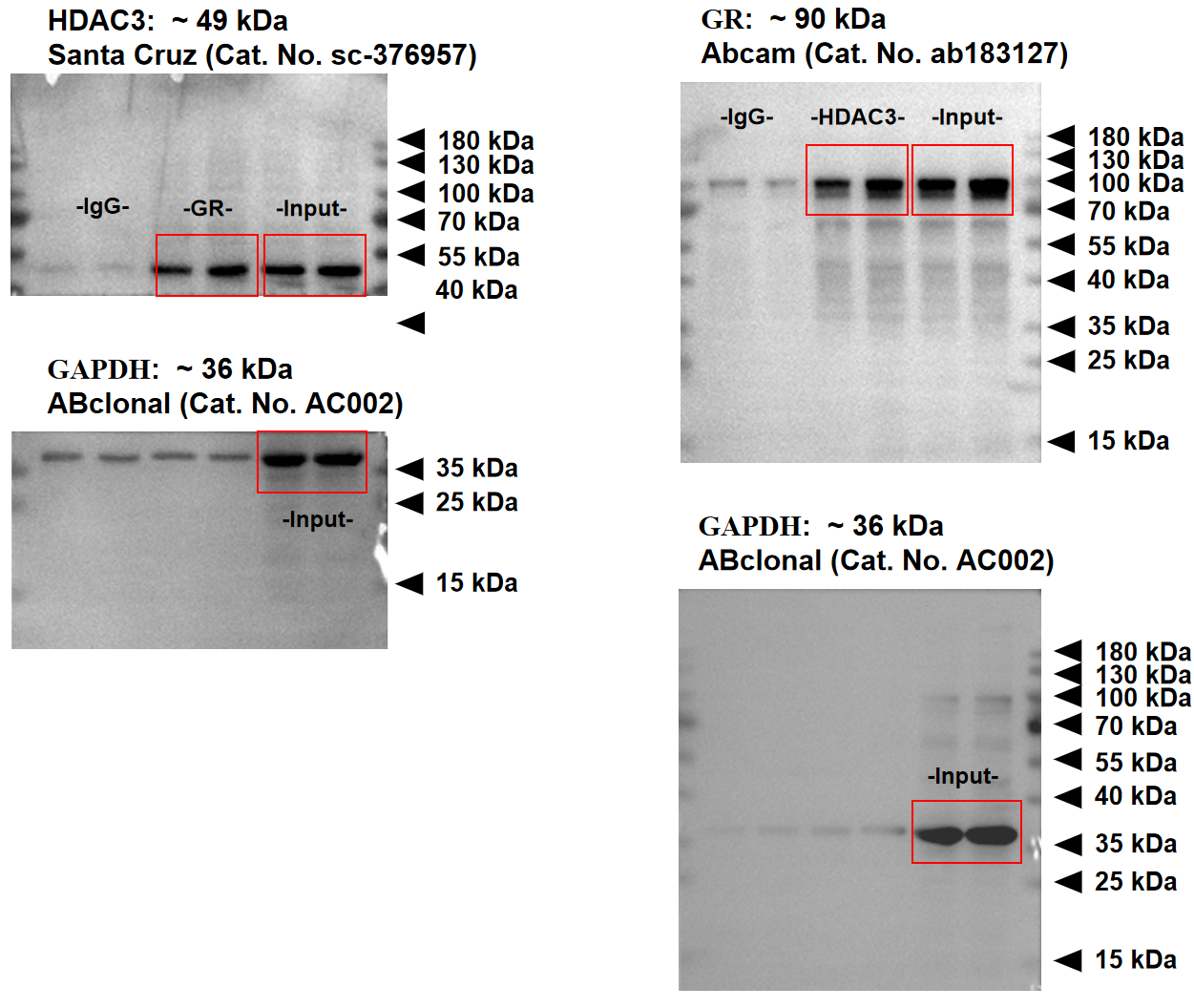


**Fig. 9k and 9q**
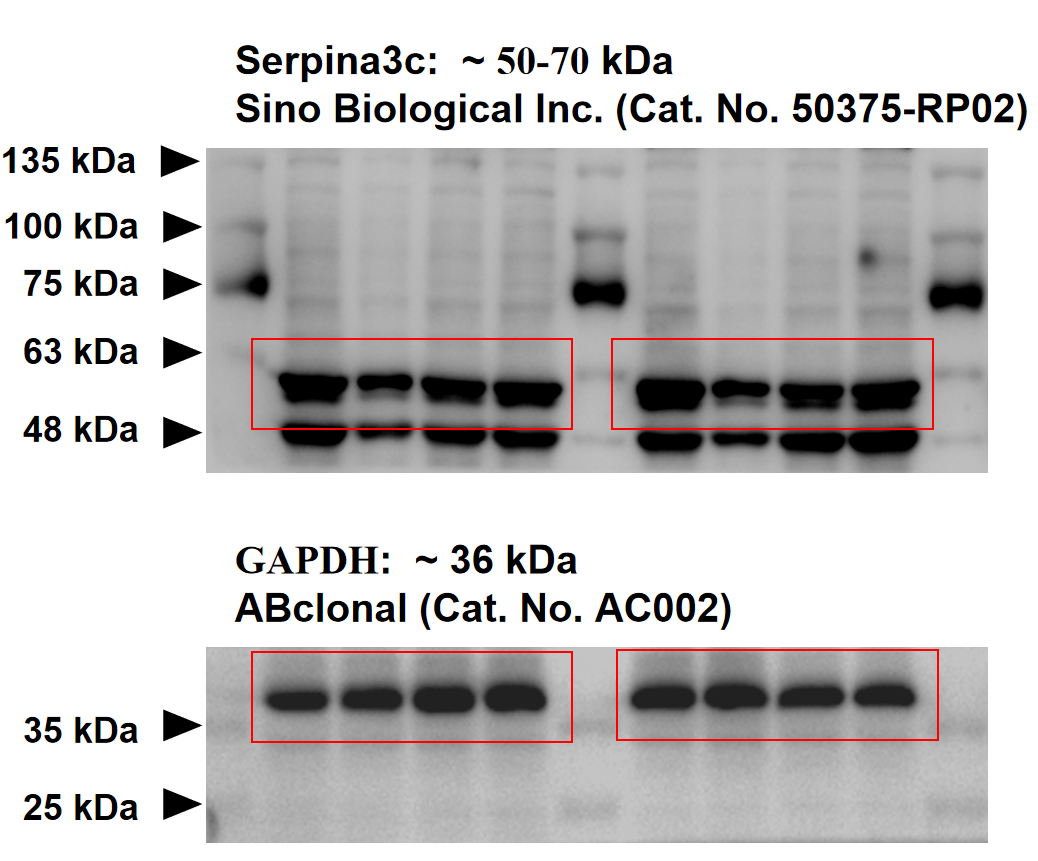


**Fig. 10a**
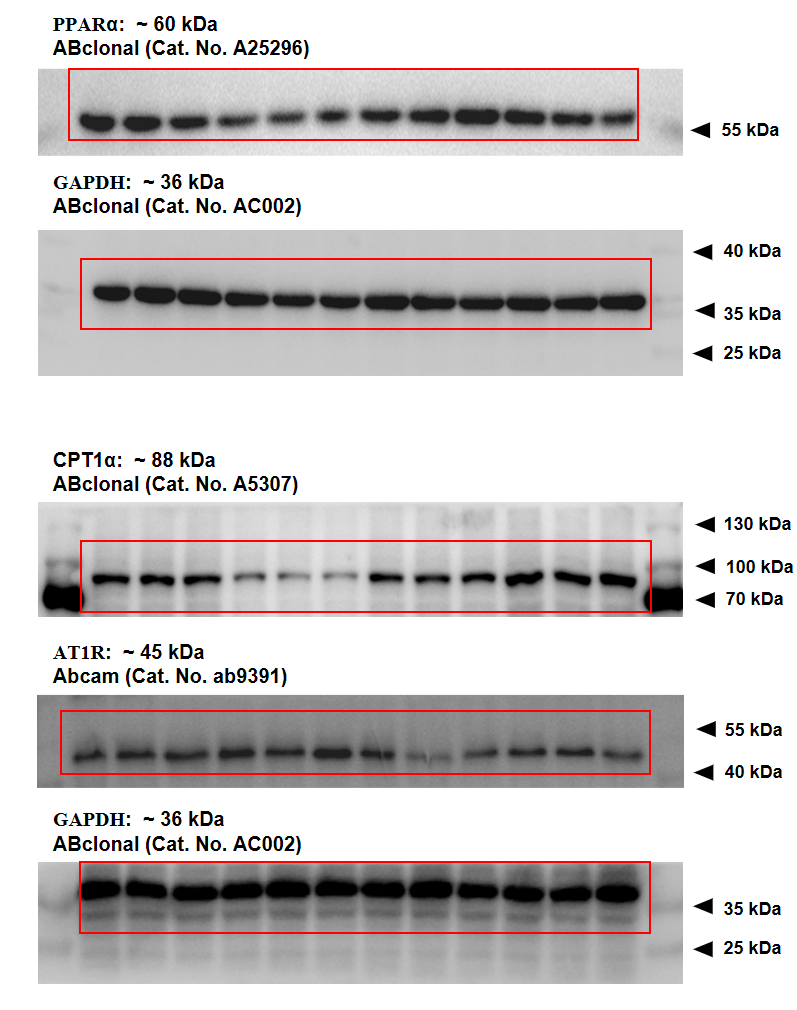


**Fig. 10a**
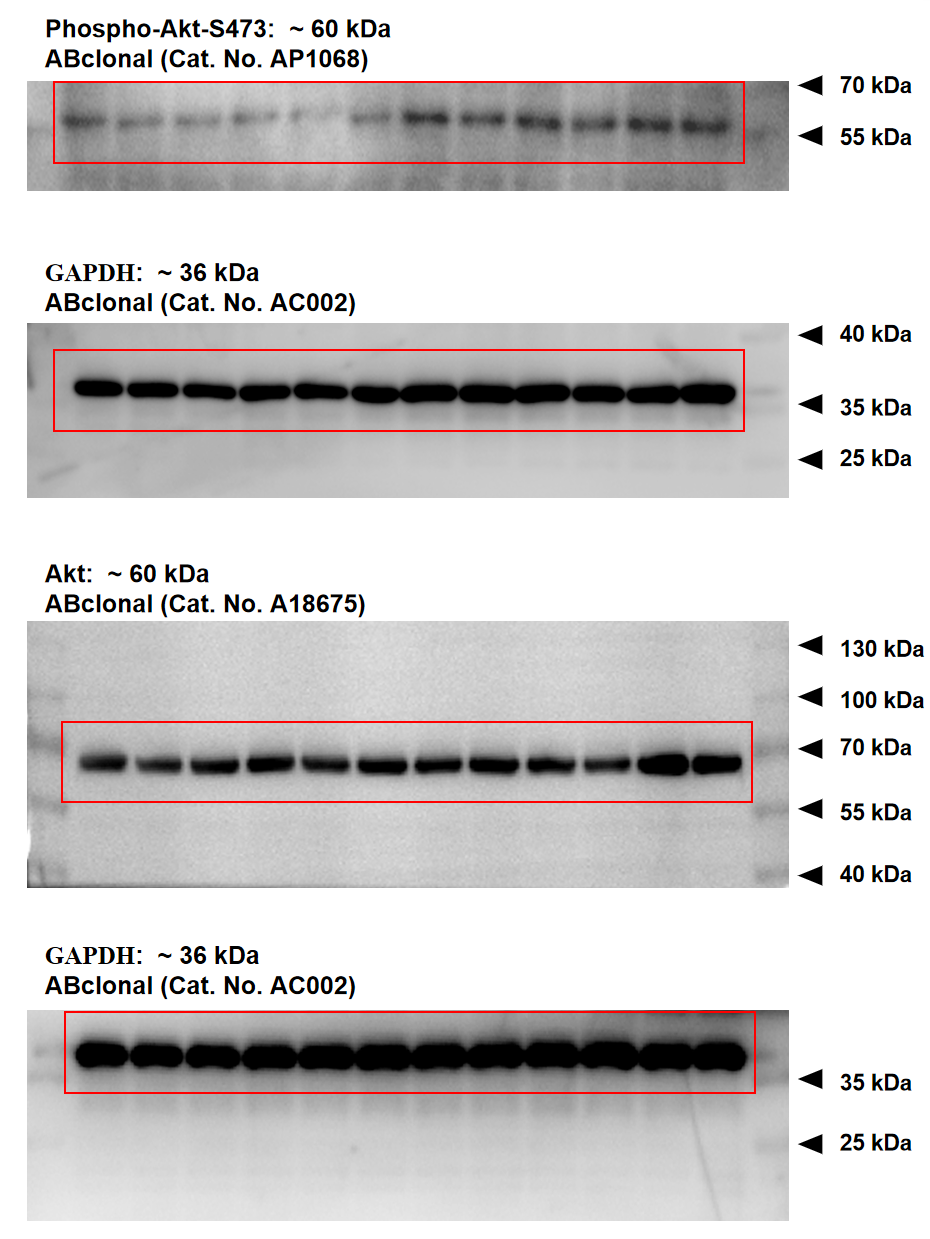


**Fig. 10a**
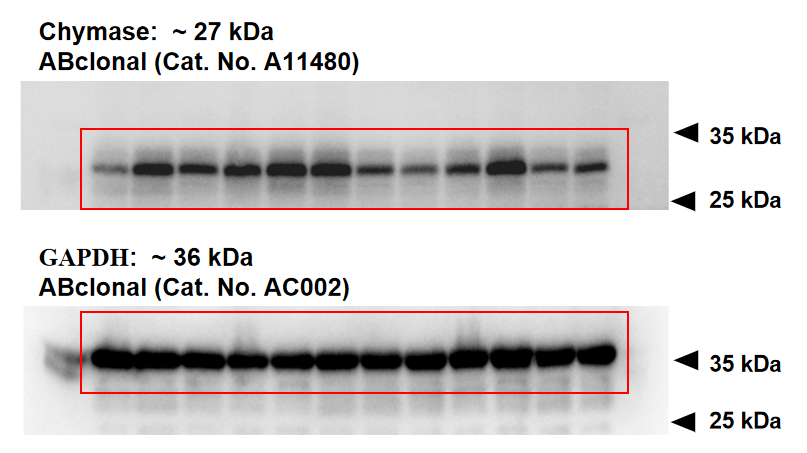


**Fig. 11a**


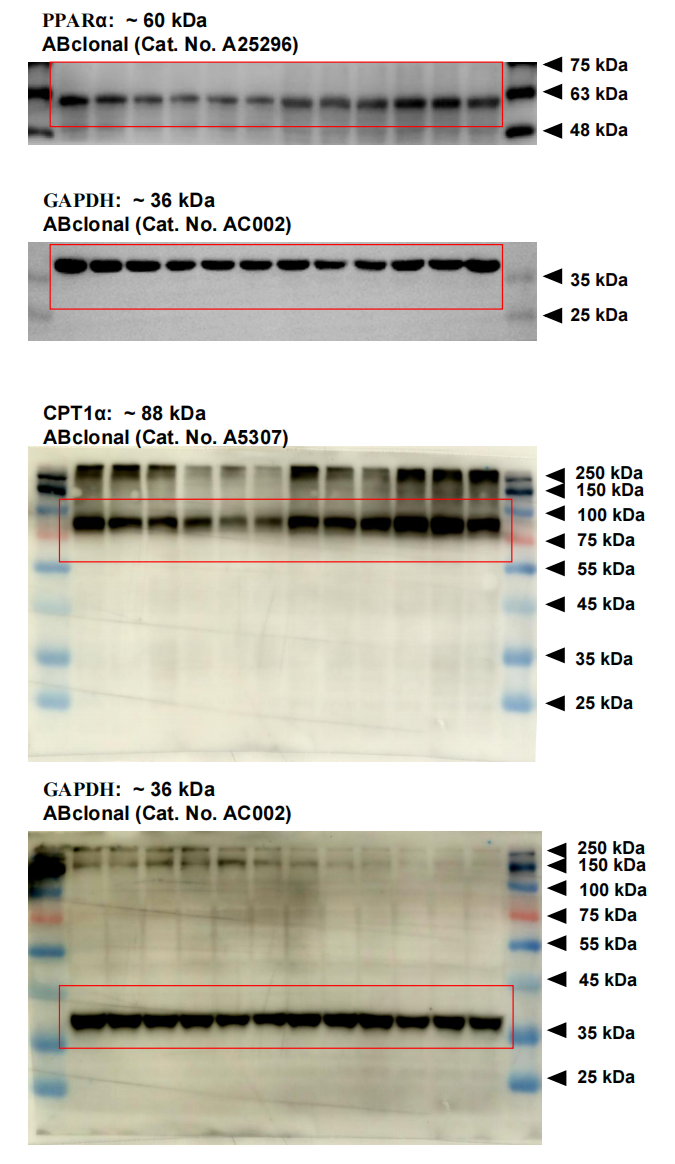


**Fig. 11a**


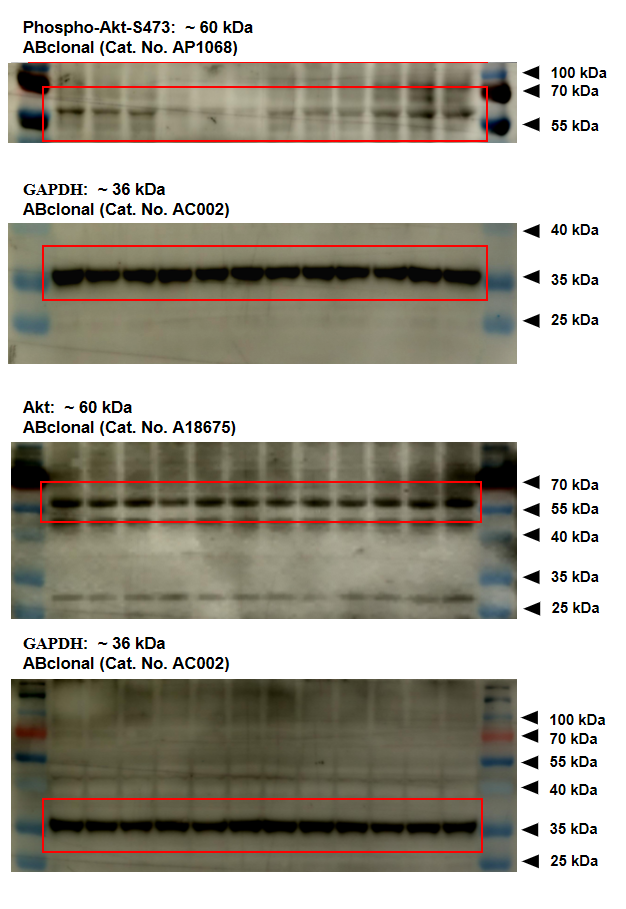


**Fig. 11a**


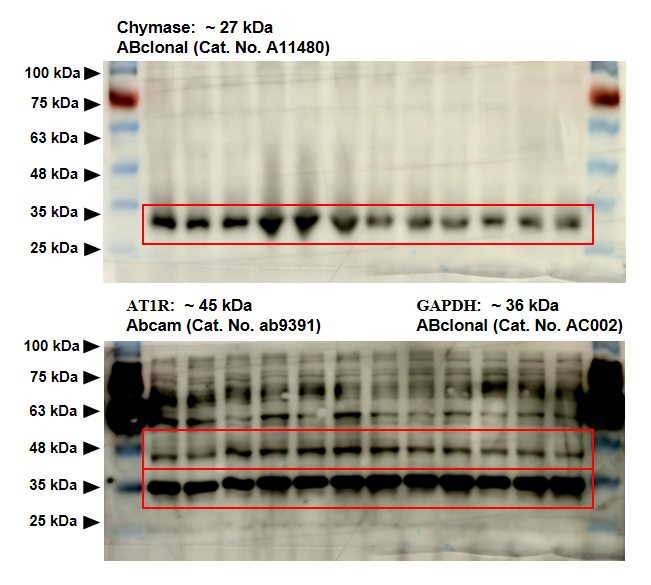


**Supplementary Fig. 8b**


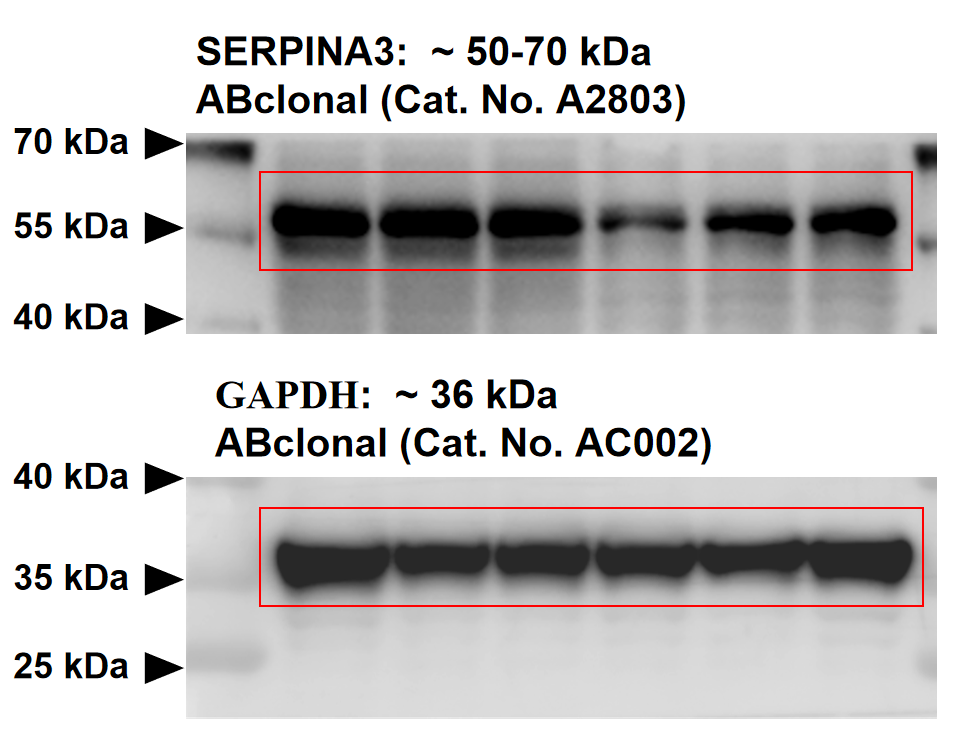


**Supplementary Fig. 10b**
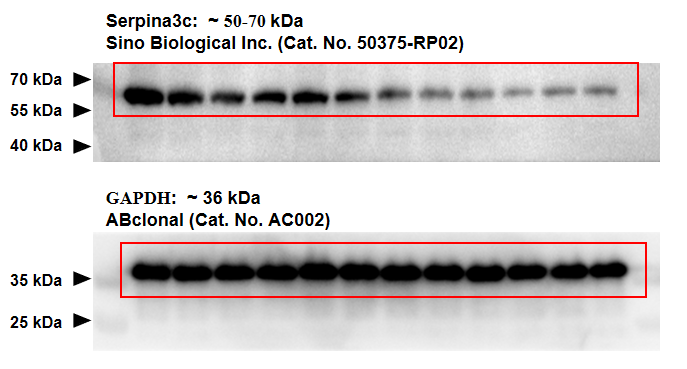


**Supplementary Fig. 10g**
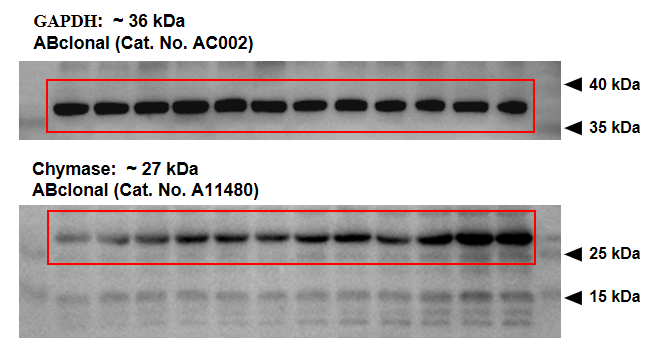


**Supplementary Fig. 11e**
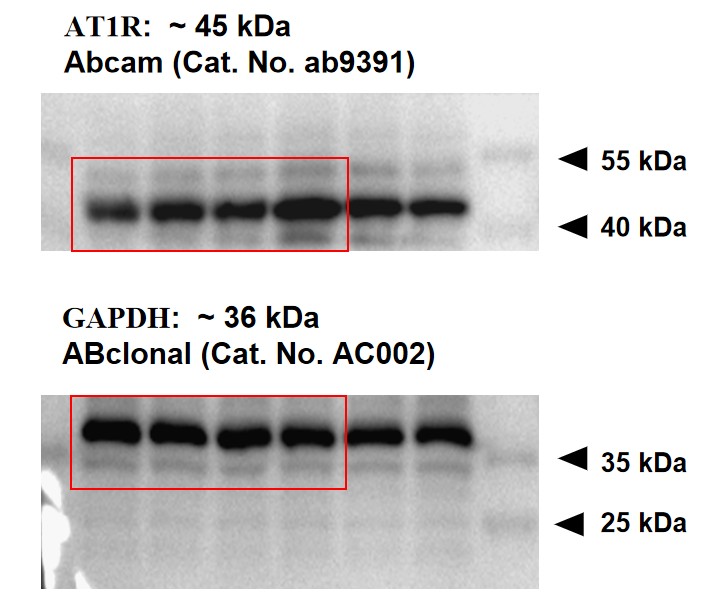


**Supplementary Fig. 12b**
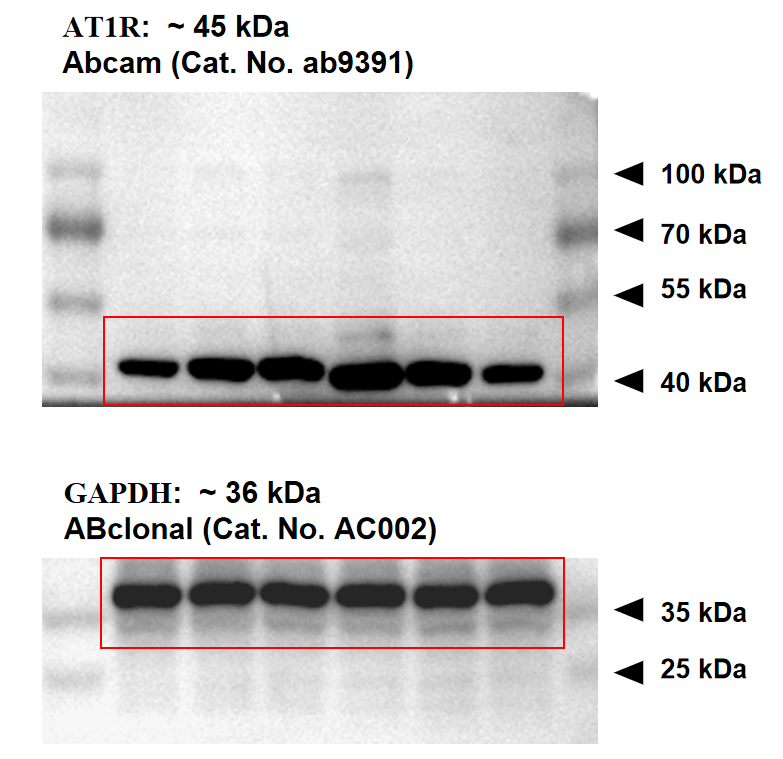


**Supplementary Fig. 16a**
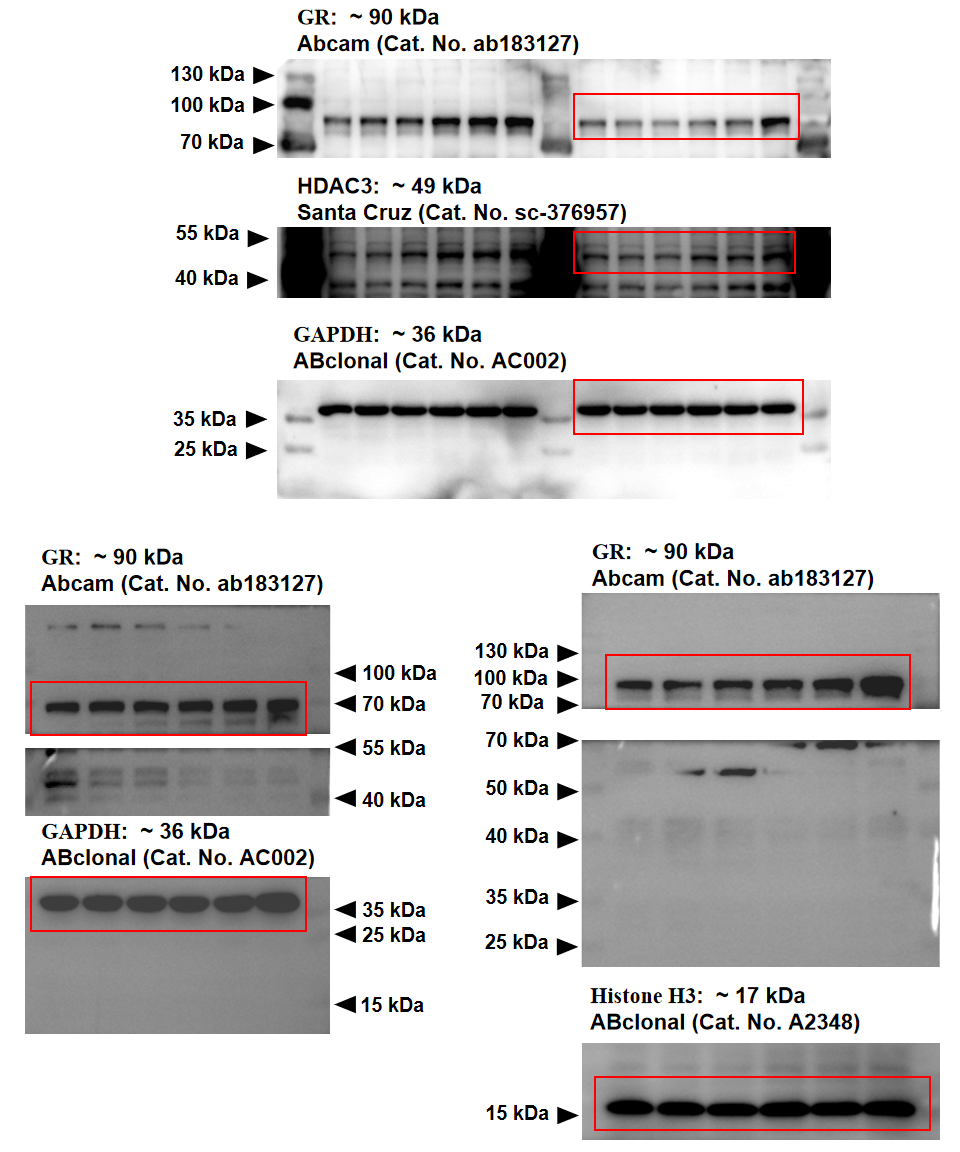


**Supplementary Fig. 16g and h**


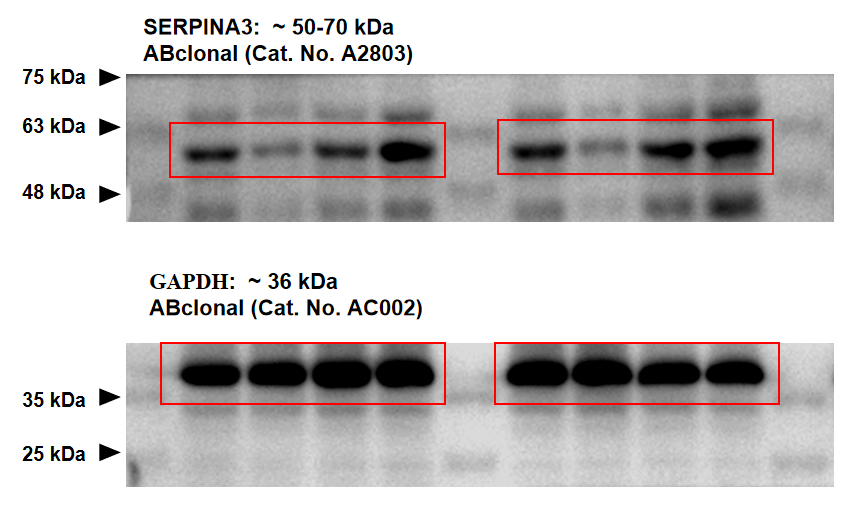

Supplement: Supplementary file 1 — Supplementary material [file 41392_2025_2569_MOESM1_ESM.docx]
